# Gold(I/III)-Phosphine Complexes as Potent Antiproliferative Agents

**DOI:** 10.1038/s41598-019-48584-5

**Published:** 2019-08-26

**Authors:** Jong Hyun Kim, Evan Reeder, Sean Parkin, Samuel G. Awuah

**Affiliations:** Department of Chemistry, University of Kentucky, 505 Rose St., Lexington, KY 40506 USA

**Keywords:** Medicinal chemistry, Coordination chemistry

## Abstract

The reaction of gold reagents [HAuCl_4_•3H_2_O], [AuCl(tht)], or cyclometalated gold(III) precursor, [C^NAuCl_2_] with chiral ((R,R)-(-)-2,3-bis(t-butylmethylphosphino) quinoxaline) and non-chiral phosphine (1,2-Bis(diphenylphosphino)ethane, dppe) ligands lead to distorted Au(I), (**1**, **2**, **4**, **5**) and novel cyclometalated Au(III) complexes (**3**, **6)**. These gold compounds were characterized by multinuclear NMR, microanalysis, mass spectrometry, and X-ray crystallography. The inherent electrochemical properties of the gold complexes were also studied by cyclic voltammetry and theoretical insight of the complexes was gained by density functional theory and TD-DFT calculations. The complexes effectively kill cancer cells with IC_50_ in the range of ~0.10–2.53 μΜ across K562, H460, and OVCAR8 cell lines. In addition, the retinal pigment epithelial cell line, RPE-Neo was used as a healthy cell line for comparison. Differential cellular uptake in cancer cells was observed for the compounds by measuring the intracellular accumulation of gold using ICP-OES. Furthermore, the compounds trigger early – late stage apoptosis through potential disruption of redox homeostasis. Complexes **1** and **3** induce predominant G1 cell cycle arrest. Results presented in this report suggest that stable gold-phosphine complexes with variable oxidation states hold promise in anticancer drug discovery and need further development.

## Introduction

Gold-based probe development and drug discovery remain a burgeoning area of biological research and treatment for disease indications such as cancer^1–5^, arthritis^6–9^, and microbial infection^10,11^ following the FDA approval of tetra-O-acetylglucose-1-thiolgold(I) triethylphosphine complex (auranofin). Exploring the Au(I) and Au(III) chemical space has given rise to enormous diversity of gold compounds of biological relevance, influenced by creative ligand design^12–16^. Despite effective clinical and preclinical treatment of cancer and rheumatoid arthritis by gold complexes such as auranofin, the molecular basis of drug action remains unclear for gold(III) phosphine compounds present in this report. Years of research implicates a number of disease targets including: (i) proteasome-associated deubiquitinases^6–9^; (ii) thiol-rich enzymes such as thioredoxin and glutathione reductase^17–20^; (iii) thiol-dependent proteases^21^; iv) autophagy induction^22^; and superoxide/oxyradical ion generation^23^.

Auranofin, which is under clinical and preclinical investigation for the treatment of a variety of cancers including leukemia^24,25^ and ovarian malignancies^26–28^ as well as microbial infections^29–31^ is a phosphinogold complex. This has accelerated the development and discovery of several gold-phosphine complexes for therapeutic applications. Gold(I)-phosphine anti-cancer complexes have been identified to trigger apoptosis by targeting the mitochondria and inhibiting thioredoxin reductase^32–34^. Structural diversity of gold complexes bearing phosphine ligands have important implications for anticancer activity and probe development^20,35^. Work by Berners-Price *et al*. demonstrated the anticancer effect of gold-phosphine complexes and have also tried to improve the *in vitro* and *in vivo* efficacy of this class of compounds^2,5,36–42^. Gold complexes bearing dithiocarbamate^43–45^ and triorganophosphine ligands^33,46^ of the type [(R_3_P) Au(S_2_CNR_2_)] display anticancer activity across a panel of cancer cells including ovarian cancer cells^47^. Recently, Darkwa and co-workers synthesized dinuclear phosphinogold(I) complexes bearing varied phosphine ligands including triphenylphosphine, and diphenylphosphino-alkanes and dithiocarbamates of the type [Au_2_Cl_2_(dppe)] and evaluated their anticancer activity^47^. The complexes displayed broad spectrum of activity in a number of cancer cell lines. Additionally, the anticancer activity of phosphinogold(I) complexes bearing thioglucose ligands as in the case of auranofin show higher potency than their thiolate counterparts even in cisplatin resistant cells. For example, the P – Au – S structural motif is prevalent in a number of gold-phosphine complexes such as the lupinylsulfide (OmS) or sulfanylpropenoate (sppa)^48^ containing phosphinogold(I), [AuOmS)_2_(Ph_2_P(CH_2_)_2_PPh_2_] or[Au(PPh_3_)(sppa)], respectively and they exhibit good anticancer activity^49^. Improving the biological activity of gold-phosphine complexes require ligand tuning that expand diversity, lipophilicity, physiological stability, and high selective cytotoxicity in cancer cells over normal cells^50,51^.

Whereas a lot of work has been conducted with linear phosphinogold(I), its high oxidation state counterpart gold(III) needs further exploration. Recent advancement of cyclometalated gold(III) in anticancer development show promising results^1,52–55^. These ligands impart strong σ-donating character to the gold center for stability and offer the possibility of different ligands around the metal center, given its square-planar geometry^56^. Che and co-workers showed that dinuclear cyclometalated gold(III) phosphine, [(C^N^C)_2_Au_2_(μ-dppp)]CF_3_SO_3_)_2_ inhibit hepatocellular carcinoma *in vivo* by inducing ER stress^57^. There still remains the need to expand the structural diversity of gold-phosphine complexes by designing new gold(III)-phosphine complexes.

Another important feature of ligands in the context of biological efficacy is chirality, since they possess the property to tune substrates to respective biological targets for improved target engagement that may be elusive for non-chiral ones. The use of chiral ligands in gold drug discovery remain largely unexplored. Incorporating chiral ligands into gold(I) or gold(III) complexes will expand the chemical space to further opportunities in medicinal inorganic chemistry.

In this report, we synthesized gold(I) complexes bearing chiral or achiral phosphine ligands and in addition mononuclear (C^N)-cyclometalated gold(III) bearing chiral or achiral phosphine ligands. The complexes display potent cytotoxic activity in different cancer cell lines by triggering apoptosis through ROS induction. The study establishes the need for a broader scope of gold complexes for cancer therapy.

## Results and Discussion

### Rationale and approach

Stabilizing the gold metal center for biological utility remains an important aspect of metallodrug discovery. Gold compounds possess high redox potential (i.e. Au^+3^ + 2e^−^ → Au^+1^ *E°* = 1.41 V and Au^+1^ + e^−^ → Au_(s)_ *E*^*o*^ = 1.69 V)^58^. This is due to relativistic effects. Often, strong σ-donating ligands such as phosphines and N-heterocyclic carbenes are used to improved stability of gold complexes against rapid reduction^59^. The polarizable nature of gold, owing to its loosely held electrons facilitates its coordination to soft Lewis bases such as phosphorus, sulfur containing ligands. Linear gold(I) complexes are limited by their low coordination number. On the other hand, gold(III) compounds largely exist in a square-planar configuration and provides increased number of coordination sites. This can be harnessed for the development of anticancer agents bearing multidentate ligands with enhanced stability and activity. In addition the use of chiral phosphine ligands in gold scaffolds and their effects on geometry remains underdeveloped. A study that examines the effect of oxidation states of compounds bearing phosphine ligands will be beneficial. We envisioned the investigation of gold(I)/(III)-phosphino complexes as potential anticancer agents to offer preliminary structure-activity insights of gold-based metallodrugs. These gold(I)/(III) complexes could interact with biological targets, such as proteins, in a similar fashion to reported gold-phosphino complexes but with optimal kinetic lability to avoid premature deactivation. Within this context, work that studied the anticancer potential of gold-phosphino complexes offered impetus that the complexes synthesized in our laboratory will be active against cancer cells *in vitro*. Furthermore, the distorted gold(I) with phosphine ligands as well as gold(III) complexes bearing cyclometalated and phosphine ligands could exhibit the needed stability for effective therapeutic response, even in drug-resistant cancer.

The design of our compound library employed the use of the chiral (R,R)-(-)-2,3-bis(t-butylmethylphosphino) quinoxaline and achiral 1,2-bis(diphenylphosphino)ethane (DPPE) phosphine ligands due to their biocompatibility and moderate lipophilicity when present coordination complexes or as ancillary ligands in organometallic compounds. For further investigation into the geometry and oxidation states of these complexes and their effects on anticancer activity, we explored the synthesis of dinucleur gold(I) complexes and (C^N)-cyclometalated backbone for the formation of an organometallic gold(III), all bearing phosphine ligands used in this study. We hypothesized that the small set of compounds with rich diversity including, ligand type, stereochemistry, oxidation state, geometry and distortion, would possess interesting biological properties for gold-based drug discovery.

### Synthesis and characterization

**Figure 1:**
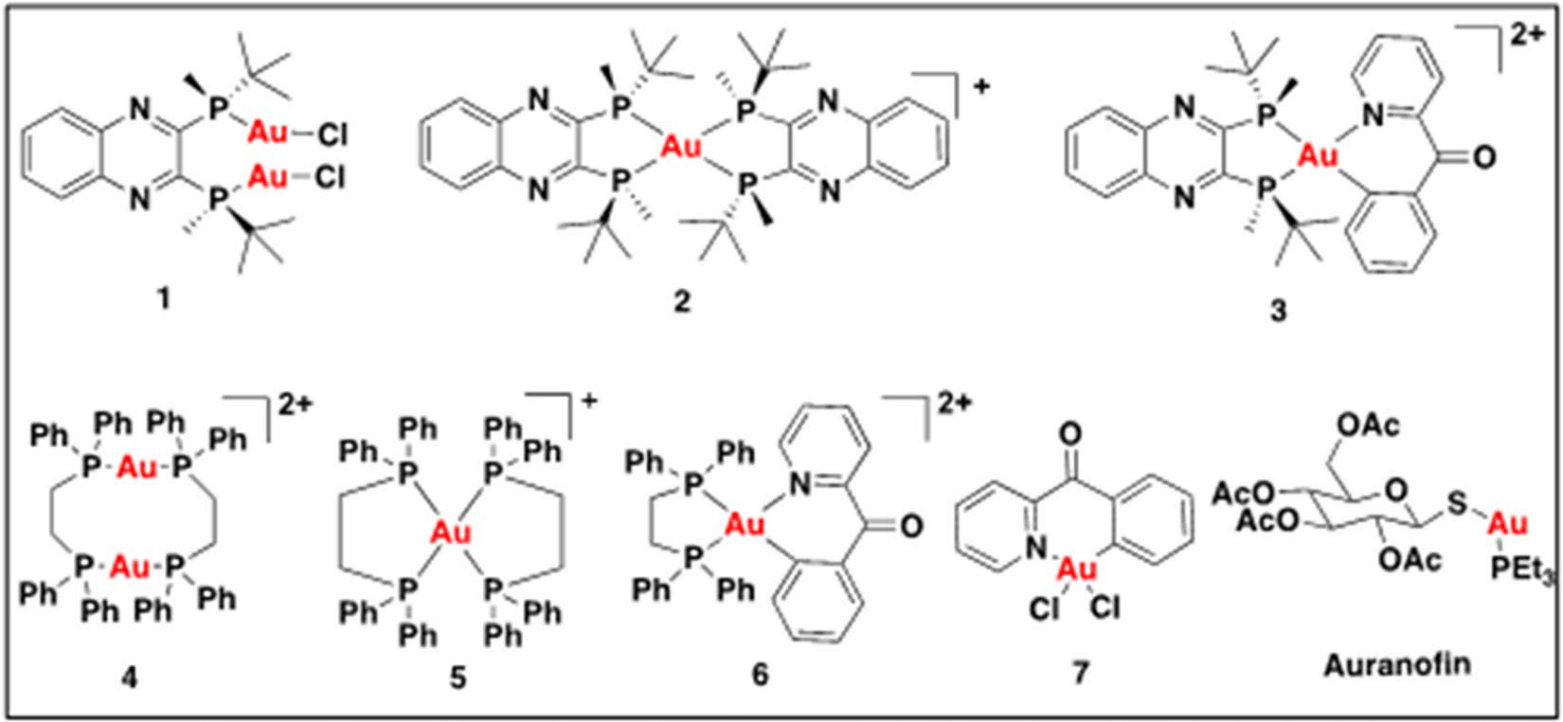
Chemical structures of gold complexes under investigation.

The gold-phosphine complexes under investigation in this study are depicted in Fig. 1. The chiral phosphine ligand, (R,R)-(-)-2,3-bis(t-butylmethylphosphino) quinoxaline^60^, which is well tolerated in mice was used for the synthesis of the respective Au(I) and Au(III) compounds, using tetrachloroauric acid trihydrate (HAuCl_4_·3H_2_O) or AuCl(tht), or (C,N)-cyclometalated Au(III)Cl_2_ starting reagents, demonstrated in compounds **1**, **2**, and **3**. To expand the diversity of gold-phosphine complexes, we synthesized compounds with the archetypical achiral phosphorus-donor ligand, DPPE, exemplified in **4**^61–63^, **5**^64–66^, according to previously reported protocols but with different anions, and the novel gold(III), **6**. The synthetic approach for compound **1**, was achieved by adding a chloroform solution of (R,R)-(-)-2,3-bis(t-butylmethylphosphino) quinoxaline to a cold solution of AuCl(tht) in chloroform. This complex was dried and recrystallized from chloroform/ether to give a pale yellow neutral compound. The synthesis of **2** was carried out by refluxing an equimolar concentration of HAuCl_4_·3H_2_O and (R,R)-(-)-2,3-bis(t-butylmethylphosphino) quinoxaline in chloroform for 1 h. After filtering through celite the reaction was purified by flash silica-gel chromatography to obtain **2** as a brick-red monocationic solid. We synthesized gold(III) analogs using the benzyolpyridine (C^N)-cyclometalated complex, **7**, which was synthesized as previously reported^67^. The chiral ((R,R)-(-)-2,3-bis(t-butylmethylphosphino) quinoxaline) and achiral (DPPE) bisphosphine ligands were used in a ligand substitution reaction with **7** to give **3** and **6** respectively, after purification by silica-gel column chromatography. Phosphines are strong coordinating ligands which can readily displace the chlorine atoms. In the reaction that leads to complex **3**, the formation of **2** in low yields is observed. This is as a result of reductive elimination promoted by the nucleophilic phosphine. It is a subject of intense investigation within our laboratory^68^. Complexes **4** and **5** bearing DPPE ligands were synthesized to investigate the effect of perchlorate counter anions on cellular activity. The complexes were purified by recrystallization from acetonitrile as white cationic solids.

The complexes were characterized by ^1^H, ^13^C, and ^31^P{^1^H} NMR spectroscopy(Fig. S1–23), UV spectroscopy, and high resolution electrospray ionization mass spectrometry. Purity of the complexes was confirmed by elemental analysis. The ^31^P{^1^H} of ((R,R)-(-)-2,3-bis(t-butylmethylphosphino) quinoxaline and DPPE have single resonances at −17 and −12 ppm respectively. Upon coordination with the gold center, the resonances shift downfield. For the gold(III) complexes, **3** and **6**, four or one signals in the ^31^P{^1^H} NMR were observed respectively. We attribute four signals in compound **3** to the presence of two diastereomers of the quinoxaline ligand and the one signals in compound **6**. P-coupling through a P-Au-P bridge was observed in both ^31^P{^1^H} NMR spectra. In compound **3**, *J*_pp_ was 0.12 ppm while in compound **6** it was 0.41 ppm. The presence or absence of the resonance structure of the P-C-C-P bridge influences *J*_pp_ coupling. Specifically, *J*_pp_ is smaller in the structure having the π electron bridge (complex **3**), and *J*_pp_ is relatively larger in the structure where the π bridge is nonexistent (complex **6**). The ^1^H-NMR and ^13^C{^1^H} NMR of compound **3** shows signals that can be attributed to diastereomer resonances. Furthermore, 2-dimensional COSY (Fig. S11) and HSQC (Fig. S12) was performed to assign the features of complex **3**. Overall, the compounds under investigation in this report were synthesized and unambiguously characterized by spectroscopy and their purity ascertained.

### Physical properties

**Figure 2:**
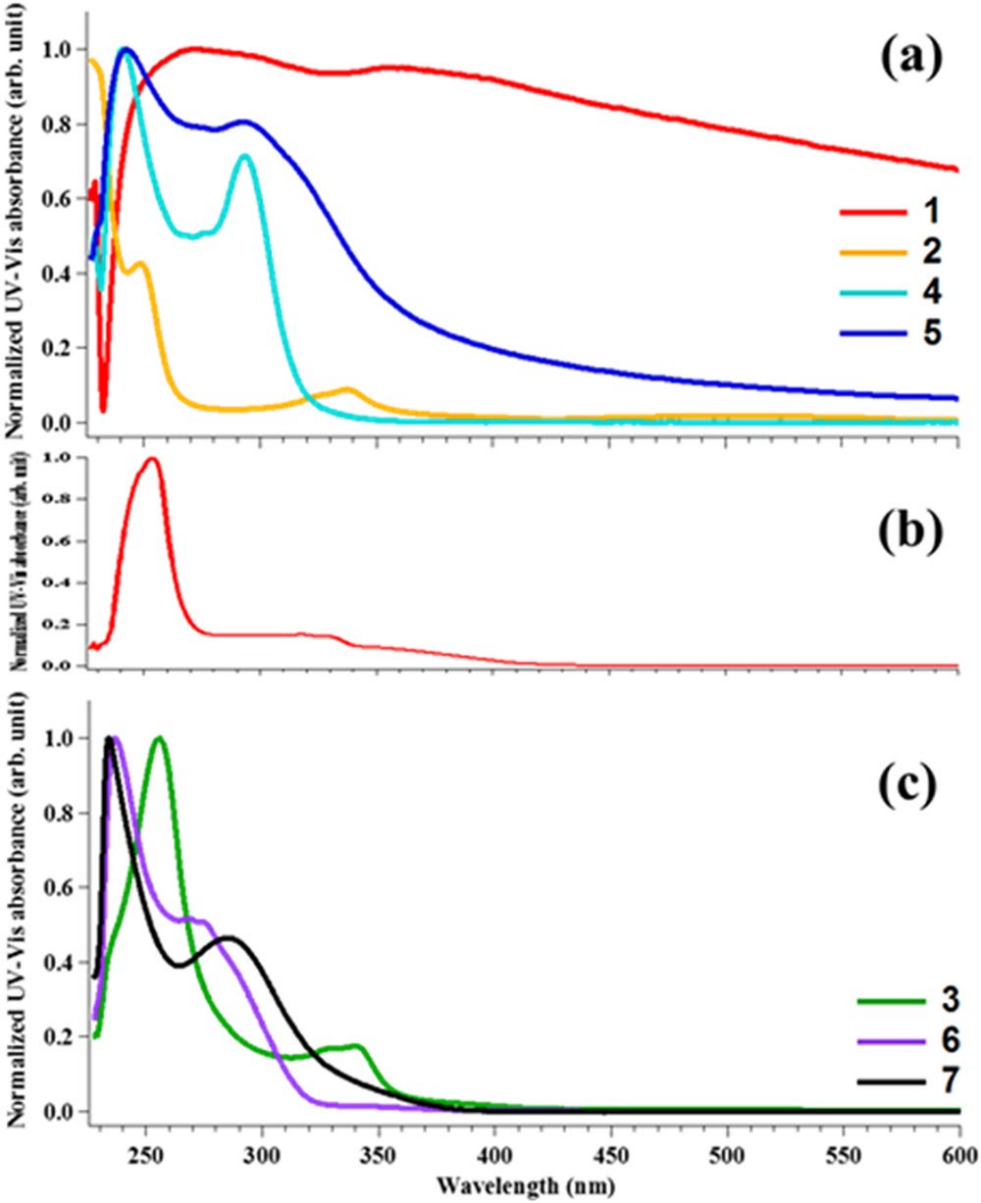
UV-Vis absorption spectrum of Au(I) series, **1**, **2**, **4**, and **5** in PBS (**a**), **1** in CHCl_3_ (**b**), Au(III) series, **3**, **6**, and **7** in PBS (**c**). Concentration of complexes = 50 μM. DMSO was used for stock solution. (The final concentration of DMSO was 1% of the total solution).

UV-Vis spectra of representative complexes were measured in air-equilibrated pH 7.4 phosphate-buffered saline (PBS) or DMEM cell culture medium, which constitutes several reducing amino acids in high concentrations (mM range). The spectra of the complexes bearing phosphine (**1**, **2**, **4**, and **5**) and both phosphine and cyclometalated ligands (**3**, **6**, and **7**) are shown in Figs 2 and S24–29. The absorbance profile of the complexes is significantly influenced by the nature of the phosphine ligands as well as the cyclometalated ligands.

The high-energy bands observed in the UV-vis spectra, likely correspond to π – π*transitions from the ligands^69,70^ or of ligand-to-metal charge transfer (LMCT) or metal-to-ligand charge-transfer (MLCT) character, which has been well-characterized by TDDFT calculations (*vide infra*). For example, in complex **7** there are two bands <300 nm, there is a high-energy band at 245 nm and another peak at 295 nm, which may be as a result of MLCT or LMCT transition^71–73^. There was no change in the absorbance profile of the gold(III) complex bearing cyclometalated and phosphine ligands (**3**) in PBS and minor changes in DMEM over the course of 48 h (Fig. S24–25). In contrast, the gold(I) bearing phosphine ligands experienced spectral changes in both PBS and DMEM over 48 h (Fig. S26–27). In PBS, the gold complexes show solubility and stability as shown from the absorption profiles in Fig. 2. A broad absorption band was noticeable for compound **1**, which can be attributed to possible aggregation due to the relatively planar and lipophilic character of the dinuclear complex, **1**, which can form π-stacking in aqueous solution. The representative examples offer insight into the role of cyclometalation and conjugated phosphine ligands to stabilize the gold center.

Complex **2**, representing Au(I), shows a continuous decrease in absorption peaks at 248 and 337 nm over time in PBS. In contrast, complex **3**, which is Au(III) showed no change in absorption bands. It can be deduced that the Au-P bonding in complex **2** is weakly stable in PBS compared to that of Au-P of complex **3**. This is supported by computational results, in that, while the four Au-P bonds of complex **2** are ~2.5 Å, the two Au-P of complex **3** show shorter bond lengths of 2.38 and 2.5 Å, respectively, indicative of stronger bonds. The stability associated with **3**, could be ascribed to both cyclometalated-Au bonding and adjacent Au-P bonds that enhance sigma-donation to the gold center. Complex **7**, which is another Au(III) display significant changes to the absorption bands in DMEM. It appears that chloride, a good leaving group, is substituted by amino acids contained in DMEM^74^. On the contrary, it shows stability in PBS, this seems like PBS does not contain components to replace Cl of complex **7**. Additionally, PBS contain 10 mM of NaCl that is likely to help stabilize the complex.

### Density functional theory and TD-DFT calculations

To gain theoretical insight into the gold complexes under investigation, DFT calculations were conducted. We used Gaussian 09 and the B3LYP functional was employed^75^. The basis set used was SDD on Au atom, taking into consideration relativistic effects and the Pople-type 6–31 G(d,p) was used for all other atoms. The geometry of the computed complexes was compared with the solved X-ray crystal structures to ensure the validity of the computations performed. The result is summarized in Fig. S31. Briefly, we considered the atomic groups around the gold atoms. For complex **1**, two independent distances between Au atom and P atom were 2.22 and 2.23 Å, and the calculated values showed minimal discrepancy, with an error of 3%. Similarly, two independent bond distances between Au and Cl was 2.28~2.29 Å, whereas the calculated values were within ~2% error. Interestingly, the bond angle of P-Au-Cl, from the crystal structure of **1** was 171° and the corresponding calculated value was 171°. In the case of **2**, the distorted structure of **2** had four P-Au bonds and its distances were all ~2.39 Å, and the calculated values were ~2.5 Å. Measured angles of three kinds of P-Au-P were 87.79°, 137.50°, and 106.48°, respectively, while the calculated values were 86.13°, 132.65°, and 111.58°. Unlike **1** and **2**, compounds **4** and **5** possess dppe ligands, which also showed minimal discrepancy between the experimental and calculated bond distances or angles. For complex **4**, the two gold metal atoms had two separate P-Au-P bonds, all four P-Au bond distances were 2.31 Å and the calculated values were ~2.38 Å. The X-ray structure revealed two P-Au-P angles as 166.8° and 177.8°, with the determined calculated angles were 169.1° and 171.0°, respectively. Complex **5** had a distorted structure like **2**. The distance between the four P-Au distances were all 2.40 Å, while the calculated values was in the range of 2.35~2.36 Å, showing less than 3% error. The three P-Au-P angles were 86°, 117°, and 118°, and the calculated values were 91°, 128°, and 114°, respectively. Taken together, this shows that DFT calculations and the method employed can be considered suitable for providing accurate geometric information of gold complexes.

To corroborate peak assignments within the experimental absorption profiles and further provide insight into HOMO-LUMO transitions, we performed TD-DFT calculations.

**Figure 3:**
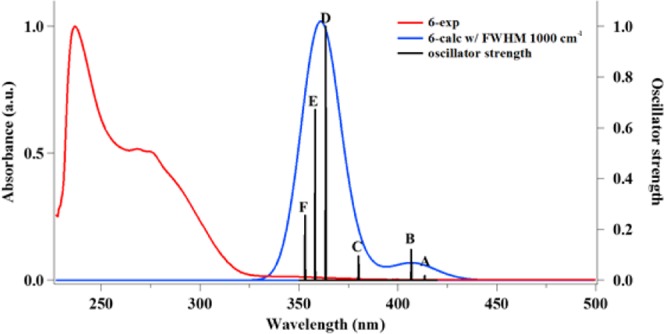
UV-Vis absorption spectrum of Au(III) complex, **6**, in PBS at the concentration of 50 μM. DMSO was used for stock solution (red) and theoretical spectrum (blue) and oscillator strengths, *f*, from Table 1 for **6** (black).

**Figure 4:**
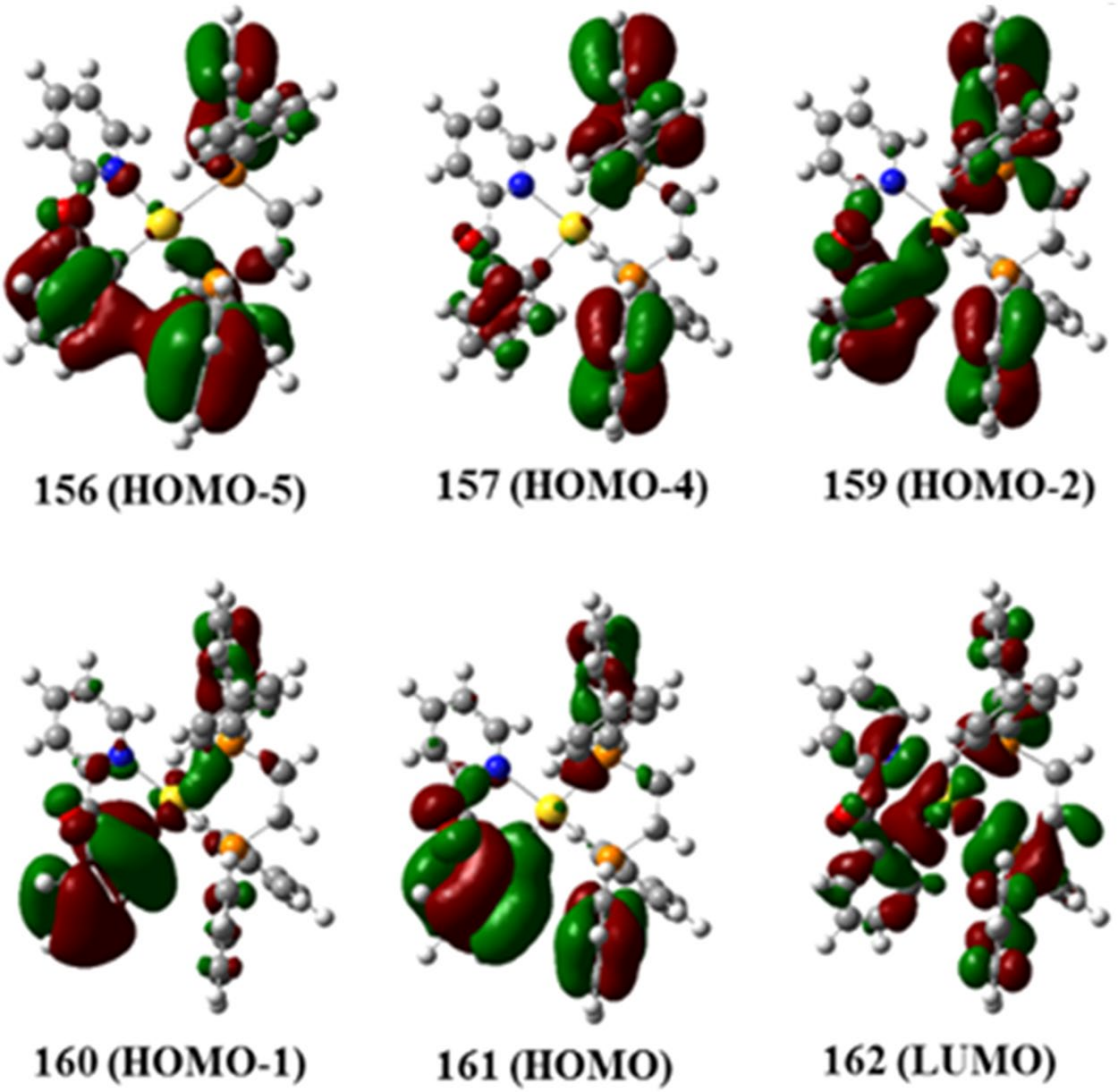
LUMO, HOMO, HOMO-1, HOMO-2, HOMO-4, and HOMO-5 electronic density maps for **6**. Calculated on B3LYP, SDD on Au and 6–31 G(d,p) on others.

In Fig. 3, the experimental UV-Vis spectrum for complex **6** was compared to the calculated spectrum. The oscillator strength and molecular orbital (MO) contributions obtained from the theoretical calculation is summarized in Table 1. The limitation of the method and basis sets used in the theoretical calculation as a result of the large relativistic effect of gold present some discrepancy, which is consistent with other gold systems in the literature. In the UV-Vis spectra, generally, 2–3 peaks were observed and the theoretical calculations (TD-DFT) were carried out to establish the relationship between the observed peaks and the associated electronic transition. The experimental absorption profile show a high-energy absorption peak located at approximately, 250 nm and a red-shifted shoulder band at ~275 nm. However, the calculated spectra show a high energy band at 350 nm and its related red-shifted band at 400 nm, confirming a discrepancy of about 100–150 nm from the experimental spectra. This is typical for gold systems^76,77^. Furthermore, for complex **1**, the contribution of HOMO-2 to LUMO was the largest, corresponding to a MLCT transition. The HOMO-2 is largely localized on the gold and the neighboring Cl atoms and the LUMO on the phenyl/pyrazine of quinoxaline. A similar charge transfer is seen in HOMO-LUMO transition for compound **1** (Fig. S32). Additionally, MLCT is also seen in complexes **2** (Fig. S33) and **5** (Fig. S34). **2** has the same ligand as **1** and shows similar metastases: λ = 221 nm (HOMO to LUMO + 2) and λ = 249 nm (HOMO-1 to LUMO + 1), which are charge transfer from the gold center to both quinoxaline ligands. For λ = 326 nm, the HOMO-LUMO transition represents a similar transfer from the gold center to the periphery of the molecule. For complex **5**, electrons distributed around the metal migrate to neighboring phenyl groups: λ = 243 nm (HOMO to LUMO + 3) and λ = 292 nm (HOMO to LUMO). Similarly, for complex **5**, the high energy is caused by MLCT of HOMO to LUMO + 3, and the neighboring λ = 292 nm (HOMO to LUMO) by gold center to the surrounding phenyl groups of the the dppe ligand. In contrast, complexes **3**, **4**, **6** and **7** show mainly LMCT transitions. For **3**, high energy peaks (λ = 256 nm) were observed in quinoxaline-to-gold charge transfer (HOMO-3 to LUMO and HOMO to LUMO + 1) and low energy peaks were considered as transition from benzopyridine to gold center (HOMO-2 to LUMO and HOMO-1 to LUMO, Fig. S35). Complex **4** is largely due to the transition of HOMO-2 to LUMO for λ = 241 nm, from the two phenyl rings of the dppe ligand to the gold, and HOMO to LUMO for λ = 294 nm from the phenyl to gold (Fig. S36). For **6**, λ = 237 nm correspond to, HOMO-2 to LUMO, which is consistent with a LMCT transition based on the localization of the HOMO-2 and the LUMO and λ = 268 nm is as a result of a similar transition, HOMO to LUMO (Fig. 4). Complex **7** also possess a similar profile with λ = 234 nm corresponding to HOMO-2 to LUMO + 1, which is a LMCT transition, and λ = 285 nm, resulting from HOMO to LUMO with LLCT transition (Fig. S37). Although the complexes use similar ligands (quinoxaline, benzoylpyridine, and dppe), the type of charge transfer in the UV-Vis spectrum is different (MLCT, LMCT, or LLCT)^69–73^ because the different types of ligands, combination of ligands, and the different coordination number of the gold make a difference. The Au(III) complexes all display LMCT transitions and the Au(I) complexes display MLCT transition with the exception of **4**. Overall, the study provide insight into the MO contributions of the experimentally obtained absorption profiles of these cytotoxic compounds.

**Table 1:** TD-DFT Excitation Calculations for **6**. ^a^Value is 2 × (coeff)^2^ × 100.

Excitation	λ_exp_ (nm)	λ_calc_ (nm)	Osillator strength	MO#, (Contributions^a^)	
A		414	0.0015	160 → 162 (68)	
				161 → 162 (28)	
B	268	407	0.0096	156 → 162 (2)	LMCT
				159 → 162 (16)	
				160 → 162 (21)	
				161 → 162 (58)	
C		380	0.0066	152 → 162 (49)	
				152 → 163 (20)	
				153 → 162 (2)	
				153 → 163 (11)	
				158 → 163 (3)	
				159 → 162 (5)	
				159 → 163 (3)	
				160 → 163 (9)	
				161 → 163 (35)	
D	237	364	0.1100	157 → 162 (49)	LMCT
				159 → 162 (73)	
				160 → 162 (7)	
				161 → 162 (6)	
E		358	0.0424	156 → 162 (49)	
				157 → 162 (24)	
				158 → 162 (56)	
				161 → 162 (4)	
F		353	0.0301	156 → 162 (5)	
				157 → 162 (50)	
				158 → 162 (39)

### Variable temperature NMR

Prior to biological investigation of these complexes, we assessed the thermal stability of representative compounds in DMSO-*d*_6_ and D_2_O. Given that stock solutions of the complexes were prepared in DMSO prior to biological, photophysical, or electrochemical evaluation DMSO-*d*_*6*_ was used. Consequently, all the biological evaluation is in aqueous base medium, thus studying their stability in D_2_O was appropriate. We measured the ^1^H-NMR of complexes **1–6** within a temperature range of 24–80 °C (Fig. S38–50). There were no obvious changes in the ^1^H-NMR spectra for the respective complexes studied over the temperature range, indicative of stability of these complexes under harsh conditions. In summary, these gold compounds show thermal stability in DMSO and D_2_O, which is an important characteristic for biologically relevant transition metal complexes.

### X-ray crystallography

**Figure 5:**
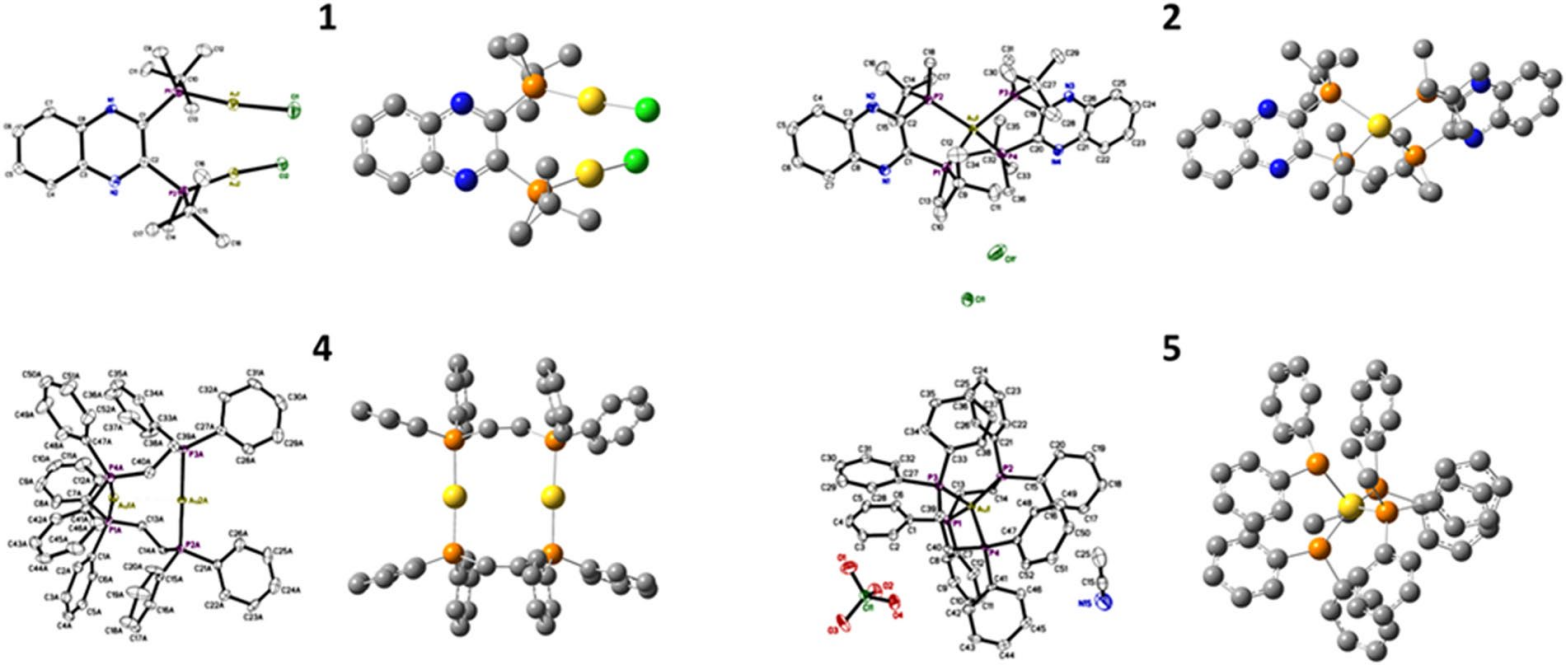
X-ray crystal and calculated structures of **1**, **2**, **4**, and **5**. Ellipsoids are drawn at 50% probability level. Hydrogen atoms bound to carbon atoms and solvent are omitted for clarity. The structures were calculated on B3LYP/SDD, 6–31 G(d,p). No imaginary frequencies were found.

Single crystals of four complexes out of the six compounds studied were obtained and the crystal structures were determined by X-ray crystallography. Crystal structures with optimized structures for **1**, **2**, **4**, and **5** are shown in Fig. 5. We note that **4**^61–63^, and **5**^64–66^ share cationic similarity to structures previously reported, for different salts of these cationic complexes. A comparison of the previously reported structures and ours reveal the perchlorate anions and no significant differences in the overall geometry of the gold complex. Moreover, the dinuclear gold compound, **4** crystallizes in the triclinic P1 space group, while **5** crystallizes in the orthorhombic space group, Pca2_1_. Crystallographic information and selected interatomic distances for compounds **1**, **2**, **4**, and **5** can be found in Table S1–4. For the dinuclear complex, **1**, it crystallizes in the orthorhombic space group, *P*2_1_2_1_2_1_. There is a slight distortion in the linear geometry of the P-Au-Cl bonds, for example, the P1-Au1-Cl1 angle is 171.77(9). The rigid chiral bisphosphine ligand may be the culprit for the observed distortion. Additionally, the bond angles C-P-Au were different for the methyl and tert-butyl group bonded to the phosphorus. For example, C9-P1-Au1 is 108.1(3), whereas C10-P1-Au1 is 112.6(3), indicative of a larger angle influenced by the bulkier tert-butyl fragment. Importantly, the dinuclear gold compound does not have a bond between the two gold atoms. Compound **2** crystallizes in the monoclinic, C2 space group which is a deviation from **1**, despite the similar chiral ligand environment. The complex exhibits an unusually distorted square planar geometry for Au(I) species. The Au-P bond distances for both **1** and **2** are slightly invariant as we observe a longer bond distance for Au-P in **2**, when compared to Au-P distances in **1**. For example, complex 1, Au1–P1: 2.225(2) Å and Au2–P2: 2.236(2) Å, and for the complex 2, Au1-P1: 2.3889(14) Å, Au1-P4: 2.390(2) Å, Au1-P2: 2.3928(19) Å, and Au1-P3: 2.3964(15) Å, respectively. The distorted square planar geometry of **2**, contribute to the longer Au-P bond distances than that of **1**. We note that the Au-P bonds for the structure of **4** is comparable to that of compound **2** but the Au-P bond distances for **5** are longer, averaging 2.406(8) Å. This is consistent with the fact that distorted gold(I) complexes such as **2** and **5** exhibit slightly longer Au-P distances than their dinuclear counterparts, **1** and **4**. The cone angle of DPPE to the Au center in **5** are smaller (P1-Au1-P2 85.97(3) Å) in comparison to the ((R,R)-(-)-2,3-bis(t-butylmethylphosphino) quinoxaline ligand in **2** (P1-Au1-P2 87.91(5) Å).

### Electrochemistry

**Figure 6:**
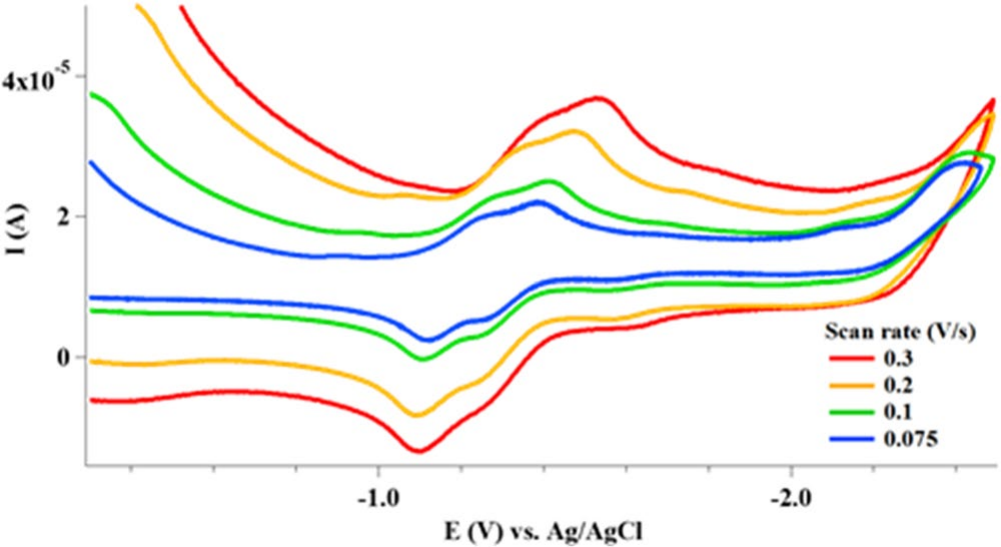
Cyclic voltammograms recorded at a platinum electrode in DMSO solution of 1.0 mM **2**, with NaClO_4_ supporting electrolyte; scan rate 0.3, 0.2, 0.1, and 0.075 V sec^−1^.

To elucidate the reduction and oxidation properties of these gold complexes, we measured the cyclic voltammetry in DMSO with sodium perchlorate (NaClO_4_) as supporting electrolyte at a Pt electrode, using CH-600D potentiostat equipment. One reversible reduction event was observed for the gold(I) complex, **1** at a potential of −1.20 V (Fig. S52). In an oxidative sweep, an oxidation peak appears at −0.98 V. This phenomenon is observed in the free ligand, ((R,R)-(-)−2,3-bis(t-butylmethylphosphino) quinoxaline (QuinoxP), which possess a more negative reduction potential at −1.68 V. Complex **2**, displays two successive reversible processes with reduction potentials at −1.26 V and −1.38 V respectively (Fig. 6). Note that **2** is supported by two QuinoxP ligands with distortions from a classical linear Au(I) complex that may lead to two closely distinct redox events. The result and the absence of metallic gold at the platinum working electrode led to the conclusion that the overall reduction for this class of Au(I) complexes is ligand-centered rather than metal-centered^78^.

The electrochemical behavior of **4** and **5**, which are Au(I) complexes bearing DPPE ligands were investigated. Complex **4** shows irreversible reduction peak at −0.84 and −1.54 V (Fig. S53). Similarly, **5** show irreversible reduction events at −0.81 V and −1.55 V with no oxidation events (Fig. S54). The events for **4** and **5** are considered to occur on the ligand, given that the reduction potentials of compounds **4** and **5** were similar and consistent with dppe as well as the lack of gold precipitation. We attribute the observation to stability of the complexes and ligand-centered redox events. Furthermore, we studied the electrochemical behavior of the gold(III) complexes, **3** and **6** in DMSO using cyclic voltammetry. As shown in Fig. S55, irreversible reduction peak at −0.80 V was observed and a reduction peak at −1.46 V for **3**. In addition, a corresponding oxidation event was observed at −1.27. **6** showed two reversible reductions at −1.01 and −1.37 V, and the paired oxidation events at –0.86 and −1.31 V were observed, respectively (Fig. S56). Typically, gold(III) undergo two separated Au(III)/Au(I) and Au(I)/Au(0) steps in non-aqueous environment or three electron^79^, Au(III)/Au(0) reduction in aqueous solutions due to disproportionation of Au(I) in aqueous solution^80–82^. Our observation lean more towards the former with an initial Au(III)/Au(I) reduction for **3** and **6**.

### Reactivity with BSA

**Figure 7:**
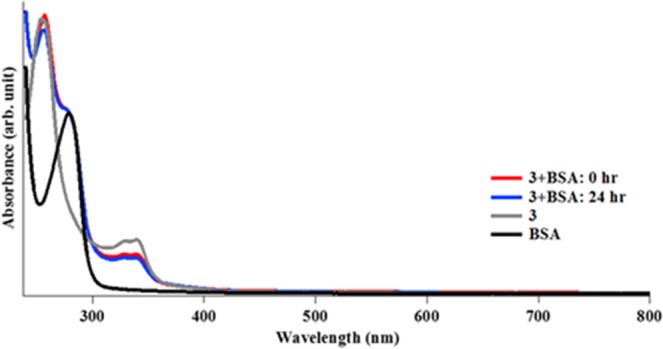
UV-Vis absorption spectra of **3**, BSA, and **3** and BSA mixture in PBS. Concentration of complex **3** and BSA = 25 μM. DMSO was used for stock solution of **3**.

Spectrophotometric investigations of the gold complexes (**1**–**6**) described in this report in a reaction with bovine serum albumin (BSA) was performed under physiological conditions. Taking advantage of the intense absorption bands of the gold complexes and BSA, we monitored the progress of the reaction using 1:1 ratio of BSA and buffered solutions of each gold complex over 24 h. Serum albumin is a major soluble protein component present in the circulatory system and has many physiological functions^83^. Importantly, BSA acts as a carrier for various pharmacological agents. It must be noted that BSA has been extensively studied, and shares homology with human serum albumin (HSA)^84^. Often, gold compounds bind methionine and cysteine residues in BSA via the sulfur atoms. The inherent absorption peaks for complexes **2**–**6** were minimally affected by the addition of the BSA solution over a 24 h period (Figs 7 and S57–S61). It is also worth pointing out that the peak corresponding to the absorption of BSA at 280 nm was unaffected under the experimental conditions. The ability for compound **1** to aggregate in aqueous solution limited the ability to evaluate it under the experimental conditions. However, a solution of compound **1** with BSA did not affect the peak attributed to BSA (Fig. S62). For compound **2**, while a decrease in the absorption band corresponding to MLCT at ~250 nm was observed, no changes in the band at 325 nm was observed in the course of the experiment. The observed decrease is consistent with the time-dependent study of **2** in PBS. Also, the peak corresponding to BSA remained unchanged throughout the 24 h period. Complexes **3** and **6** are gold(III) compounds with cyclometalated ligands but different bisphosphine ligands. Interestingly, none of the peaks associated with the complexes or BSA changed, indicative of stability in the presence of BSA over 24 h. While the shoulder peak at 300 nm disappeared in the case of complex **4** (LMCT), the BSA peak at 250 nm was unmodified. Complex **5** on the other hand did not display any alteration in its peak. In general, there was no indication of the formation of metallic gold as no brown precipitate formed in any of the reaction over the duration of the experiments. Using HPLC (Fig. S63–S64) we characterized the extent of interaction of the test compounds and BSA. This approach can be used to quantify potential binding of gold compounds with BSA by evaluating the retention times and area of peaks associated with individual agents as well as reaction solutions of test compounds and BSA. Following the UV-vis studies, we used compounds **2** and **3** for the HPLC study based on the common chiral ligands but different oxidation states. There were no changes in the peaks, indicative of no covalent modification of BSA or changes to the gold compounds. These compounds by virtue of their coordinated ligands and cyclometalation demonstrate high stability even towards proteins like BSA. Detailed studies by Minghetti and co-workers^85^ on the reactivity of selected gold(III) complexes with serum albumin under similar experimental conditions showed varied stability of the gold complexes in the presence of BSA. This result has important implications for the pharmacological activity of these gold complexes, in that they can avoid premature deactivation until they reach their target and also reduce off-target effects.

### Cellular toxicity studies

The antiproliferative properties of these gold complexes were evaluated in a panel of cancerous cell lines using crystal violet assay for H460 and OVCAR8. We used an ATP-dependent luminescence cell assay, cell titre glo, for K562 cells. To extend the therapeutic utility of these novel drug candidates, we performed cytotoxicity studies with normal retinal pigment epithelium, RPE-NEO. Auranofin and cisplatin were used as controls. We obtained dose-response curves from the cell viability experiments and subsequently derived IC_50_ values (concentration required to kill 50% of cells) summarized in Table 2. Complexes **1**–**6** displayed high nanomolar to low micromolar cell killing which are 2–10 folds better than cisplatin. None of the gold-phosphine complexes display cross-resistance evidenced by indifferent toxicities in cisplatin resistant cells, including the well-characterized high grade-serous ovarian cancer (HGSOC) cell line, OVCAR8, which demonstrate the example of high potency of these novel Au complexes in clinically relevant tumor cells. Generally, the gold compounds studied are slightly less potent toward RPE-Neo cells, indicative of selective toxicity for cancerous cells compared to healthy cells. Compounds **2** display ~8–40-fold higher potency in cancer cells than the normal RPE-Neo cell line.

Table 3 summarizes the electrochemical potentials, LUMO eigenvalues and representative cytotoxicity of the gold complexes studied in this report. In general, the redox active behavior of the complexes may be suggestive of redox induced cell-death. However, we could not establish a positive correlation between reduction potentials, LUMO eigenvalues and cytotoxicity. Several factors affect the induction of cell death by metal-based drugs and such correlations must be cautiously applied.

**Table 2:** IC_50_ Values (µM) of **1–6**, cisplatin, and auranofin against a panel of cancer cell lines after 72 h exposure. Cisplatin stock was prepared in PBS and gold compounds were freshly prepared in DMSO and used immediately. DMSO concentration was <1%.

Compound	IC_50_(μM) K562 (leukemia)	IC_50_ (μM) H460 (lung)	IC_50_ (μM) OVCAR8 (ovarian)	IC_50_ (μM) RPE-NEO (normal)
1	0.92 ± 0.02	0.43 ± 0.04	0.10 ± 0.01	0.47 ± 0.01
2	0.35 ± 0.05	0.16 ± 0.02	0.83 ± 0.12	6.49 ± 0.02
3	1.74 ± 0.18	0.46 ± 0.02	0.38 ± 0.04	0.55 ± 0.02
4	0.23 ± 0.08	0.16 ± 0.03	0.79 ± 0.13	1.53 ± 0.01
5	0.45 ± 0.03	2.53 ± 0.22	0.18 ± 0.06	2.94 ± 0.01
6	1.65 ± 0.12	0.22 ± 0.01	1.09 ± 0.34	1.11 ± 0.01
auranofin	0.42 ± 0.05	0.52 ± 0.02	0.34 ± 0.06	1.09 ± 0.03
cisplatin	13.05 ± 1.02	5.63 ± 1.02	8.95 ± 0.44	12.98 ± 0.04

**Table 3:** Electrochemical potentials, LUMO/HOMO energies, and cytotoxicity of the gold complex **1**–**6**.

Complex	Redox potential (eV)		Lumo (eV)	Homo (eV)	IC_50_ (μM)
	Epc	Epa			K562
1	−1.2	−0.979	−3.18	−6.07	0.92 ± 0.02
2	−1.26, −1.38	−1.12, −1.26	−2.58	−4.24	0.35 ± 0.05
3	−0.80, −1.46	−1.27	−8.52	−12.03	1.74 ± 0.18
4	−0.84, −1.54		−6.11	−10.90	0.23 ± 0.08
5	−0.81, −1.55		−3.10	−7.76	0.45 ± 0.03
6	−0.01, 1.37	−0.86, 1.31	−8.13	−11.90	1.65 ± 0.12

### Cellular uptake studies

**Figure 8:**
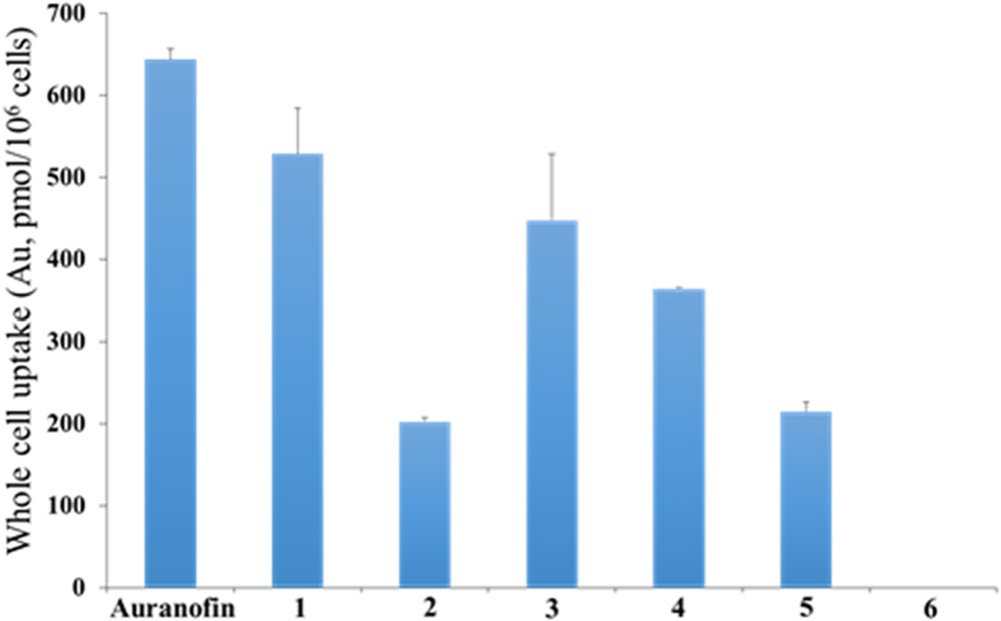
Whole cell (OVCAR8) uptake results from auranofin (5 μM) and complexes **1**–**6** (5 μM). Cells were incubated with compounds for 15 h.

To gain insight into the intracellular behavior of the gold reagents, we studied the whole cell uptake of **1–6** and auranofin as well as subcellular distribution of Au for **1** and **3**. OVCAR8 cells were incubated with the gold compounds (5 μM) for 15 h. The Au content was measured by using ICP-OES, calibrated with HAuCl_4_ standard. Differential whole cell uptake was observed for all compounds with neutral complexes, auranofin and **1** displaying the highest uptake respectively and **6** taken up the least (Fig. 8). Generally, increased cellular uptake correlates well with high cytotoxicity and biological effects thereof. Previous work using other gold phosphine complexes show that increased uptake is accompanied by cell growth inhibition, and that a parabolic dependence between cell growth inhibition, uptake, and lipophilicity does exist^86,87^. Thus, it is possible to predict whether changes in the oxidation number of gold complexes and changes in lipophilicity of the complexes would have an effect on uptake and growth inhibition. However, the set of test compounds investigated in this report did not establish significant correlations of uptake and cytotoxicity. A clear observation of the data revealed higher uptake for dinuclear complexes (**1** or **4**) over mononuclear gold complexes bearing chiral or achiral phosphine ligands respectively. It is likely that the dinuclear gold increases lipophilicity of the agents. Additionally, the use of chiral QuinoxP ligands have a higher lipophilic character over the dppe ligands. We evaluated **1** and **3**, which bear the same chiral ligand but different oxidation states in a series of biological experiments and although IC_50_ values do not show significant differences, apoptosis assay (vide infra) reveal a much higher early to late-stage apoptosis for **1**, which has a higher cellular uptake than **3**. Following the incubation of OVCAR8 cells with **1** or **3**, we isolated the nuclear component from the cytoplasm and the residual cell pellet. As shown in Fig. S65, complex **1** or **3** was localized predominantly to the cytoplasmic fraction at 200 pmol/10^6^ cells concentration. In contrast, a lower localization within the nuclear fraction at 15 pmol/10^6^ cells was observed for both **1** and **3**. The similar Au concentration in the subcellular fractions despite the different structural scaffolds may be the clue to the similar growth inhibition observed. The concentration found within the cell residue (pellet) was similar to that of the nuclear fraction, within margin of error. These results demonstrate that the new Au constructs largely localize and may target cytoplasmic proteins owing to their lipophilic chiral ligands with minimal potential to target genomic DNA.

### Mode of action studies

**Figure 9:**
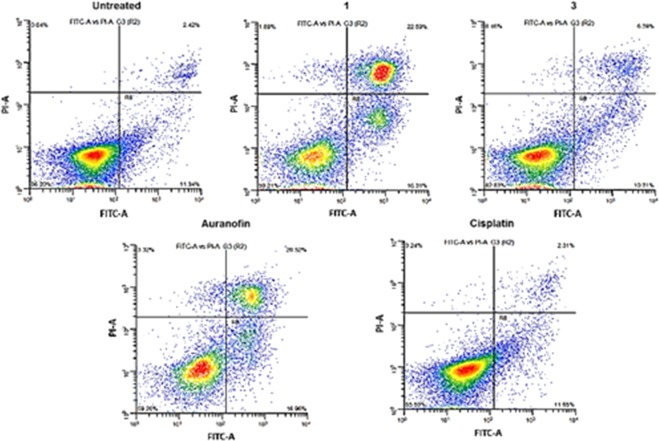
FITC Annexin V/PI apoptosis dead cell assay. OVCAR8 cells were used. Plots of untreated cells (negative control), cells treated with **1** (2 μM for 48 h), **3** (2 μM for 48 h), auranofin (2 μM for 48 h), or cisplatin (2 μM for 48 h).

To further understand the cellular responses evoked by **1** or **3**, the compounds were examined for apoptotic effects. Mitochondria dysfunction and ER stress can result in apoptosis. Characteristically, cells undergoing apoptosis have associated cell membrane disorientation. This leads to efficient phosphatidylserine residue translocation from the interior of the cell to the exterior membrane, which can be readily detected by Annexin V molecules. We performed a dual-stain experiment involving FITC-labeled Annexin V and propidium iodide (PI) for flow cytometry analysis. The HGSOC cell line, OVCAR8, were treated with **1**, **3**, auranofin or cisplatin for 72 h. We observed a large population occurring as late-stage apoptotic cells for the Au(I) compounds, i.e. **1** and auranofin. In general, all the Au complexes induce significant apoptosis over cisplatin as shown in Fig. 9. Taken together, these Au reagents display enhanced *in vitro* potency when compared with the conventional platinum(II)-based agent, cisplatin.

**Figure 10:**
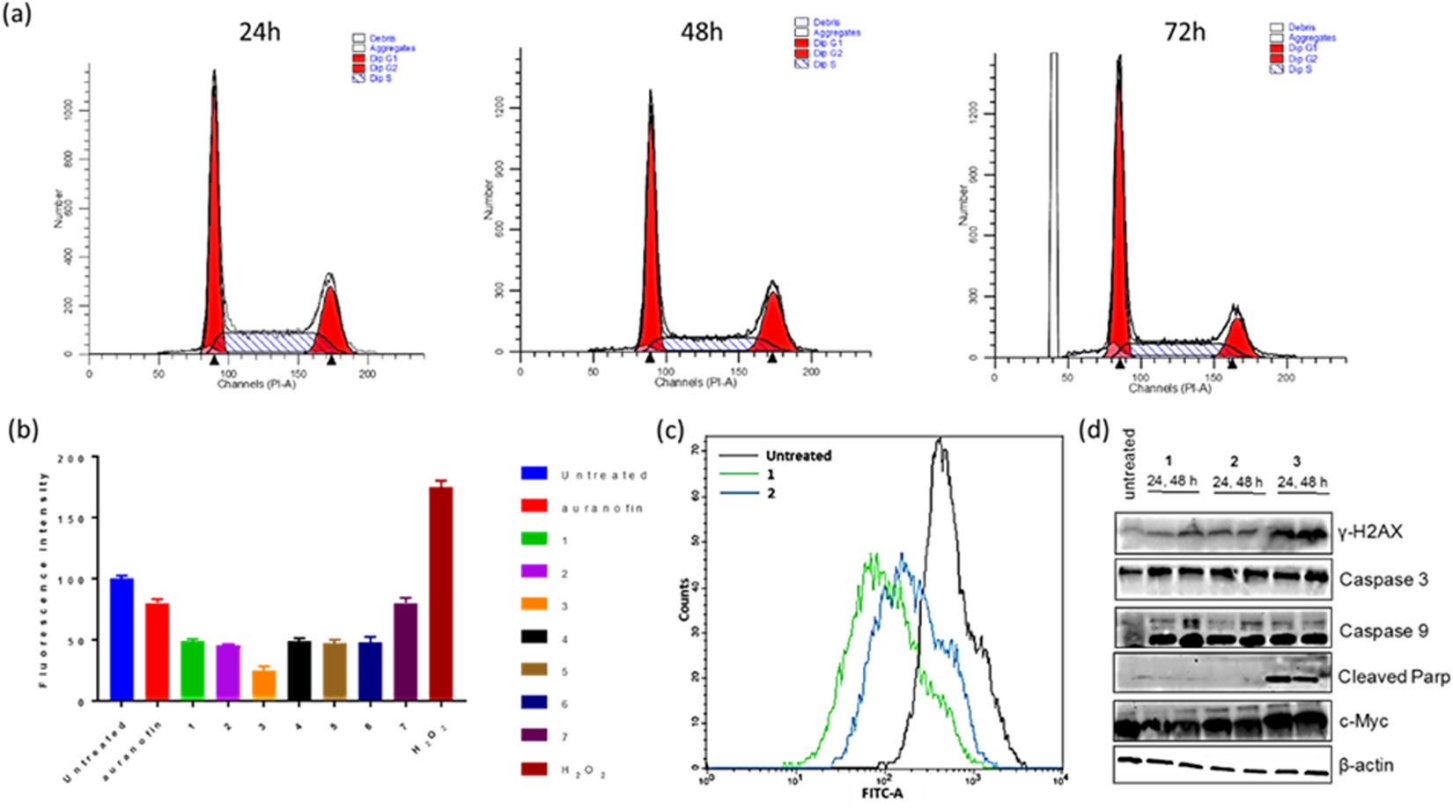
(**a**) Histogram representing the different phases of the cell cycle of OVCAR8 in the presence or absence **1** (2 µM) over the course of 72 h. 24 h treated with **1**: G1: 42.22%, S: 36.39%, G2/M: 21.38%, 48 h treated with **1**: G1: 47.27%, S: 34.46%, G2/M: 18.26%, 72 h treated with **1**: G1: 54.99%, S: 29.48%, G2/M: 15.53%. (**b**) Intracellular ROS using OVCAR8 cells and dihydrorhodamine 123 as dye. Plots show untreated cells (negative control), H_2_O_2_ treated cells (positive control), and cells treated with **1–7** (5 μM, overnight) or auranofin (5 μM, overnight). (**c**) Histogram plot of the effects of **1** and **2** (µM) on mitochondrial membrane potential using rhodamine 123 in OVCAR8 cells. (**d**) immunoblotting for the expression of apoptosis and DNA damage response proteins in OVCAR8 cells following incubation with **1**, **2**, or **3**. (full-length blots/gels are presented in Supplementary Fig. 68).

Suspicious of mitochondria dysfunction and ROS production as primary inducers of cell death, we conducted intracellular reactive oxygen species (ROS) experiments using human cancer cell line, OVCAR8 (Fig. 10b). Mitochondria are the power hub that control cell death signaling within cells and generate large amounts of ROS within the cell. That said, the mitochondria is also susceptible to ROS attack. Thus, measuring ROS production induced by the gold compounds presented in this report is rational. Under the experimental condition, compound treated cells revealed a low amount of ROS. It is fair to say that all compounds inhibit ROS, especially the Au(III) reagent, **3**, as measured by the fluorescence of dihydrorhodamine 123 using flow cytometry. Together, the study suggests the ability of these gold complexes to modulate intracellular ROS.

Having established the effects of **1**–**6** on intracellular ROS, mitochondrial membrane potential studies were conducted to monitor mitochondrial insults. Notably, **1** and **2**, largely depolarized the mitochondria membrane by measuring the fluorescence of rhodamine 123 using flow cytometry (Figs 10c, S66). Well studied organic small-molecule that modulate ROS are known to induce mitochondrial membrane potential depolarization resulting in cell death^88^. Additionally, a few organometallic gold complexes induce mitochondria depolarization as a result of mitochondrial dysfunction or ER stress^1,89^. Mitochondria membrane depolarization often stimulates caspase signaling, hence, using immunoblotting analyses, we monitored changes in the expression of biomarkers related to apoptosis and DNA damage response (Fig. 10d). OVCAR8 cells incubated with **1**–**3** for 48 h revealed marked increase in caspase 3 and 9 levels^2,41,42,90^. While **3** induced differential expression of cleaved PARP and γ-H2AX, compounds **1** and **2** did not, suggesting that **3** may impose a broader mechanism of action. The generation of ROS is capable of compromising mitochondria function, leading to the activation of caspase-3 and cleavage of PARP. Our experimental results parallel the existing mode of action with a unique differentiation. For example, complexes **1** and **2** are not involved in ROS production and do not induce PARP cleavage. This suggests that within the apoptotic program, **1** and **2** may cause late apoptosis as rightly observed for **1** (Fig. 9) since PARP cleavage occurs early during apoptosis in order to avoid energy (NAD and ATP) depletion needed for later stages in the apoptotic program^91,92^. Overall, these studies confirm the differentiated mechanism of novel chiral or non-chiral gold complexes that are capable of inhibiting ROS in cells, setting off mitochondrial membrane potential depolarization as a result of perturbed redox homeostasis that lead to apoptosis in cancer cells. The preliminary mechanistic activity of the gold candidates was characterized in OVCAR8 cells by assessing cell cycle effects. Using flow cytometric analyses on OVCAR8 cells after treatment with **1** or **3**, incubating the cells with each agent at ~two-fold higher concentrations than their respective *in vitro* IC50 values for either 24, 48 or 72 h. The study revealed that **1** and **3** stalled the cell cycle at the G0/G1 phase in a time-dependent manner. Notably, within 24 h, there was an increase in the G2/M phases, which diminished to levels relative to untreated controls by 72 h (Figs 10a and S67). This observation differs from cell cycle patterns of cisplatin-treated cells, which primarily induce G2/M phase arrest by 72 h for most ovarian cancer cells. Undoubtedly, a more rigorous biological characterization is required to fully elucidate the mechanism.

## Conclusion

The synthesis and characterization, electrochemical, and biological evaluation of six gold complexes bearing chiral and achiral bisphosphine ligands were investigated. All compounds display potent cytotoxic effects in cancer cells over normal cells. Particularly, the gold compounds described show high antiproliferative activity in a panel of cell lines including the clinically relevant HGSOC OVCAR8 cell line, ranging from 2–10-fold lower IC_50_ values when compared to cisplatin. The complexes investigated offer a broader mechanism of action than cisplatin or auranofin including apoptosis, mitochondria depolarization and G0/G1 cell cycle arrest. Despite differential intracellular accumulation of complexes **1**–**6**, the cytotoxic effects were not significantly invariant. The redox potential of these complexes is affected by the choice of ligands. Additionally, theoretical insight using DFT and TD-DFT calculations demonstrated HOMO-LUMO orbital distributions which can inform future design and donor effect of ligands for stability and prediction of excited states. Although this study uses a relatively modest set of compounds, they represent an important proof-of-concept study for the development of new gold-phosphine complexes that possess ligand variability and different oxidation states. Ongoing studies are being performed to expand the scope of stable gold(III)-phosphine complexes as effective anticancer agents.

## Methods

### General information

Auranofin and Cisplatin were purchased from Santa Cruz Biotechnology Inc. and Strem, respectively and used without further purification. All other reagents were purchased from Strem, Sigma Aldrich, or Alfa Aesar and used without further purification. All reactions were carried out under normal atmospheric conditions. Deuterated solvents were purchased from Cambridge Isotope Laboratories (Andover, MA). ^1^H, ^13^C, ^31^P, COSY, and HSQC NMR spectra were recorded on a Varian Unity 400/500 NMR spectrometer with a Spectro Spin superconducting magnet in the University of Kentucky NMR facility. Chemical shifts in ^1^H, ^13^C, COSY, and HSQC NMR spectra were internally referenced to solvent signals (^1^H NMR: DMSO at δ = 2.50 ppm; ^13^C NMR: DMSO at δ = 39.52 ppm), and those in ^31^P NMR spectra were externally referenced to 85% H_3_PO_4_ in D_2_O (δ = 0 ppm). Electrospray ionization mass spectrometry (ESI-MS) was performed on an Agilent Technologies 1100 series liquid chromatography/MS instrument. High-resolution mass spectra (HRMS) were obtained by direct flow injection (injection volume = 5 or 2 μL) ElectroSpray Ionization (ESI) on a Waters Qtof API US instrument in the positive mode (CIC, Boston University). Typical conditions are as follows: capillary = 3000 kV, cone = 35 or 15, source temperature = 120 °C, and desolvation temperature = 350 °C. In addition to spectroscopic characterization, bulk purity of all new compounds was assessed by combustion elemental analysis for C, H, N. Elemental analysis was carried out at the microanalysis lab at University of Illinois Urbana Champaign using Perkin Elmer 2440, Series II with a combustion temperature of ~2000 °C and accuracy of 0.3% abs. Reactions were monitored using aluminum backed silica-gel thin-layer chromatography (TLC) plates (Silicycle, TLA-R10011B-323, Canada) and visualized under low-wavelength light (254 nm) or stained with iodine on silica for visualization with the naked eye. Purification of reactions was performed using silica-gel (Silicycle, P/N: R10030B (SiliaFlash^®^F60, Size: 40–63 μm, Canada) chromatography. The CombiFlash^®^ Rf + Lumen, Teledyne ISCO was used purification of some compounds. Quantification of elemental gold in samples was performed using ICP-OES at the University of Kentucky Energy Research and Training Laboratory. Flow cytometry experiments were performed at the University of Kentucky flow cytometry core facility. Quantum chemical calculations using Gaussian was performed on the University of Kentucky high performance computing (HPC) facility.

**[2**,**3-bis(tert-butylmethylphosphino)quinoxaline]digold(I) (1)** Under normal atmospheric conditions, in a 10 mL scintillation vial was placed (R,R)-(-)-2,3-bis(t-butylmethylphosphino)quinoxaline (15.5 mg, 0.05 mmol). Chloroform (2 mL) was added and the solution (golden) was stirred at room temperature. To the solution was added solid white Au(tht)Cl (15.0 mg, 0.05 mmol). The solution turned brick red instantly. The solution was stirred for about 15 min and monitored by TLC in 100% chloroform. The solution was passed through celite and solvent allowed to evaporate under vacuum. The residue was washed with diethyl ether and filtered. The solid was dried in vacuo to obtain a light purple solid. Yield: 21 mg, 57.1%. ^1^H NMR (400 MHz, chloroform-d) δ 8.21 (dd, J = 6.4, 3.5 Hz, 1 H), 8.01 (dd, J = 6.5, 3.5 Hz, 1 H), 2.19 (d, J = 8.0 Hz, 3 H), 1.43 (d, J = 17.1 Hz, 9 H). ^13^C NMR (101 MHz, chloroform-d) δ 153.36, 153.16, 152.96, 152.74, 152.53, 140.13, 140.09, 140.05, 133.55, 129.39, 35.93, 35.89, 35.74, 35.73, 35.57, 35.53, 28.06, 28.02, 28.00, 11.98, 11.96, 11.79, 11.75, 11.58, 11.56. ^31^P NMR (162 MHz, chloroform-d) δ 29.02. HRMS (ESI) (m/z): calcd. for C_18_H_28_Au_2_Cl_2_N_2_P_2_ [M + Na] 821.0334, found: 821.0362. Anal. Calcd. for C_18_H_28_Au_2_Cl_2_N_2_P_2_: C, 27.05; H, 3.53; N, 3.51. Found: C, 27.13; H, 3.77; N, 3.89.

**Bis-[2**,**3-bis(tert-butylmethylphosphino)quinoxaline]gold(I) chloride (2)** Under normal atmospheric conditions, in a 25 mL round bottom flask was placed (R,R)-(-)-2,3-bis(t-butylmethylphosphino)quinoxaline (39.0 mg, 0.117 mmol). Chloroform (6.0 mL) was added and the solution (golden) was stirred at room temperature. To the solution was added solid white Au(tht)Cl (37.0 mg, 0.115 mmol). The solution turned brick red instantly. The solution was stirred for about 15 min and monitored by TLC in 5% MeOH in DCM. A purple precipitate was obtained. Yield: 26 mg, 23.0%. ^1^H NMR (400 MHz, chloroform-d) δ 8.25 (dd, J = 6.4, 3.5 Hz, 1 H), 7.99 (dd, J = 6.5, 3.4 Hz, 1 H), 2.01 (s, 3 H), 1.28 (s, 9 H). ^31^P NMR (162 MHz, chloroform-d) δ 13.05. HRMS (ESI) (m/z): calcd. for C_36_H_56_AuN_4_P_4_ [M-Cl]^+^ 865.3121, found: 865.3104. Anal. Calcd. for C_36_H_56_AuClN_4_P_4_.0.3CH_2_Cl_2_: C, 47.05; H, 6.16; N, 6.05. Found: C, 47.18; H, 6.27; N, 5.90.

**(2-benzoylpyridine)[(2**,**3-tert-butylmethylphosphino)quinoxaline]gold(III) dichloride (3)** Under normal atmospheric conditions, cyclometalated Au(III)Cl2 (26.1 mg, 0.058 mmol) was placed in a 25 mL round bottom flask and 5 mL of chloroform was added, the solution turned white. (R,R)-(-)-2,3-bis(t-butylmethylphosphino)quinoxaline (19.8 mg, 0.059 mmol) was added, the solution turned golden peach color. The solution was stirred for about 2 hours. The solution was monitored by TLC in 5% MeOH in DCM and Au starting materials disappeared. The product was separated by flash silica-gel chromatography with 5% MeOH in DCM as eluent. A bright purple precipitate was obtained. Yield: 35.8 mg, 73.6%. ^1^H NMR (400 MHz, DMSO-d6) δ 8.80–8.75 (m, 1 H), 8.48 (dd, J = 11.7, 5.7 Hz, 1 H), 8.40–8.20 (m, 3 H), 8.18–8.01 (m, 1 H), 8.01–7.92 (m, 2 H), 7.92–7.83 (m, 1 H), 7.76–7.62 (m, 2 H), 7.41 (q, J = 7.5 Hz, 1 H), 2.68 (d, J = 12.7 Hz, 1 H), 2.47 (s, 2 H), 2.38 (d, J = 11.9 Hz, 1 H), 2.02 (d, J = 12.6 Hz, 2 H), 1.48–1.36 (m, 9 H), 1.15–1.05 (m, 9 H). ^13^C NMR (101 MHz, DMSO-d_6_) δ 198.15, 198.11, 157.15, 155.89, 155.29, 150.28, 149.66, 149.49, 149.00, 148.81, 148.72, 141.97, 141.90, 139.66, 139.6, 137.92, 137.89, 137.37, 136.95, 136.88, 135.37, 135.27, 135.14, 135.02, 134.54, 134.45, 134.18, 129.98, 126.79, 126.52, 126.34, 126.04, 124.58, 124.47, 54.92, 37.91, 37.68, 36.97, 36.73, 27.11, 27.09, 26.53, 26.51, 26.48, 26.45, 25.34, 25.32, 6.43, 6.10, 5.62, 5.01, 4.66, 4.37. ^31^P NMR (162 MHz, CDCl3) δ 50.35 (d, J = 20.1 Hz), 49.39 (d, J = 19.7 Hz), 43.82 (d, J = 19.7 Hz), 37.83 (d, J = 19.8 Hz). HRMS (ESI) (m/z): calcd. for C_30_H_36_AuClN_3_OP_2_ [M-Cl]^+^ 748.1688, found: 748.1685. Anal. Calcd. for C_30_H_36_AuCl_2_N_3_OP_2_.0.5CH_2_Cl_2_.0.8(CH_3_CH_2_)_2_O: C, 45.67; H, 5.12; N, 4.74. Found: C, 46.03; H, 4.96; N, 4.40.

**Bis[μ-[1**,**2-ethanediylbis[diphenylphosphine-κP]]]digold(I) diperchlorate (4)** Under normal atmospheric conditions, HAuCl_4_·3H_2_O (200.0 mg, 0.508 mmol), 1,2-Bis(diphenylphosphino)ethane, DPPE, (424.0 mg, 1.064 mmol), and NaClO_4_ (186.0 mg, 1.519 mmol) were placed in a 100 mL round bottom flask and 40 mL of MeCN was added. The reaction solution was stirred and refluxed overnight. And then it was filtered and the solvent was allowed to evaporate under vacuum. A white precipitate was obtained. Yield: 146.8 mg, 20.8%. ^1^H NMR (400 MHz, DMSO-d_6_) δ 7.80–7.09 (m, 5 H), 2.55 (s, 1 H). ^13^C NMR (101 MHz, DMSO-d_6_) δ 133.22, 132.03, 130.34, 129.33, 128.85. ^31^P NMR (162 MHz, DMSO-d_6_) δ 21.23. HRMS (ESI) (m/z): calcd. for C_52_H_48_Au_2_ClO_4_P_4_ [M-ClO_4_]^+^ 1289.1523, found: 1289.1545.

**Bis[1**,**2-ethanediylbis[diphenylphosphine-κP]]gold(I) perchlorate (5)** Under normal atmospheric conditions, HAuCl_4_·3H_2_O (200.0 mg, 0.508 mmol), 1,2-Bis(diphenylphosphino)ethane, DPPE, (424.0 mg, 1.064 mmol), and NaClO_4_ (186.0 mg, 1.519 mmol) were placed in a 100 mL round bottom flask and 40 mL of MeCN was added. The reaction solution was stirred at room temperature overnight. And then it was filtered and the solvent was allowed to evaporate under vacuum. A white precipitate was obtained. Yield: 230 mg, 41.4%. 1 H NMR (400 MHz, DMSO-d_6_) δ 7.88–7.11 (m, 5 H), 2.55 (s, 1 H). ^13^C NMR (101 MHz, DMSO-d_6_) δ 133.25, 132.03, 130.34, 128.85, 27.55. ^31^P NMR (162 MHz, DMSO-d_6_) δ 21.24. HRMS (ESI) (m/z): calcd. for C_52_H_48_AuP_4_ [M-ClO_4_]^+^ 933.2372, found: 933.2385

**(2-benzoylpyridine)[1**,**2-ethanediylbis[diphenylphosphine]]gold(III) dichloride (6)** Under normal atmospheric conditions, cyclometalated Au(III)Cl_2_ (26.8 mg, 0.060 mmol) was placed in a 25 mL round bottom flask and 5 mL of chloroform was added. 1,2-Bis(diphenylphosphino)ethane, DPPE, (23.7 mg, 0.059 mmol) was added and the reaction solution was refluxed for 4 hours. The reaction was monitored by TLC in 5% MeOH in DCM, the target compound showed an Rf ~0.3. After TLC. The product was separated by flash silica-gel chromatography with 5% MeOH in DCM as eluent. Yield: 19.4 mg, 38.9%. ^1^H NMR (400 MHz, Chloroform-d) δ 8.59 (d, J = 4.7 Hz, 1 H), 8.22–8.06 (m, 2 H), 7.95 (m, J = 15.3, 5.9 Hz, 6 H), 7.83–7.73 (m, 5 H), 7.72–7.34 (m, 14 H), 3.79 (s, 2 H), 3.18 (s, 2 H). ^13^C NMR (101 MHz, DMSO-d_6_) δ 193.98, 151.88, 148.73, 141.73 (d, J = 6.4 Hz), 137.60 (d, J = 16.4 Hz), 134.32, 133.47–132.48 (m), 131.54, 129.69, 129.11, 127.99, 124.55, 120.11, 119.24, 117.95, 117.10, 79.20, 24.49, 22.09. ^31^P NMR (162 MHz, DMSO-d_6_) δ 27.87, 27.46. HRMS (ESI) (m/z): calcd. for C_38_H_32_AuClNOP_2_ [M-Cl]^+^ 812.1313, found: 812.1315. Anal. Calcd. for C_38_H_32_AuCl_2_NOP_2_: C, 53.79; H, 3.80; N, 1.65. Found: C, 53.51; H, 3.90; N, 1.46.

**Dichloro(2-benzoylpyridine)gold(III) (7)** This procedure was adapted from previous reports in the literature^93–95^. ^1^H NMR (400 MHz, DMSO-d6) δ 9.48 (d, J = 5.8 Hz, 1 H), 8.56 (t, J = 7.7 Hz, 1 H), 8.37 (d, J = 7.7 Hz, 1 H), 8.14–8.05 (m, 1 H), 7.76 (d, J = 7.6 Hz, 1 H), 7.73–7.65 (m, 1 H), 7.48 (m, 7.51–7.45, 2 H). LRMS (ESI, positive mode): calcd. for C_12_H_8_AuCl_2_NO [M^+^] 448.96, found: 449.00.

### UV-Vis spectrophotometric measurements

Stock solutions (1 mM) of complexes **1**–**7** were prepared in DMSO. For studies with PBS and DMEM, the gold solutions were diluted with the respective diluent (PBS or DMEM) to achieve a final gold concentration of 25 μM or 50 μM. Background scans were performed with the corresponding DMSO or solvent mixture. For the reactivity experiment with BSA, the sample solution was prepared by the above-mentioned method, and BSA was separately dissolved in PBS to prepare 1 mM stock solution and diluted with PBS. The sample solution was mixed with the BSA solution immediately before the measurement. Single scan or time-based scanning was performed as needed. The UV-Vis spectrometer used was a Shimadzu UV-1280 model.

### Electrochemistry

Cyclic voltammetry was performed in dimethyl sulfoxide (DMSO) with NaClO_4_, which were purchased (Aldrich, USA) and used without further purification. All complexes (**1**−**6**) were dissolved in DMSO in the concentration of 1.0 mM, followed by addition of 0.01 M NaClO_4_ as a supporting electrolyte. Electrochemical measurements were performed at ambient temperature using CH-600D potentiostat (CH Instruments, USA) for cyclic voltammetry. The voltammetric measurements were performed in a three-electrode cell containing a platinum working electrode, a non-aqueous Ag/AgCl reference electrode and a platinum wire as counter electrode.

### Cell viability assay

Various established human ovarian, leukemia and lung cancer cell lines were seeded in 96-well plates (2 × 10^3^ cells/well) and were incubated with RPMI 1640 supplemented with 10% FBS (150 μL) for 24 h and at 37 °C. They were then treated with cisplatin, auranofin, or **1**–**6** at increasing concentrations for 72 h from stock solutions following serial dilutions. Cisplatin stock solution was prepared using PBS and the gold compounds were prepared in DMSO. Thereafter, cellular viability was assessed via the established crystal violet colorimetric assay. In brief, crystal violet reagent (50 μL of a 0.5% solution in glutaraldehyde) was added to each well and allowed to incubate with cells for 30 min. The plates were washed with water by running them under a gentle flow of tap water. The plates were air dried and dissolved with methanol (100 μL/well) and the plates were rocked for an additional 10 min. Measurements of absorbance were subsequently performed using a Genios plate reader at 570 nm (peak absorbance). All experiments were conducted in triplicate. For K562 cells, a cell density of 4,000 cells/well was used and cellular viability was assessed after treatment, using the established luminescent cell titre glo assay.

### Whole cell uptake studies

OVCAR8 cells (1 × 10^6^) were seeded in a 6-well plate and incubated for 15 h at 37 ^o^C. Cells were then incubated with the test compounds (5 μM) in fresh RPMI 1640 medium (10 mL) and subsequently incubated for a given period of time (~24 h) at 37 °C. The medium was then removed and cells were collected via trypsinization. Cells were then washed with PBS (1 mL x 3). The cells were digested by adding 0.5 mL of concentrated HCl and briefly placing them on an agitator. Cells were then transferred to a new tube containing 4.5 mL of DI water. The gold content was analyzed by ICP-OES to obtain the whole cell uptake after quantification.

### Intracellular distribution

To measure the intracellular cellular uptake, ~1 million OVCAR8 cells were treated with 5 μM compounds **1**, or **3** at 37 °C for ~17 h. The media were removed, and the cells were washed with PBS solution (1 mL × 3), harvested, and centrifuged. The cell pellet was suspended in an appropriate volume of PBS to obtain a homogeneous suspension (100 μL). The nuclear and cytoplasmic extraction kit (NE-PER, Thermo Scientific Inc.) was used to extract the separate cytoplasmic, nuclear, and pelleted fractions. The fractions were mineralized with 70% HNO_3_ and then heated at 95 °C for 10 min. The gold content was analyzed by ICP-OES. Cellular gold levels were expressed as pmol of Au per million cells. Results are presented as the mean of three determinations for each data points.

### Cell cycle analysis

OVCAR8 cells were seeded in 6-well plates (2 × 10^5^ cells/well) and were treated with PBS, **1**, or **3** for 24 or 48 h at a concentration of 2 µM. The cells were re-suspended in ice-cold PBS, fixed with 70% ethanol in PBS at 4 °C overnight, subsequently washed with ice-cold PBS (x2), and then incubated with RNase A (1 μg/mL) for 20 min at 37 °C. They were then stained with PI (10 μg/mL for 30 min in the dark) and their DNA content and cell cycle distribution were measured, using a FACSCalibur flow cytometry (BD Biosciences, USA) and as determined with ModFit software.

### Apoptosis study

OVCAR8 cells were seeded in 6-well plates (5 × 10^5^ cells/well), treated with PBS, cisplatin, auranofin, **1** or **3** at a fixed Au concentration (2 µM) for 48 h, and harvested by trypsinization. The Annexin V-FITC Apoptosis Detection Kit (BD Biosciences) was used to determine the fraction of cells (from a total of 1 × 10^4^ cells) that underwent apoptosis, using fluorescence-activated cell-sorting sorting (BD Biosciences, USA) and by following the manufacturer’s protocol. Data were analyzed using FlowJo software.

### Mitochondria membrane potential

OVCAR8 cells (175,000 cells/well) were plated in 6-well plates taking into account respective controls (untreated and unstained, untreated and stained) and test compounds. After overnight incubation of cells at, 37 °C and 5% CO_2_, 5 μM of complexes was added to the desired wells and incubated overnight. We collected cells via trypsinization and centrifugation, and re-suspended cells in a solution of 70% ethanol in PBS and placed in freezer for at least 5 minutes. Rhodamine-123 solution (1 mg/ml in ethanol) was dissolved and diluted with PBS to a final concentration of 0.005 mg/mL. The solution was added (0.5 mL) to the cells re-suspended, and incubated for 30 min. Cells were pelleted by centrifugation, washed with 1 mL of PBS, and re-suspended in 0.5 mL of PBS for flow cytometry analysis.

### Western blot

OVCAR8 cells (500,000 cells/well) were plated on a 6-well plates. The cells were then treated with 5 μM gold compounds and incubated for 24 h and 48 h at 37 °C, after which media was removed and cells were washed with PBS. The cells were scraped into SDS-PAGE loading buffer (64 mM Tris-HCl (pH 6.8)/9.6% glycerol/2% SDS/5% β-mercaptoethanol/0.01% bromophenol blue) and incubated at 95 °C on a heat block for 10 min. The samples were cooled and stored at −20 °C until ready for use. Whole cell lysates were resolved by 4–20% sodium dodecylsulfate polyacrylamide gel electrophoresis (SDS-PAGE; 200 V for 25 min) followed by electro transfer to a polyvinylidene difluoride membrane, PVDF (350 mA for 1 h). Membranes were blocked using 5% (w/v) bovine serum albumin (BSA) in PBST (PBS/0.1% Tween 20) and incubated with specific primary antibodies (Cell Signaling Technology and Santa Cruz Biotechnology) overnight at 4 °C. On the following day, after washing with PBST (3 × 5 mL), the membrane was incubated with horseradish peroxidase-conjugated secondary antibodies (Cell Signaling Technology) in fresh BSA blocking solution. Immune complexes were detected with the ECL detection reagent (BioRad) and analyzed using a BioRad imager fitted with a chemiluminescence filter. Intracellular ROS using murine polymorphonuclear neutrophils.

### X-ray crystallography

Crystals of **1**, **2**, **4**, and **5** were grown at room temperature by vapor diffusion of diethyl ether into a DMF or DCM solutions of each complex. Suitable crystals were selected by microscopic examination through crossed polarizers, mounted on a fine glass fibre in polyisobutane oil, and cooled to 90 K under a stream of nitrogen. A Bruker D8 Venture diffractometer with graded-multilayer focused MoKα X-rays (λ = 0.71073 Å) was used to collect the diffraction data from the crystal. The raw data were integrated, scaled, merged and corrected for Lorentz-polarization effects using the APEX3 package^96–98^. Space group determination and structure solution and refinement were carried out with SHELXT and SHELXL^99,100^, respectively. All non-hydrogen atoms were refined with anisotropic displacement parameters. Hydrogen atoms were placed at calculated positions and refined using a riding model with their isotropic displacement parameters (U_iso_) set to either 1.2U_iso_ or 1.5U_iso_ of the atom to which they were attached. The structures, deposited in the Cambridge Structural Database, were checked for missed higher symmetry, twinning, and overall quality with PLATON^101^, an R-tensor^102^, and finally validated using CheckCIF^101^. See Tables S1–S8 for structure details. Of note is the different counter anions (ClO4^−^) for **4** and **5**, which make them crystallographically distinct from similar published structures.

### Cell lines and cell culture conditions

All ovarian cancer cells (OVCAR8), lung cancer cells (H460), and the leukemia cell line (K562) were maintained in the Roswell Park Memorial Institute (RPMI) 1640 medium. With the exception of H460 which was supplemented with 15% heat inactivated fetal bovine serum (FBS) and 10% glutamine, all other cell lines were cultured in RPMI supplemented with 10% FBS and 1% penicillin/streptomycin. All cells were grown at 310 K in a humidified atmosphere containing 5% CO_2_.

### Quantum chemical calculations

All calculations were performed using the Gaussian 09 package^75^. Density functional theory (DFT) and time dependent density functional theory TD-DFT with Becke’s three-parameter hybrid functional with the correlation functional of Lee, Yang, and Parr (B3LYP)^103^ was used to calculate the equilibrium geometries and vibrational frequencies of **1**–**6**. Stuttgart-Dresden effective-core-potential basis set (SDD) for Au and 6–31 G(d,p) for C, H, N, O, P, and Cl were employed for these calculations. A vibrational analysis was accomplished to identify the nature of each optimized stationary points no imaginary frequencies were found. Optimized XYZ coordinates for all calculated molecules are attached on Table S9.

## Associated Content

Supporting Information is available free of charge on the Nature Publications website. Experimental details, characterization of compounds **1**–**7**, and data regarding all X-ray crystallography, molecular biology. Crystal structures of **1** (CCDC 1817925), **2** (CCDC 1817924), **4 (**CCDC 1852670), and **5** (CCDC 1852671) have been deposited at the Cambridge Crystallographic Data Centre.

## Supplementary information

ESI
