# Supplementary material for: Gold(I/III)-Phosphine Complexes as Potent Antiproliferative Agents
Source: Sci Rep. 2019 Aug 26;9:12335. doi: 10.1038/s41598-019-48584-5 (PMC6710276; doi:10.1038/s41598-019-48584-5)
Supplement: Supplementary file 1 — ESI [file 41598_2019_48584_MOESM1_ESM.pdf]

Gold(I/III)-Phosphine Complexes as Potent Anticancer Agents

Jong Hyun Kim, Evan Reeder, Sean Parkin, and Samuel G. Awuah*

*To whom correspondence should be addressed:

Samuel G. Awuah, Ph.D.
Assistant Professor of Chemistry & Pharmaceutical Sciences
Department of Chemistry
University of Kentucky
505 Rose Street
Lexington, KY 40509, USA
(859) 323-9561
awuah@uky.edu

TABLE OF CONTENTS

NMR spectra data S3-S14
Supplementary figures S15-S42
Supporting Tables S43-S80

NMR spectra data

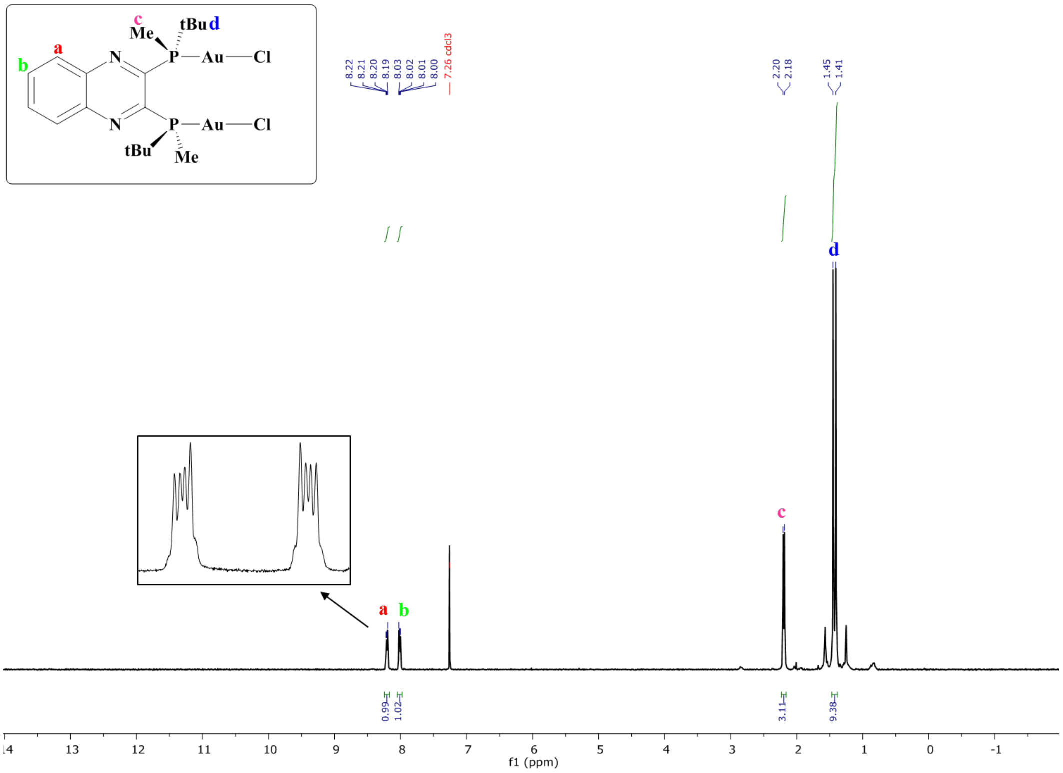

Figure S1. ^{1}H NMR spectrum of complex **1** in $CDCl_3$ at 298K

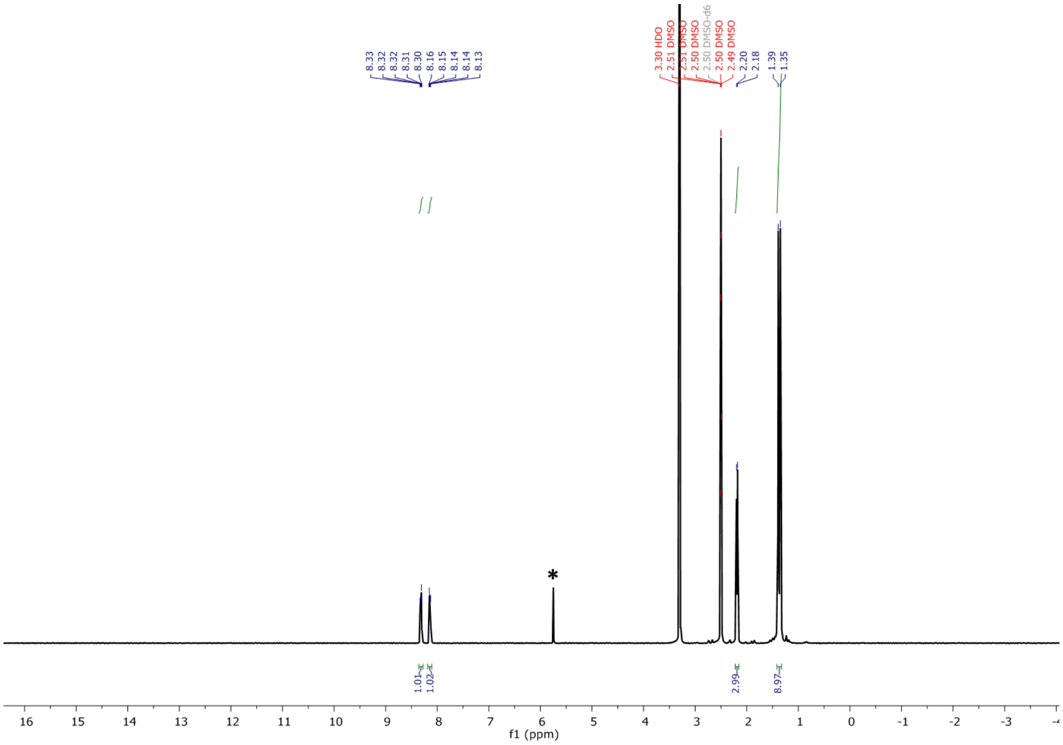

Figure S2. ^{1}H NMR spectrum of complex **1** in DMSO at 298K * Impurity(CH_2Cl_2)

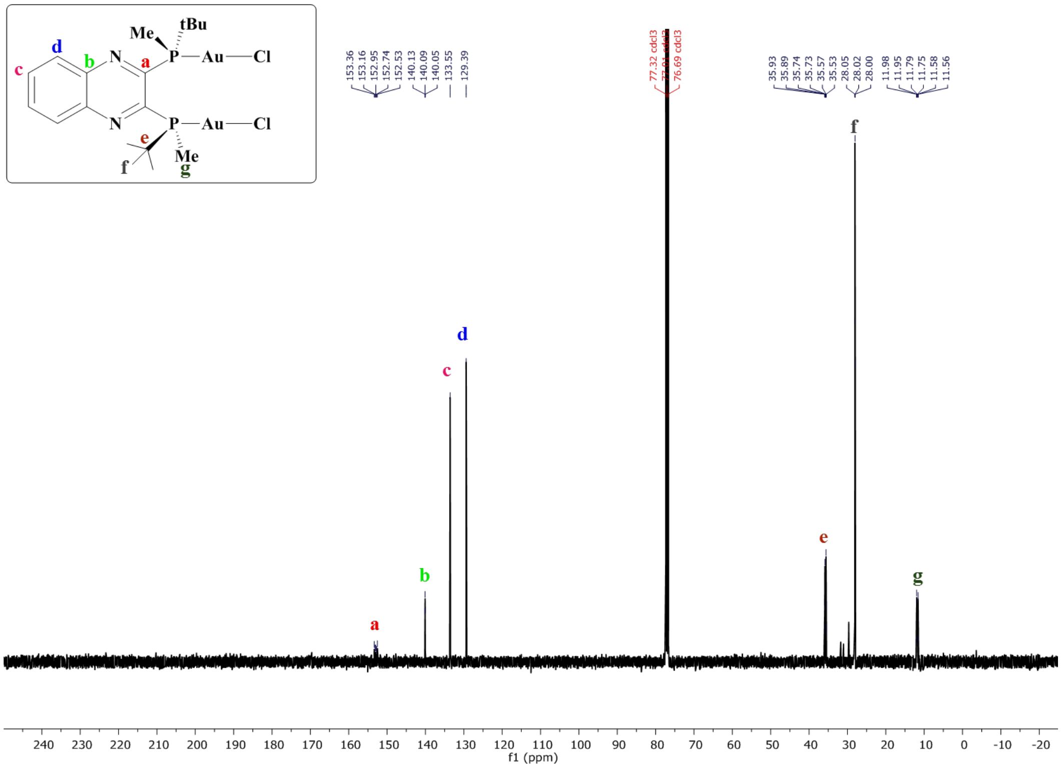

Figure S3. $^{13}C\{^{1}H\}$ NMR spectrum of complex **1** in $CDCl_3$ at 298K.

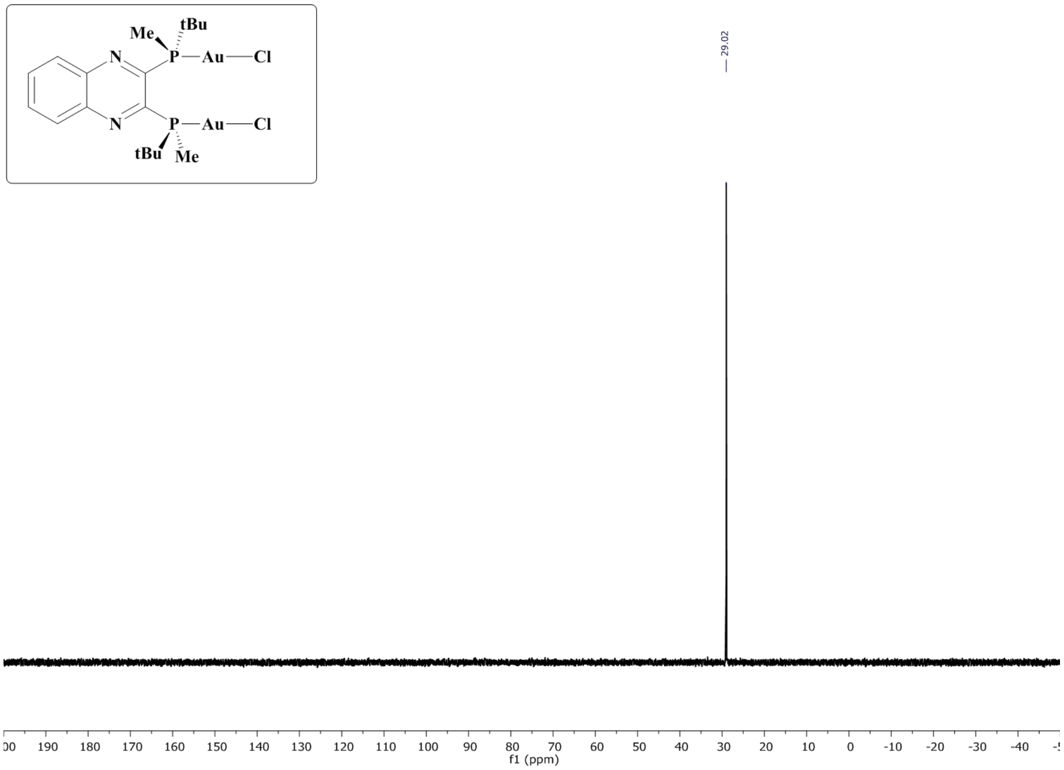

Figure S4. $^{31}P\{^{1}H\}$ NMR spectrum of complex **1** in $CDCl_3$ at 298K

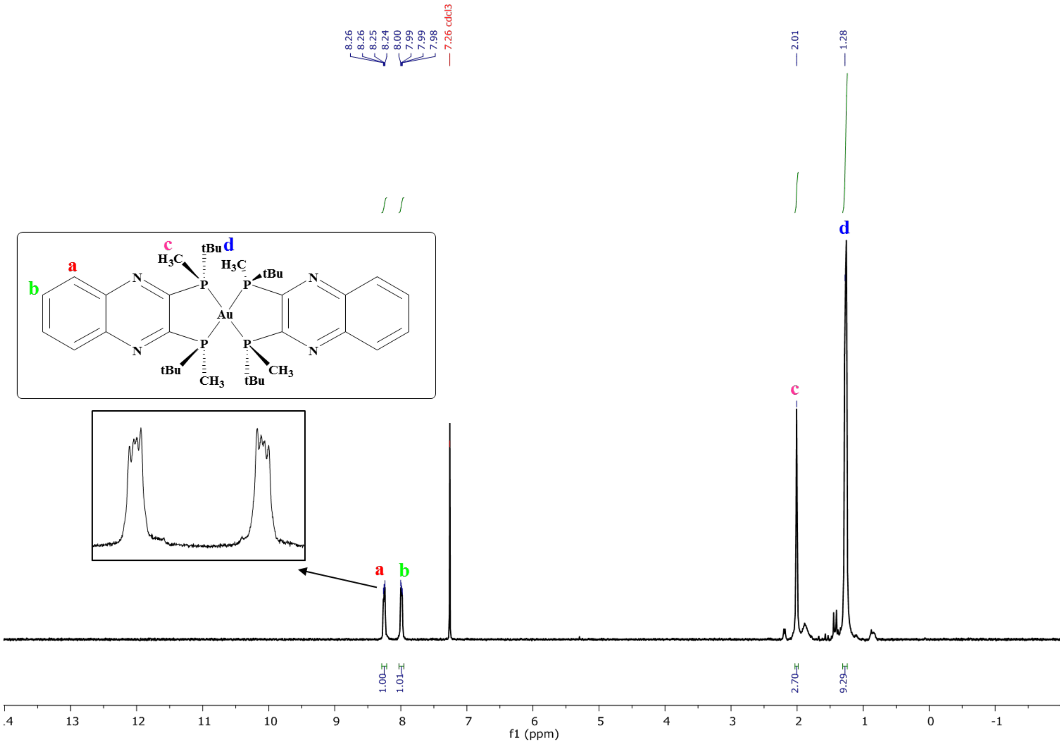

Figure S5. ^{1}H NMR spectrum of complex **2** in $CDCl_3$ at 298K

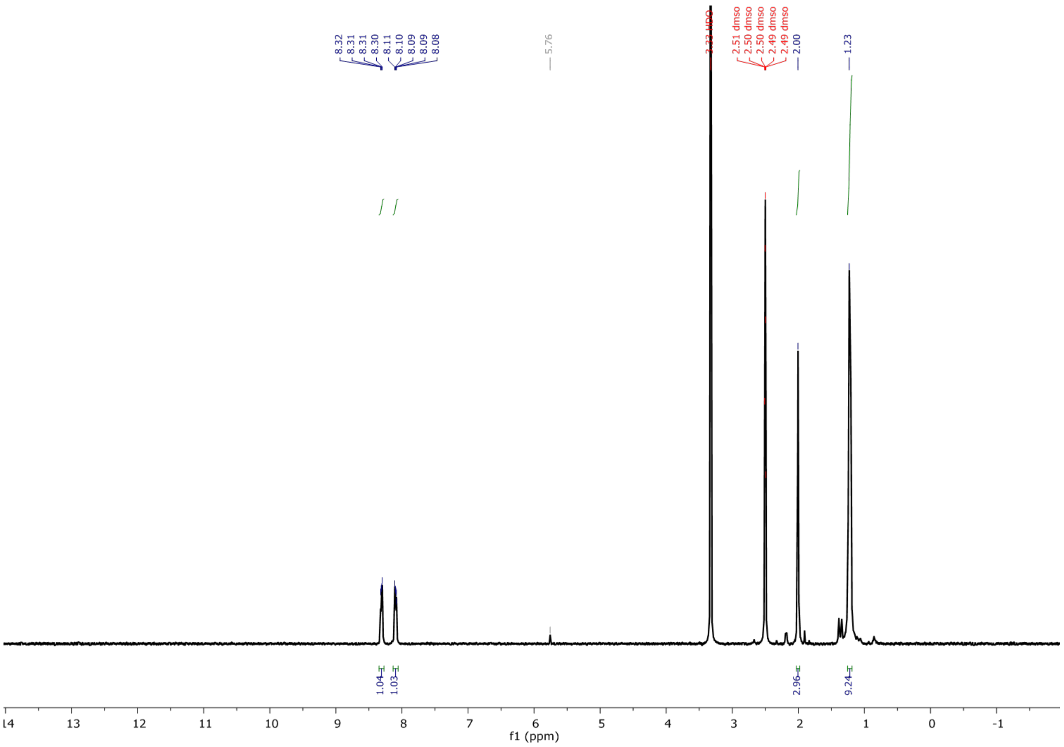

Figure S6. ^{1}H NMR spectrum of complex **2** in DMSO at 298K

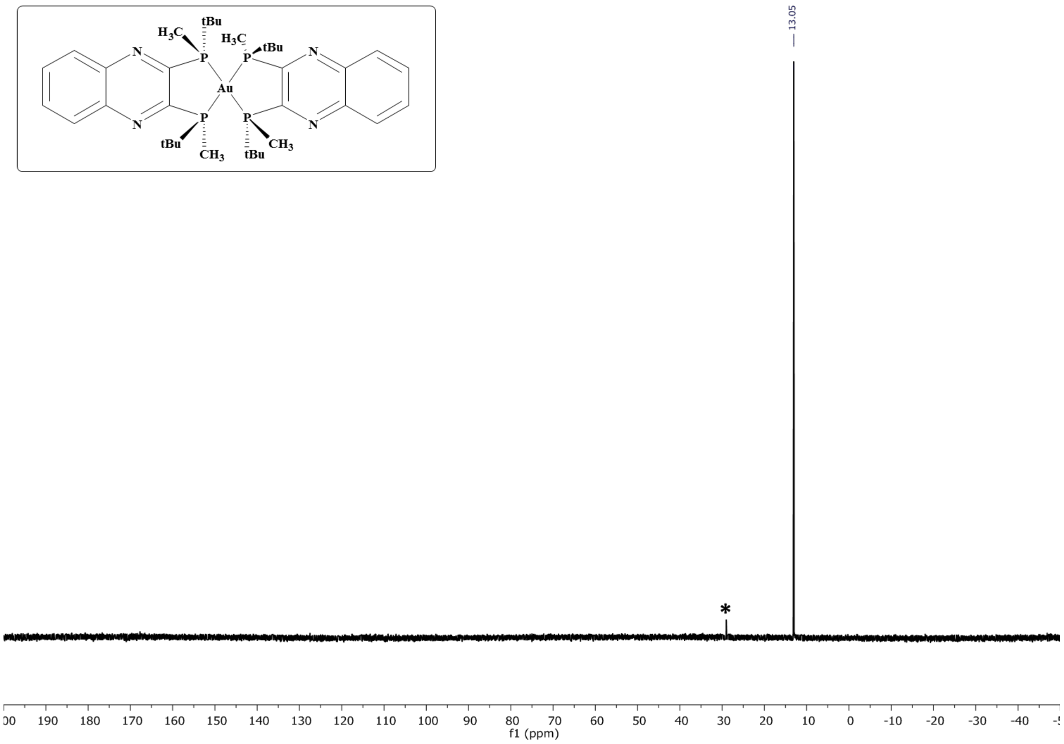

Figure S7. $^{31}P\{^{1}H\}$ NMR spectrum of complex **2** in $CDCl_3$ at 298K. * Impurity (complex 1).

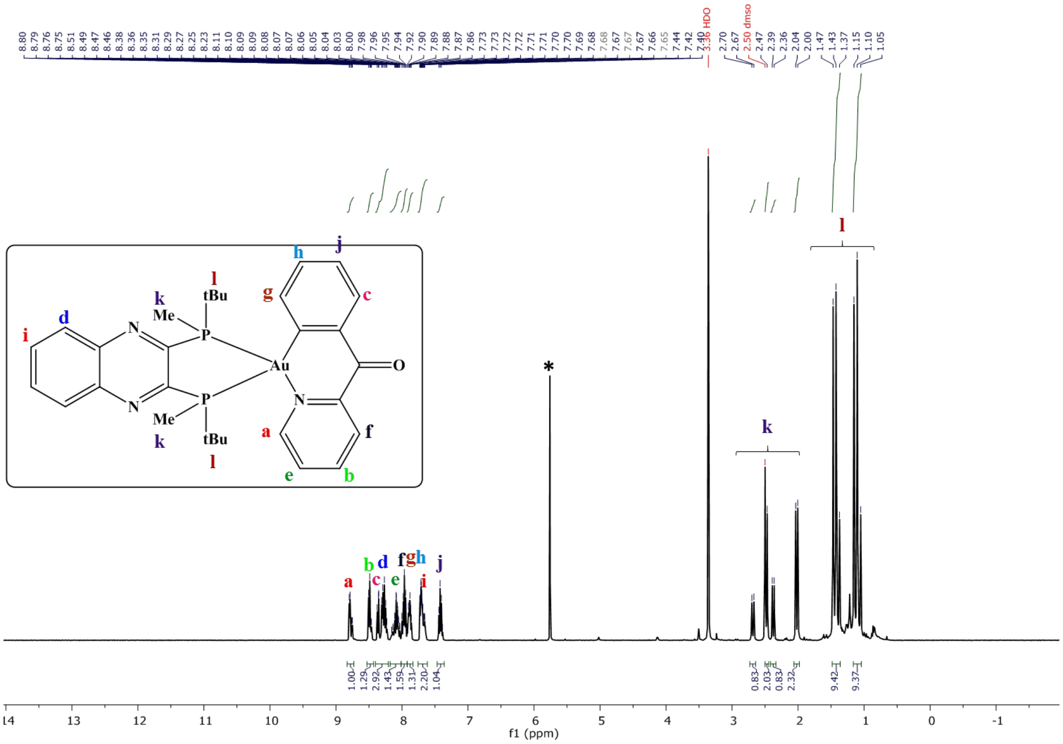

Figure S8. ^{1}H NMR spectrum of complex **3** (diastereomers) in DMSO-d_6 at 298K. * CH_2Cl_2.

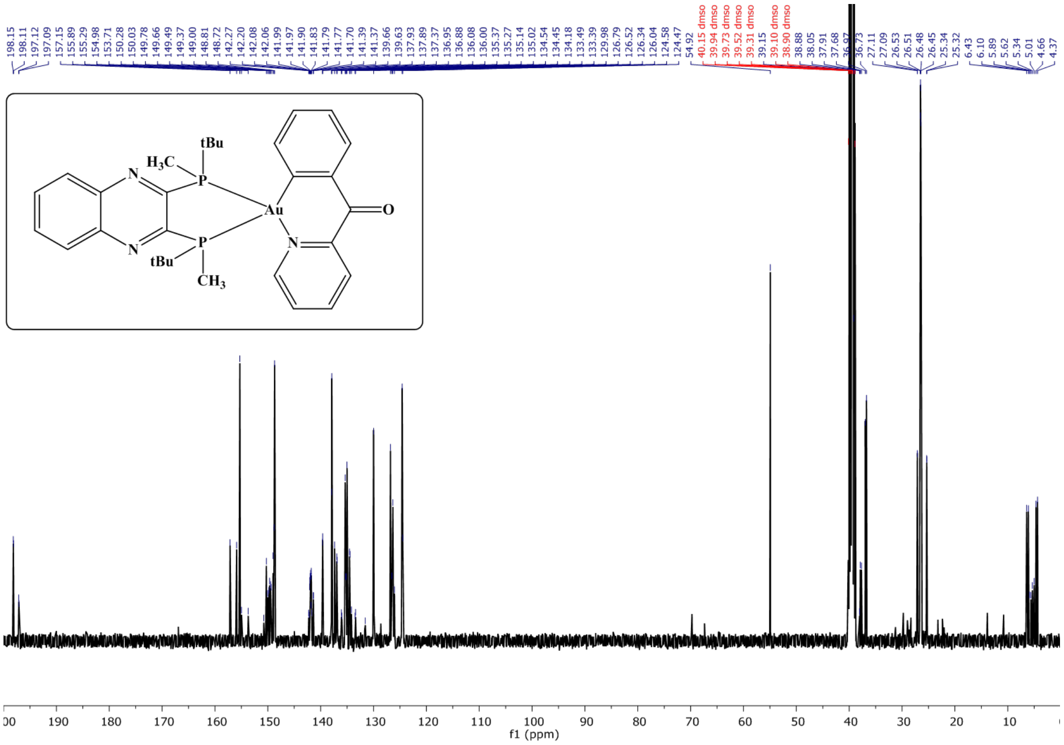

Figure S9. $^{13}C\{^1H\}$ NMR spectrum of complex **3** (diastereomers) in DMSO-d_6 at 298K.

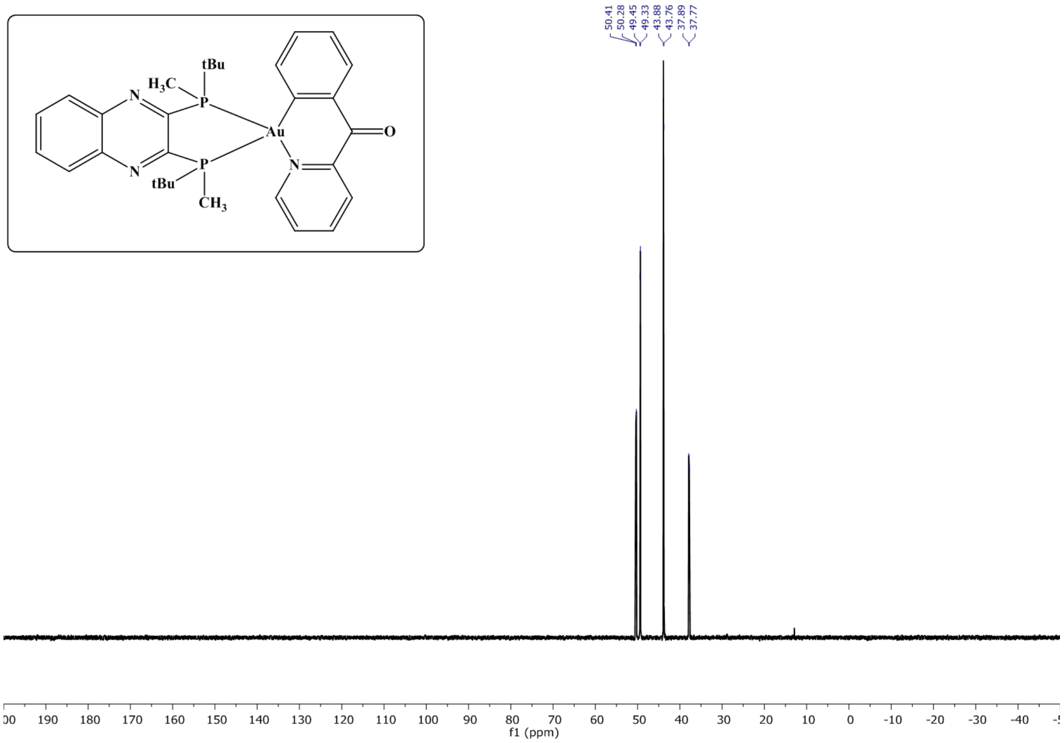

Figure S10. $^{31}P\{^1H\}$ NMR spectrum of complex **3** (diastereomers) in $CDCl_3$ at 298K.

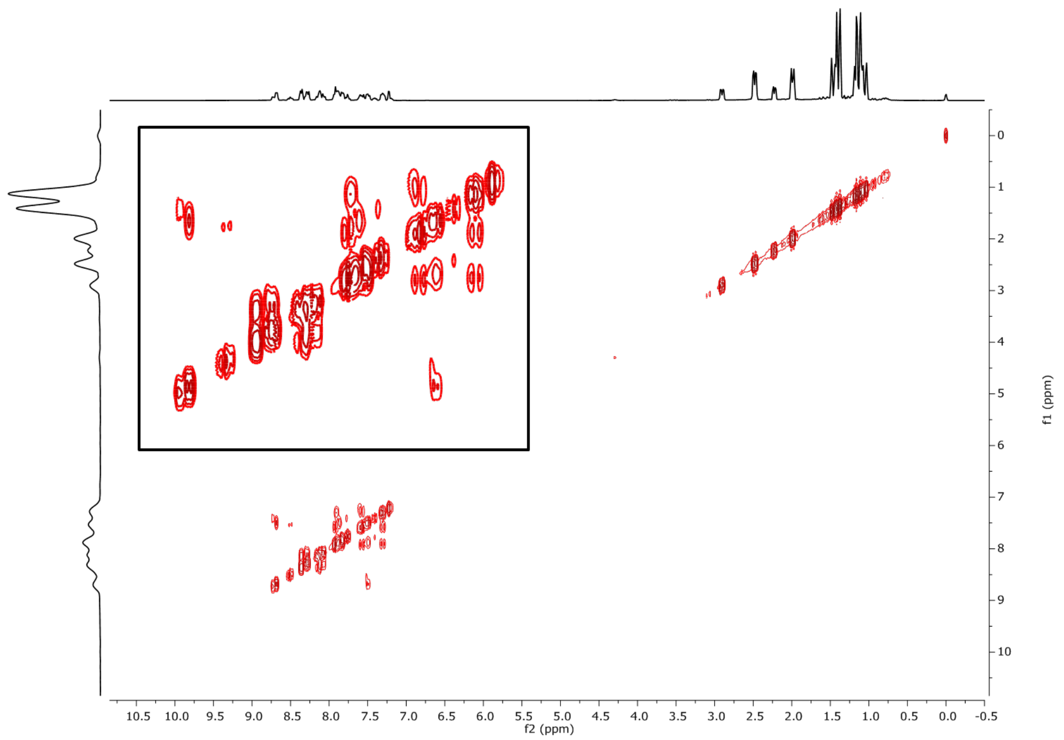

Figure S11. COSY NMR spectrum of complex **3** in $CDCl_3$ at 298K.

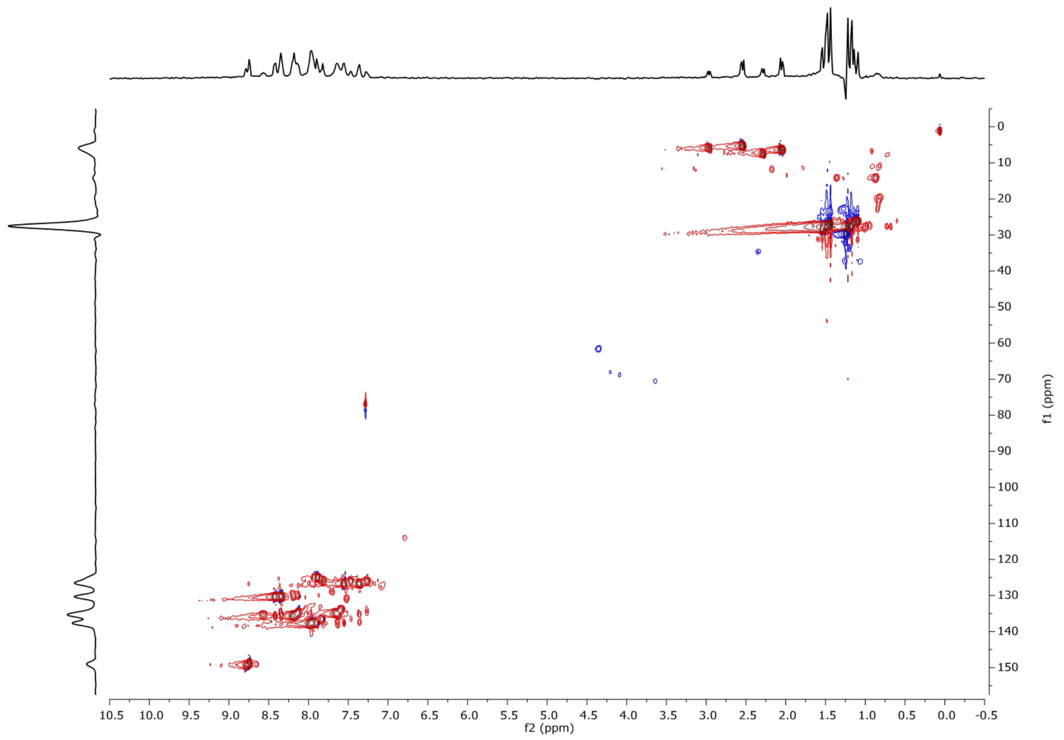

Figure S12. ^{1}H-^{13}C HSQC NMR spectrum of complex **3** in $CDCl_3$ at 298K.

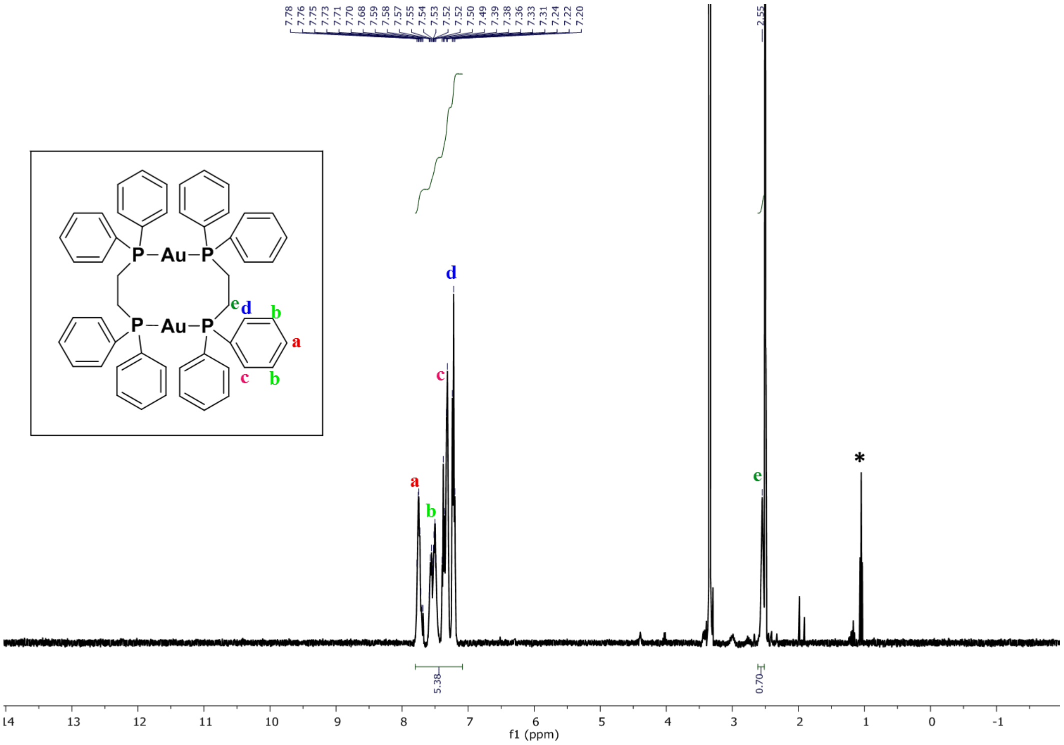

Figure S13. ^{1}H NMR spectrum of complex **4** in DMSO-d_6 at 298K. * Ethanol.

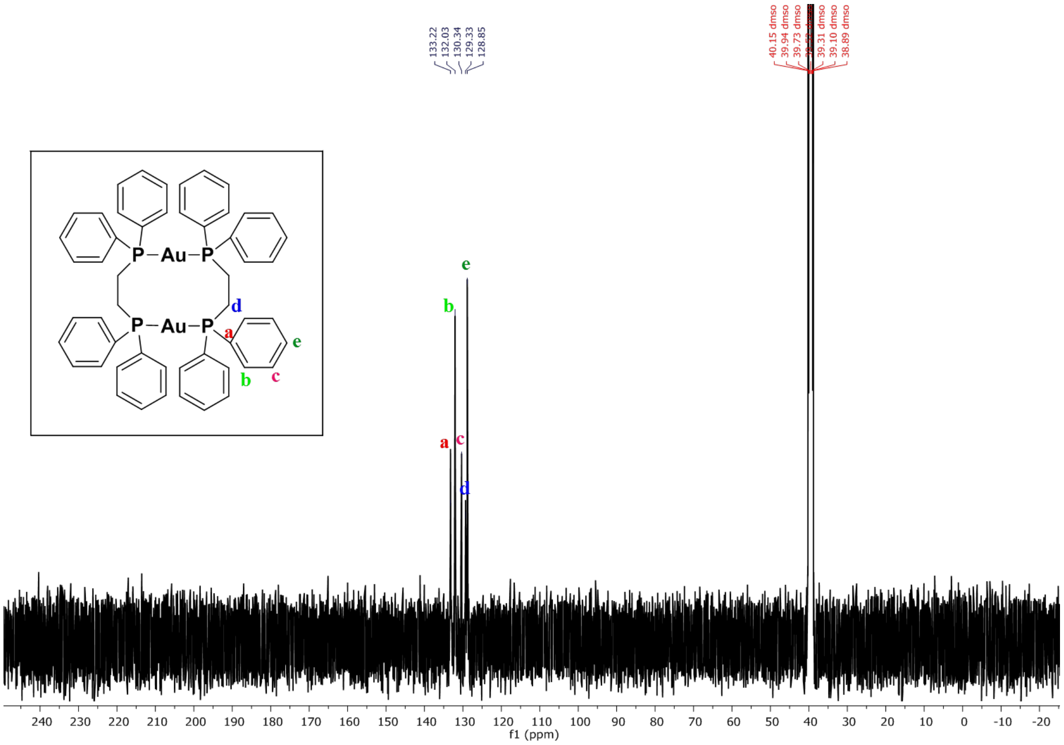

Figure S14. $^{13}C\{^{1}H\}$ NMR spectrum of complex **4** in DMSO-d_6 at 298K.

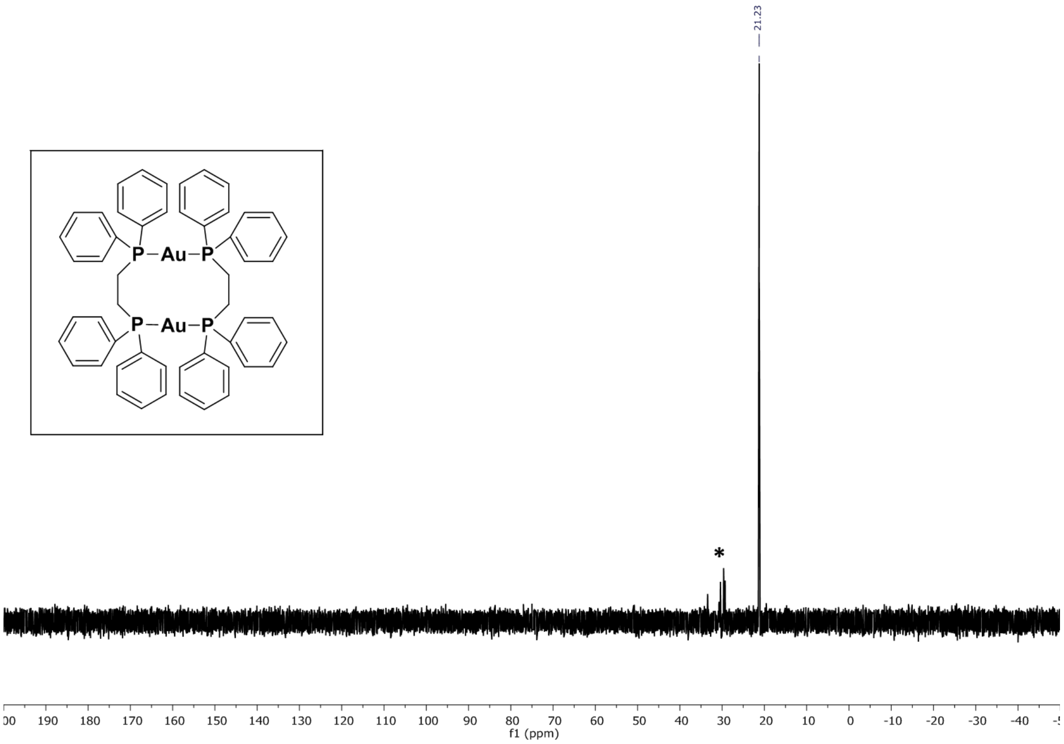

Figure S15. $^{31}P\{^{1}H\}$ NMR spectrum of complex **4** in DMSO-d$_6$ at 298K. * Impurity from DPPE.

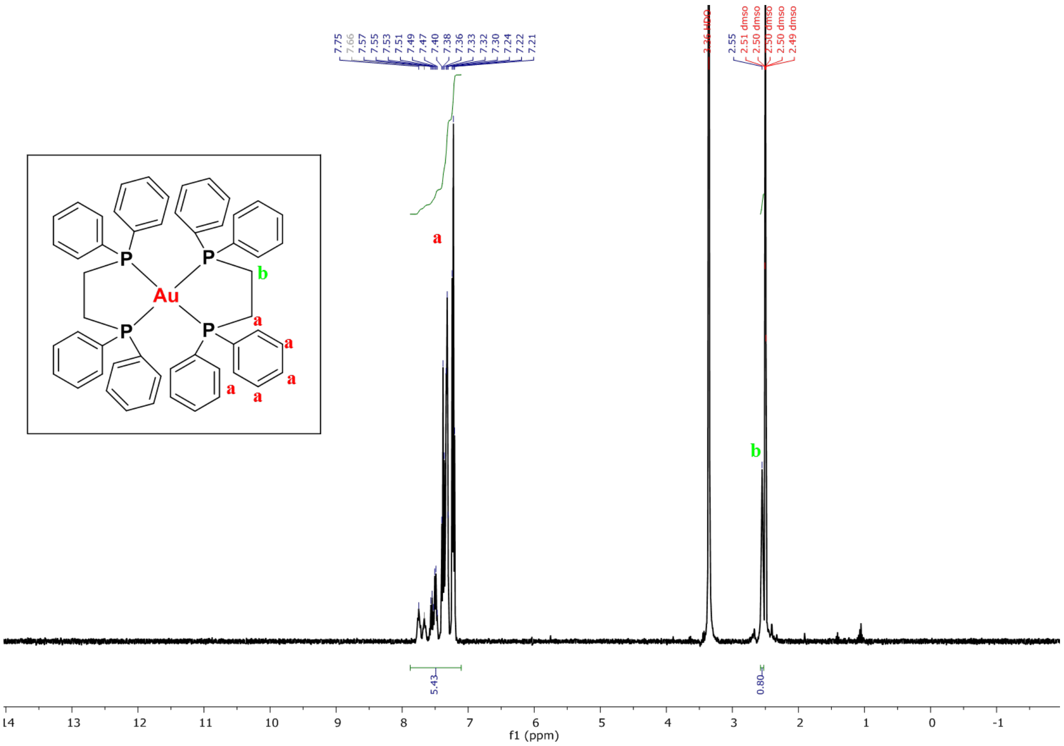

Figure S16. ^{1}H NMR spectrum of complex **5** in DMSO-d$_6$ at 298K.

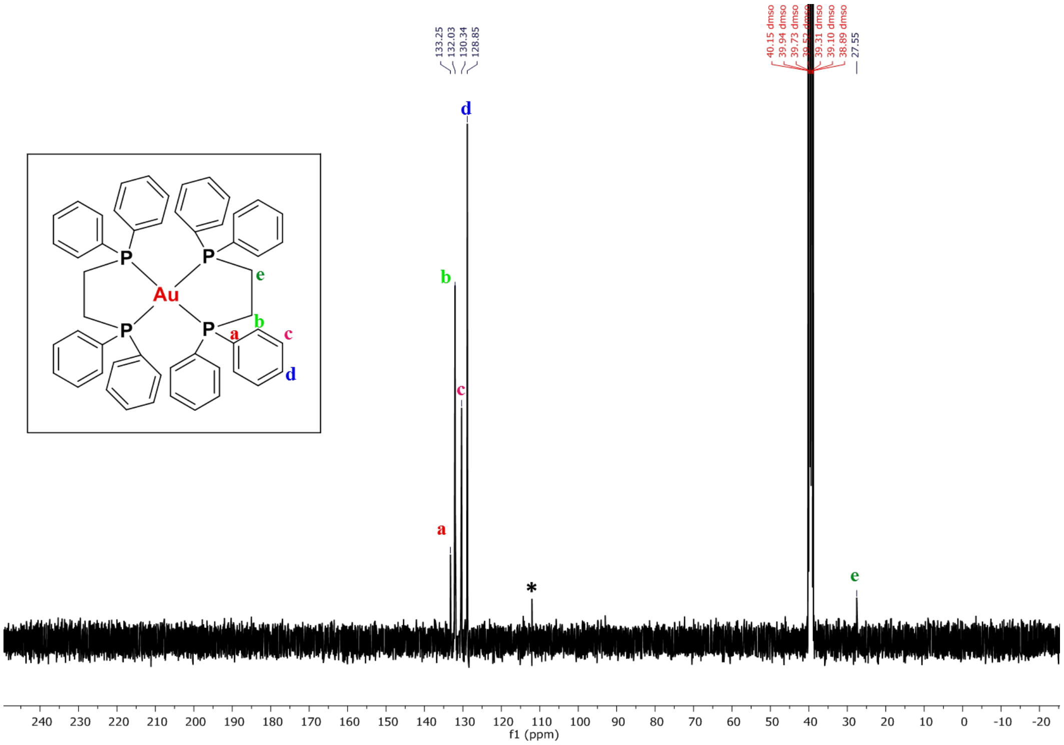

Figure S17. $^{13}C\{^1H\}$ NMR spectrum of complex **5** in DMSO-d_6 at 298K. *Mechanical noise.

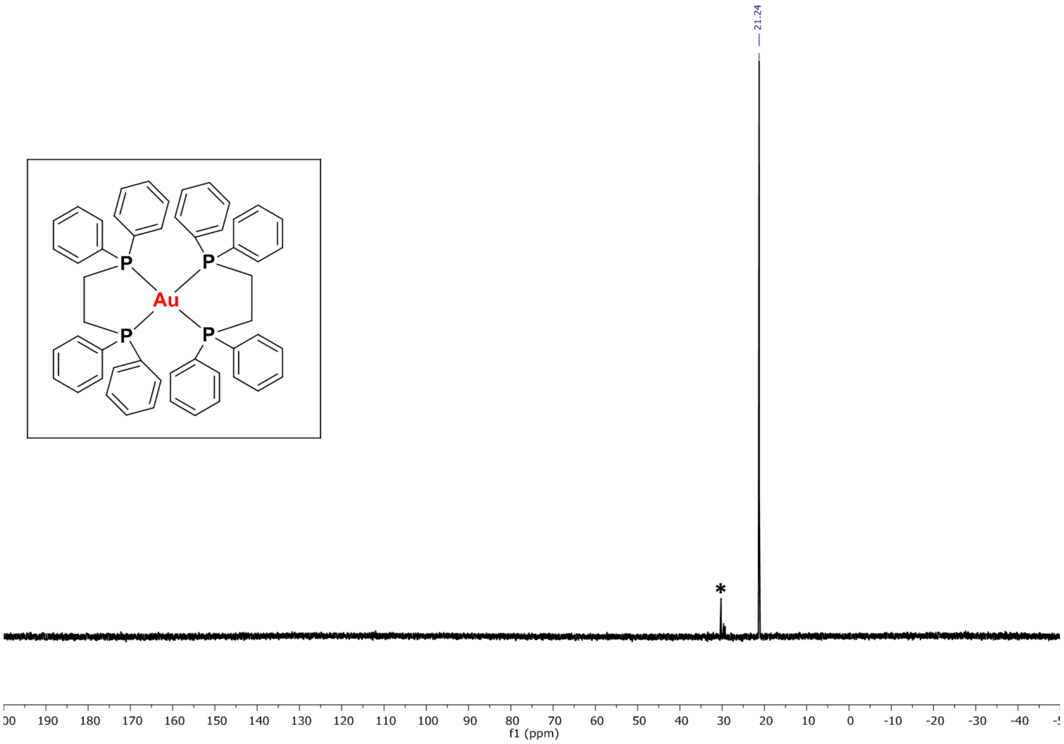

Figure S18. $^{31}P\{^1H\}$ NMR spectrum of complex **5** in DMSO-d_6 at 298K. *Impurity from DPPE.

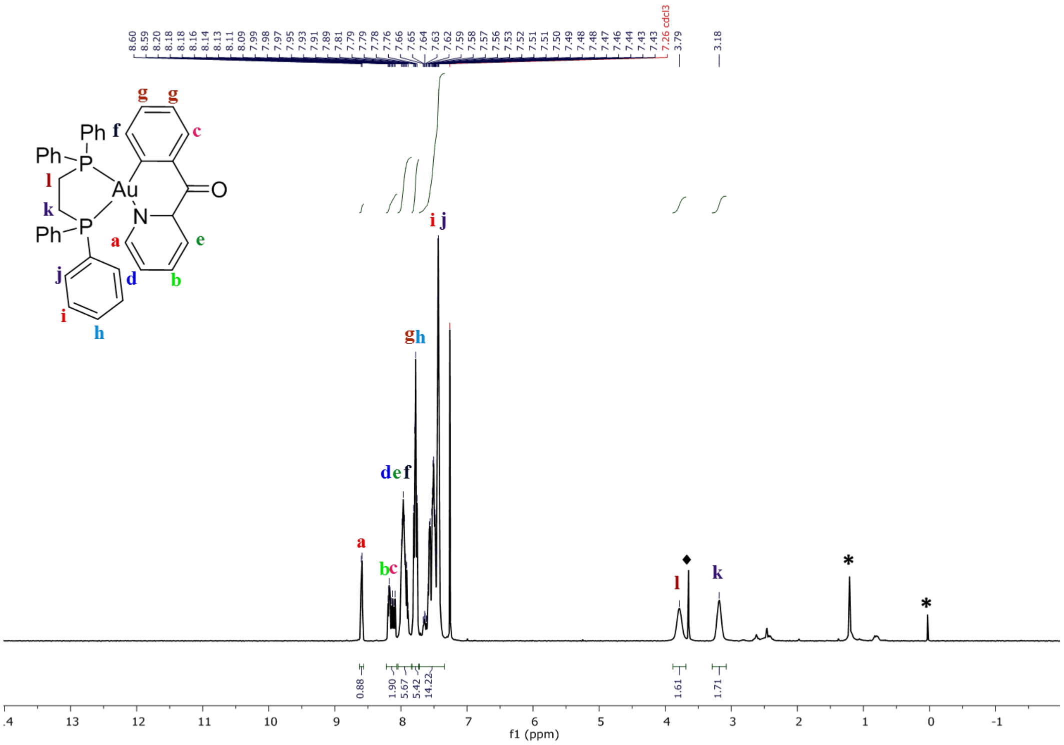

Figure S19. ^{1}H NMR spectrum of complex **6** in $CDCl_3$ at 298K. * grease, ♦ impurity.

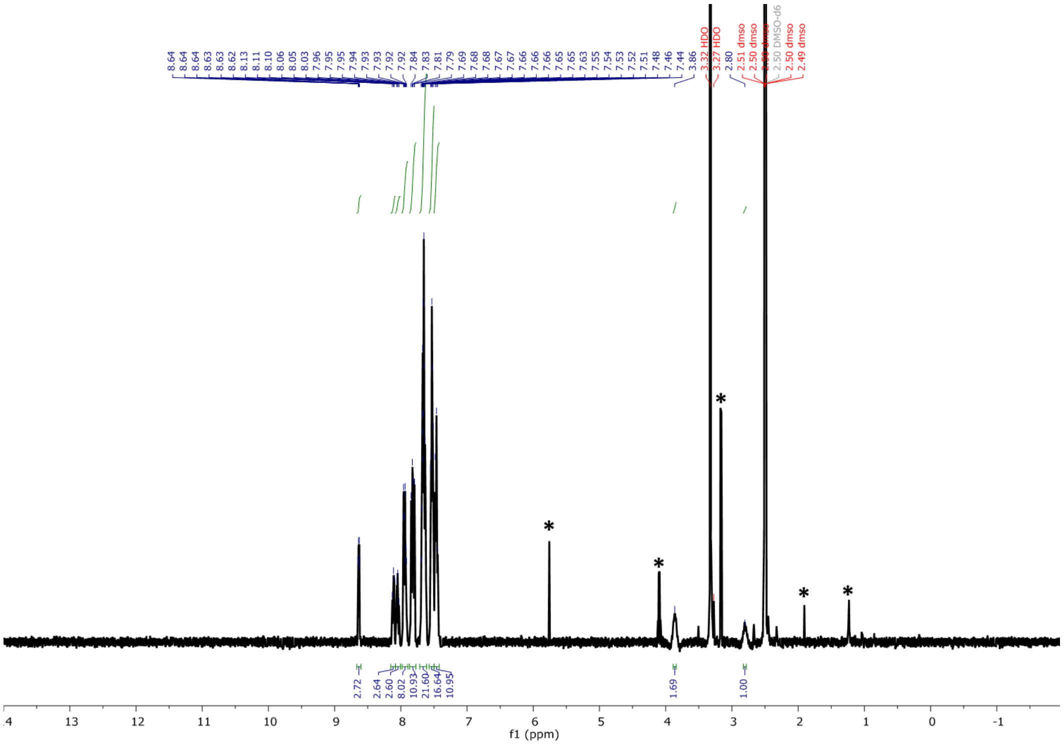

Figure S20. ^{1}H NMR spectrum of complex **6** in DMSO at 298K. * impurities(solvents).

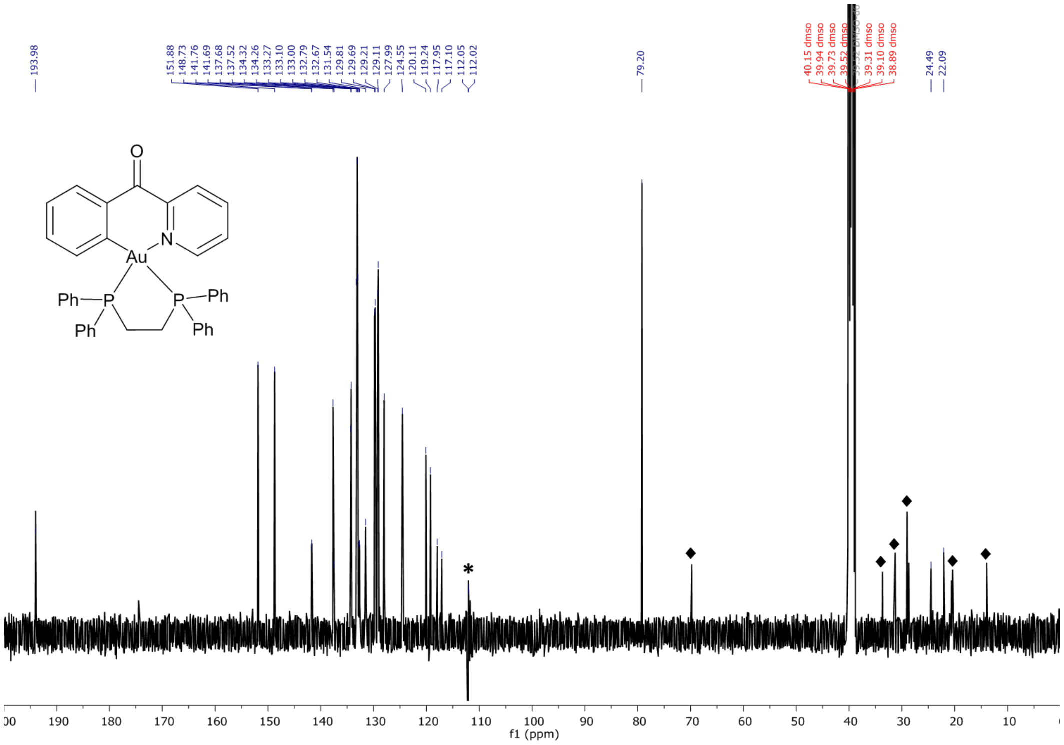

Figure S21. $^{13}C\{^1H\}$ NMR spectrum of complex **6** in DMSO at 298K. *Mechanical noise, ♦impurities.

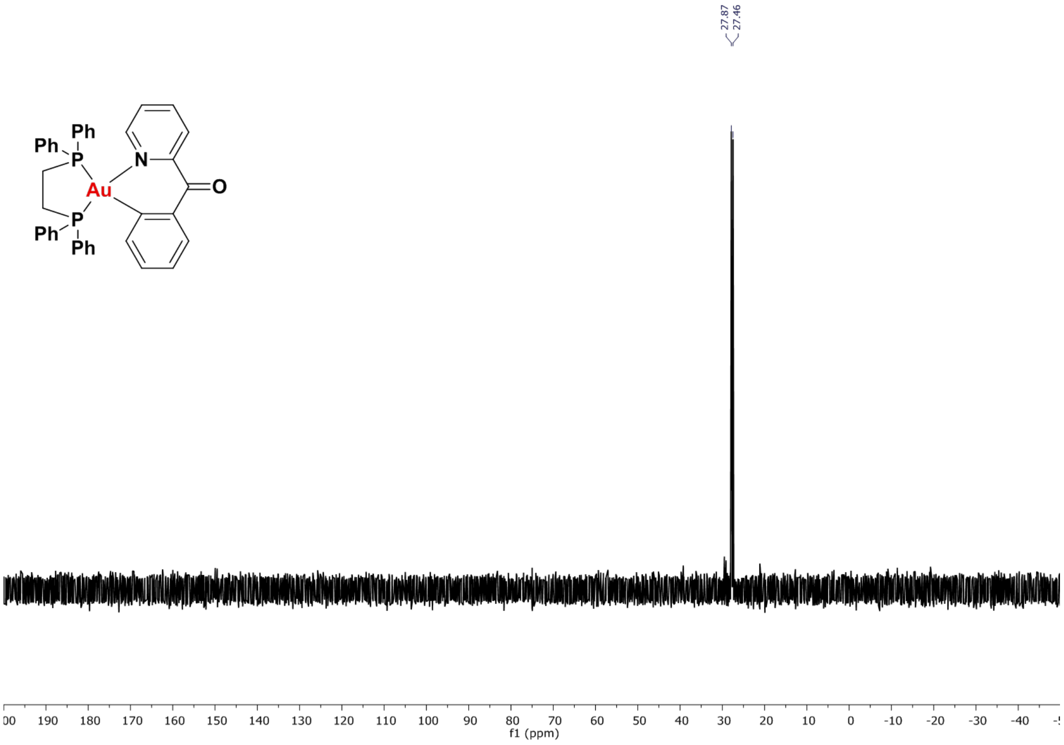

Figure S22. $^{31}P\{^1H\}$ NMR spectrum of complex **6** in DMSO at 298K.

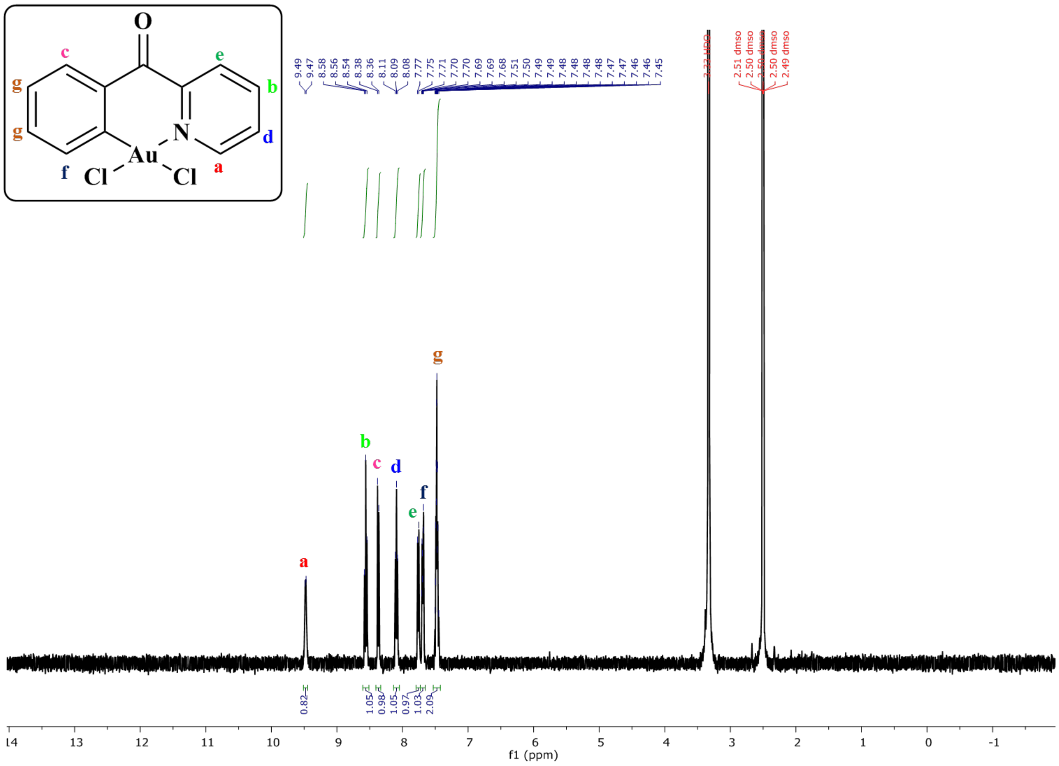

Figure S23. ^{1}H NMR spectrum of complex **7** in DMSO-d_6 at 298K.

Supplementary figures

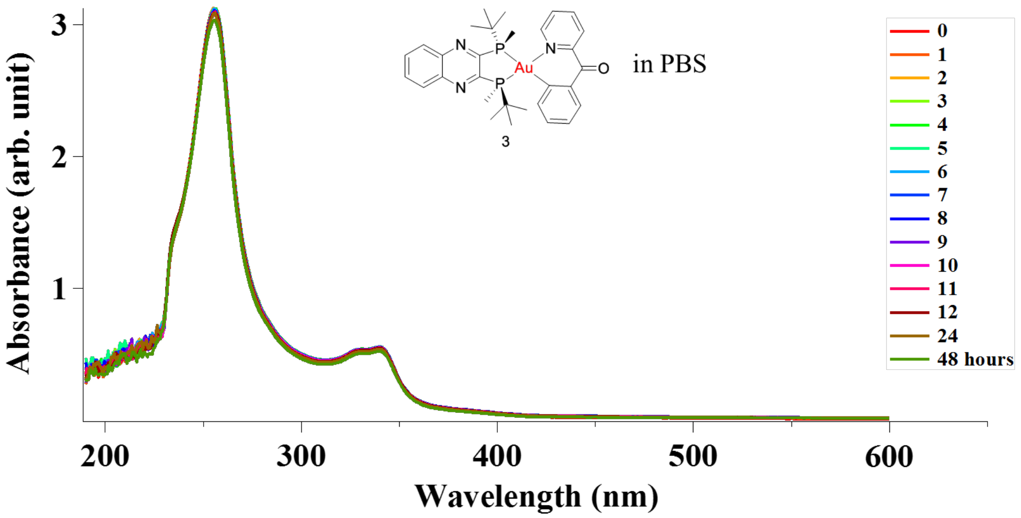

Figure S24. UV-Vis absorption spectrum of **3** in PBS. Concentration of complex **3** = 50 μM. DMSO was used for stock solution.

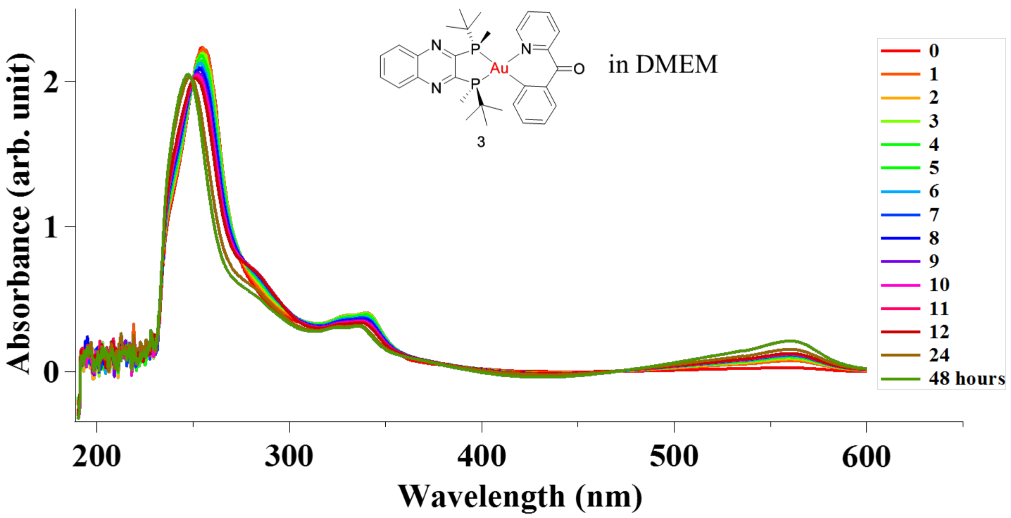

Figure S25. UV-Vis absorption spectrum of **3** in DMEM. Concentration of complex **3** = 50 μM. DMSO was used for stock solution.

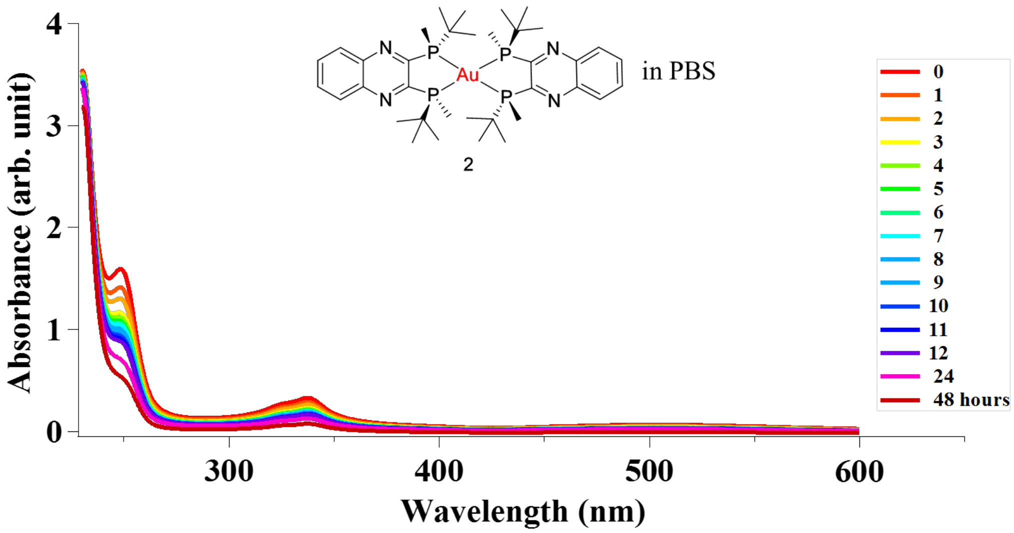

Figure S26. UV-Vis absorption spectrum of **2** in PBS. Concentration of complex **2** = 50 μM. DMSO was used for stock solution.

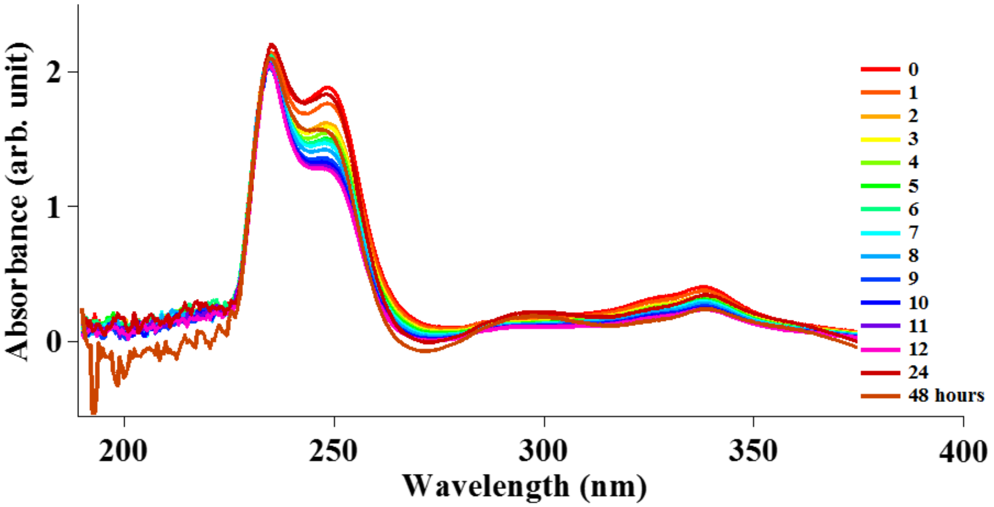

Figure S27. UV-Vis absorption spectrum of **2** in DMEM. Concentration of complex **2** = 50 μM. DMSO was used for stock solution.

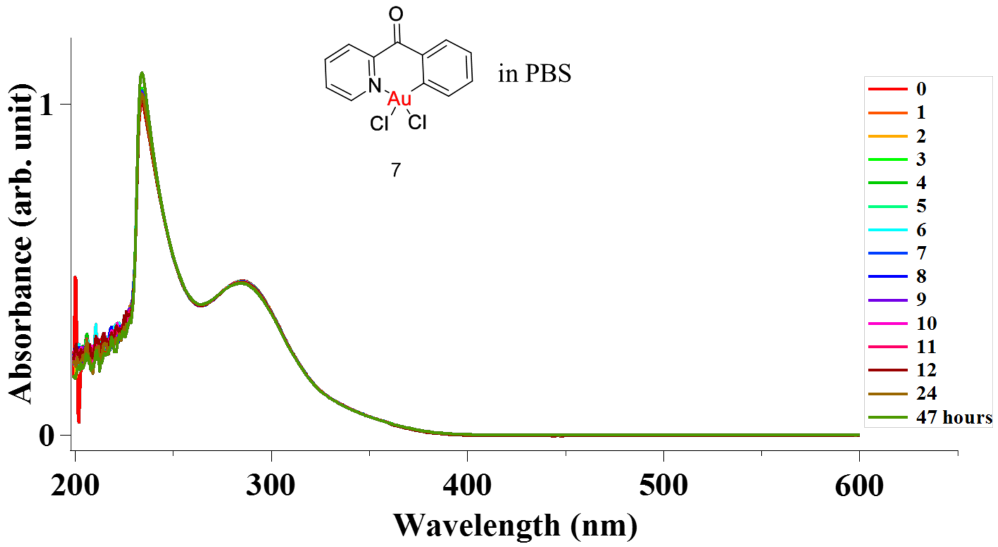

Figure S28. UV-Vis absorption spectra of **7** in PBS. Concentration of complex **7** = 50 μM. DMSO was used for stock solution.

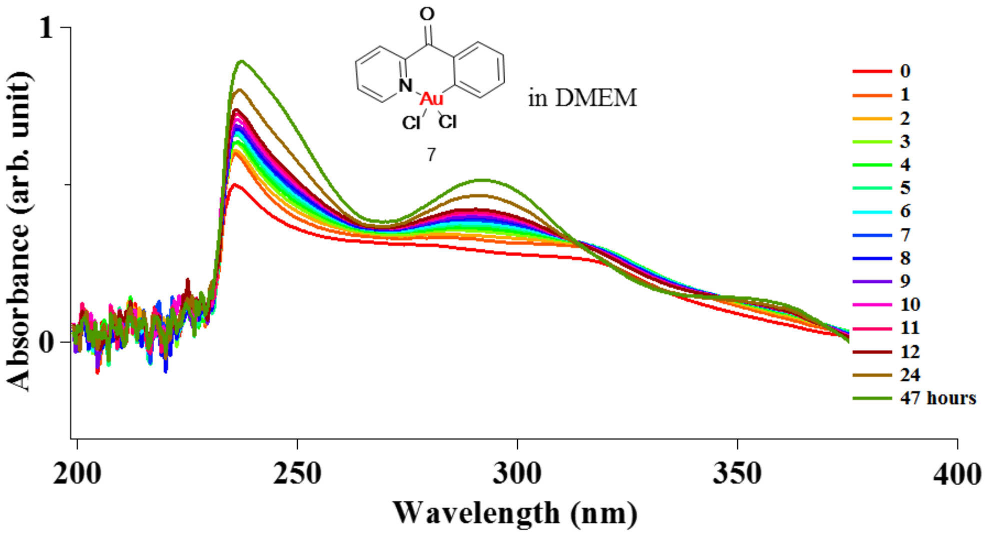

Figure S29. UV-Vis absorption spectra of **7** in DMEM. Concentration of complex **7** = 50 μM. DMSO was used for stock solution.

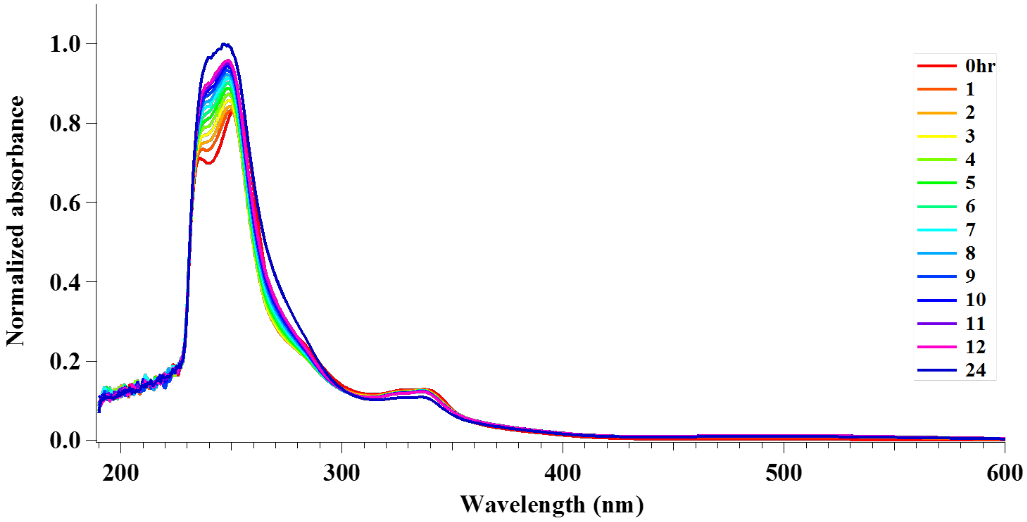

Figure S30. UV-Vis absorption spectra of **3** (25 μM) with L-glutathione (10 mM) in PBS. DMSO was used for stock solution of **3**.

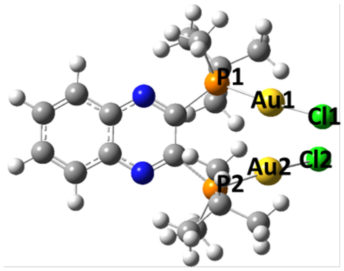

Complex		length	(calc)	angle	(calc)	% error
1	P1-Au1	2.235	2.302			3.0
	P2-Au2	2.225	2.302			3.5
	Au1-Cl1	2.290	2.334			1.9
	Au1-Cl2	2.284	2.334			2.2
	∠P1-Au1-Cl1			170.96	171.44	0.3
	∠P2-Au2-Cl2			171.77	171.40	0.2

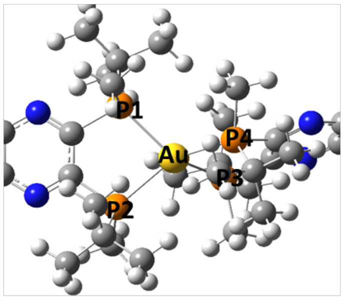

Complex		length	(calc)	angle	(calc)	% error
2	P1-Au	2.396	2.506			4.6
	P2-Au	2.390	2.502			4.7
	P3-Au	2.389	2.506			4.9
	P4-Au	2.393	2.502			4.6
	∠P1-Au-P2			87.79	86.13	1.9
	∠P1-Au-P3			137.50	132.65	3.5
	∠P1-Au-P4			106.48	111.58	4.8

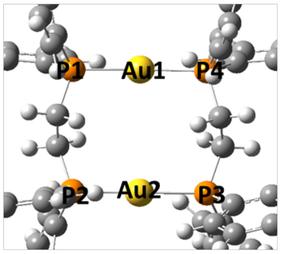

Complex		length	(calc)	angle	(calc)	% error
4	P1-Au1	2.303	2.378			3.3
	P2-Au2	2.315	2.379			2.8
	P3-Au2	2.313	2.379			2.9
	P4-Au1	2.310	2.378			2.9
	∠P1-Au1-P4			166.80	169.07	1.4
	∠P2-Au1-P3			177.82	170.98	3.8

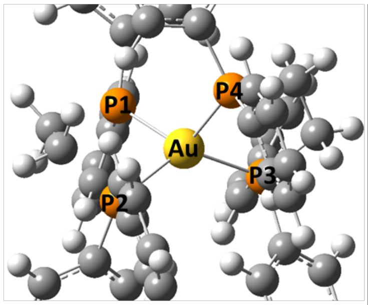

Complex		length	(calc)	angle	(calc)	% error
5	P1-Au	2.411	2.347			2.7
	P2-Au	2.392	2.361			1.3
	P3-Au	2.433	2.347			3.5
	P4-Au	2.387	2.362			1.0
	∠P1-Au-P2			85.97	91.13	6.0
	∠P1-Au-P3			116.67	128.50	10.1
	∠P1-Au-P4			118.46	114.37	3.5

Figure S31. Geometries comparison of 1, 2, 4, and 5, with measured and calculated values by selecting representative parts of gold complexes. The unit of length is Å and that of angle is degree. All calculation were done with B3LYP, SDD for Au and 6-31G(d,p) for others.

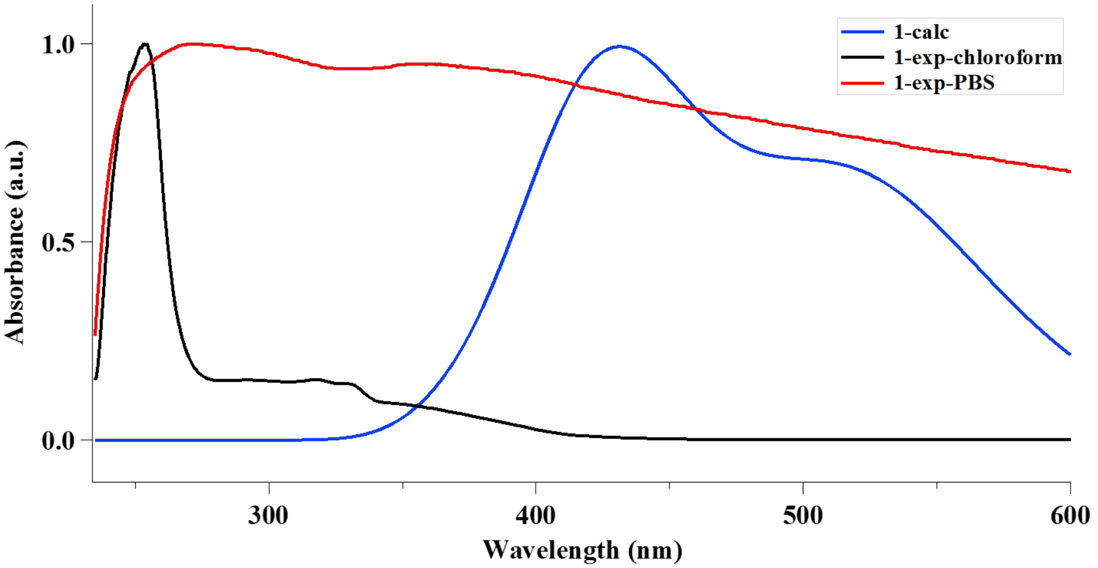

Complex	Peak (measured, nm)	Peak (calculated, nm)	Transition (MO#)		
			from	to	
1	254	432	124	127	MLCT
	318	522	126	127	MLCT
	330				

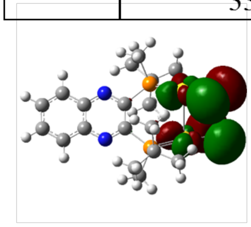

124 (HOMO-2)

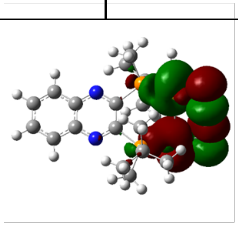

126 (HOMO)

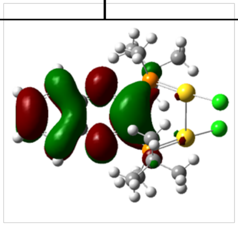

127 (LUMO)

Figure S32. UV-Vis absorption spectrum of **1**, in PBS (red) and in $CHCl_3$ (black) at the concentration of 50 µM. DMSO was used for stock solution. And the blue spectrum is theoretical (TD-DFT calculation). In PBS, complex **1** was aggregated and showed absorption in all over the range.

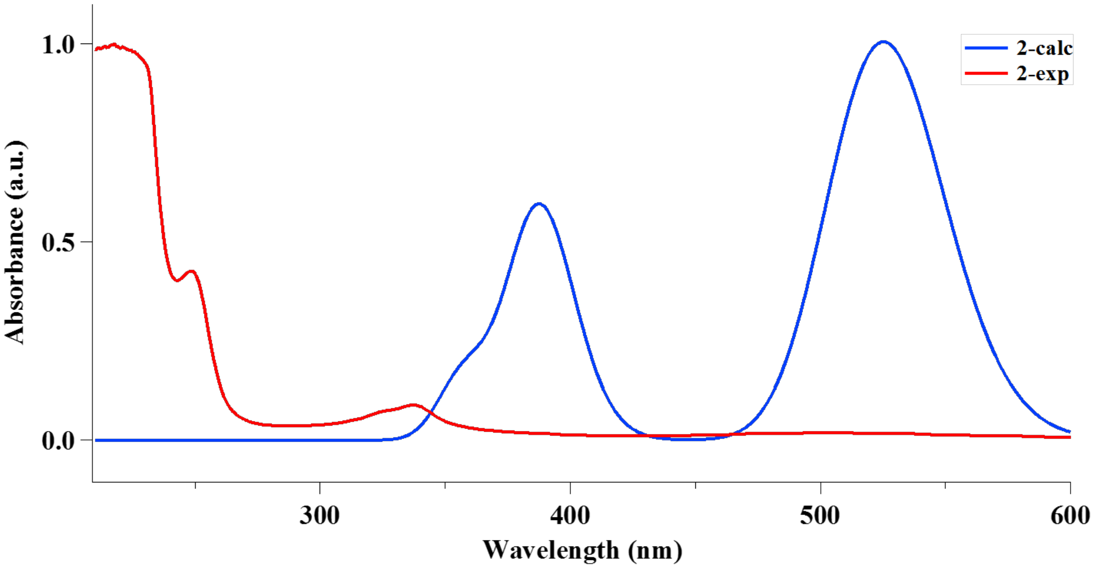

Complex	Peak (measured, nm)	Peak (calculated, nm)	Transition (MO#)		
			from	to	
2	221	359	189	192	MLCT
	249	387	188	191	MLCT
	326	525	189	190	MLCT
	337				

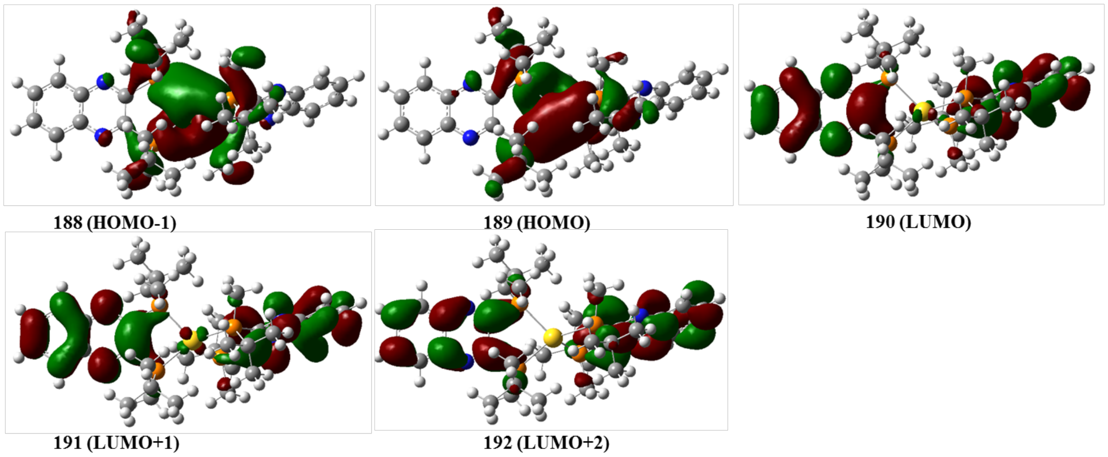

Figure S33. UV-Vis absorption spectrum of **2**, in PBS (red) at the concentration of 50 μM. DMSO was used for stock solution. And the blue is theoretical spectrum (TD-DFT calculation).

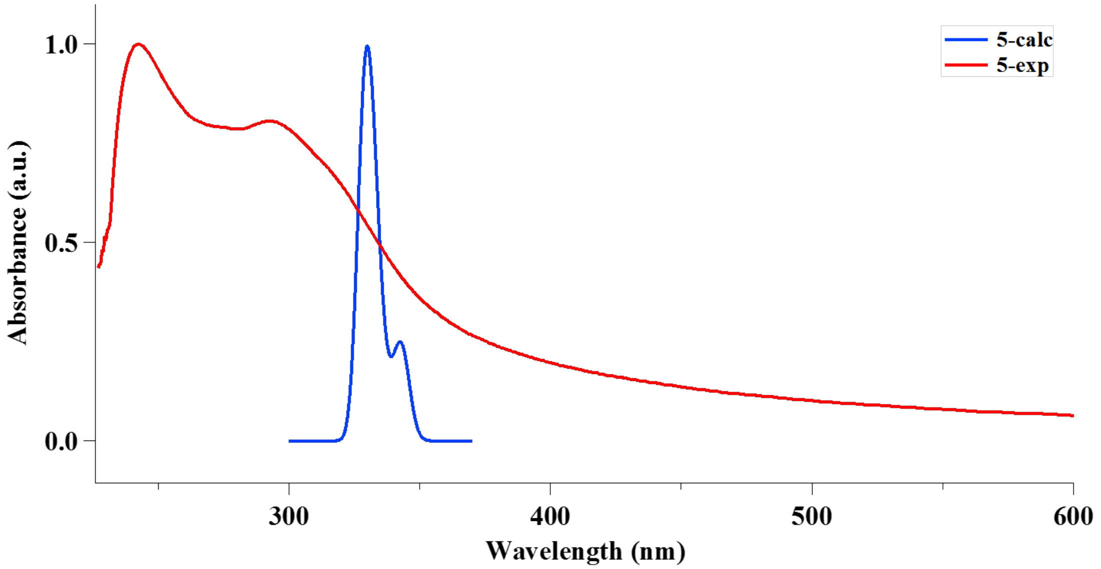

Complex	Peak (measured, nm)	Peak (calculated, nm)	Transition (MO#)		
			from	to	
5	243	330	218	221	MLCT
			219	221	
			219	223	
	292	343	219	220	MLCT
			219	221	
			219	222	

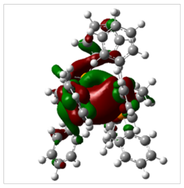
218 (HOMO-1)
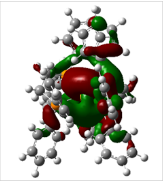
219 (HOMO)
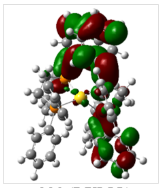
220 (LUMO)
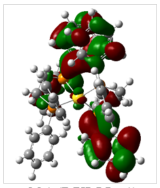
221 (LUMO+1)
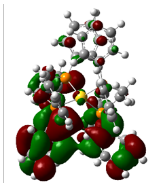
222 (LUMO+2)
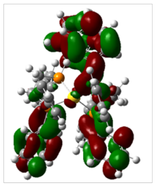
223 (LUMO+3)

Figure S34. UV-Vis absorption spectrum of **5**, in PBS (red) at the concentration of 50 μM. DMSO was used for stock solution. And the blue is theoretical spectrum (TD-DFT calculation).

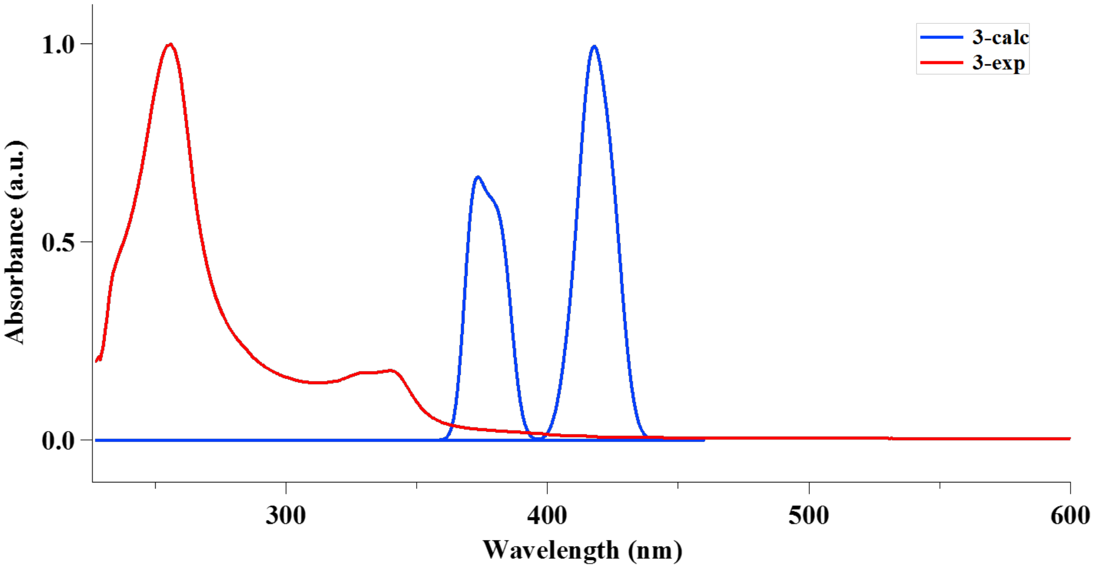

Complex	Peak (measured, nm)	Peak (calculated, nm)	Transition (MO#)		
			from	to	
3	256	371	143	147	LMCT
			143	148	
			146	148	
			146	149	
	328	417	142	147	LMCT
			144	147	
			145	147	
	340	424	144	147	LMCT
			145	147	

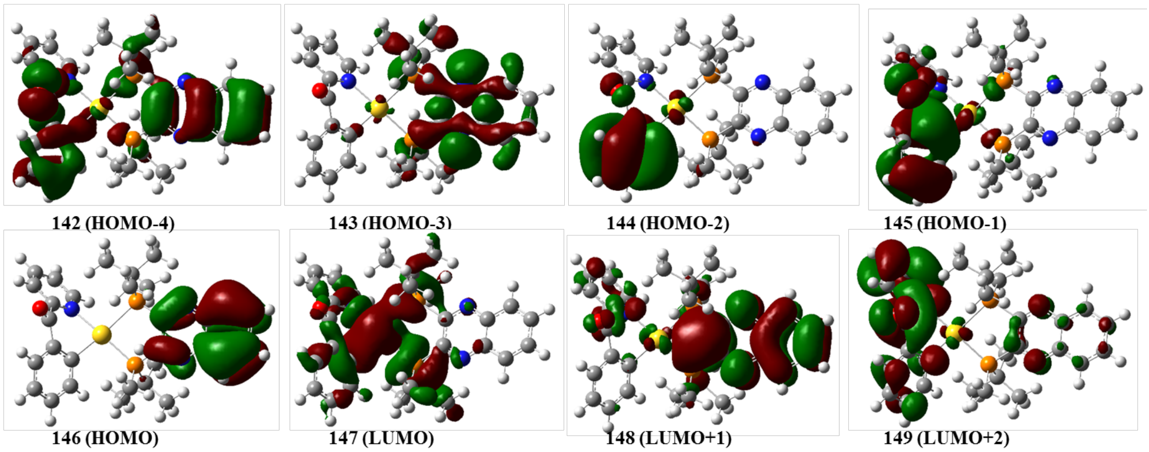

Figure S35. UV-Vis absorption spectrum of **3**, in PBS (red) at the concentration of 50 μM. DMSO was used for stock solution. And the blue is theoretical spectrum (TD-DFT calculation).

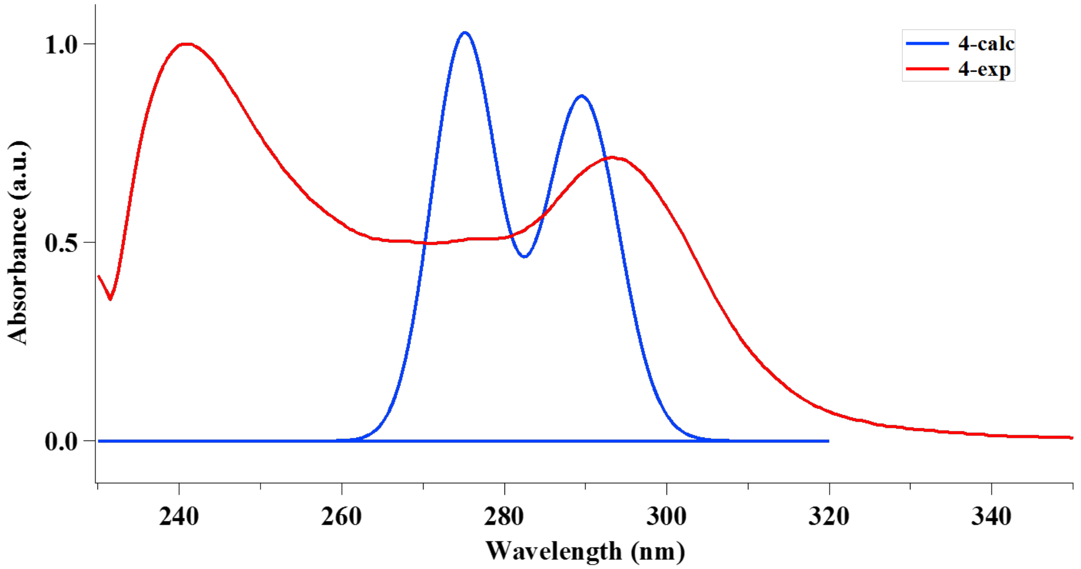

Complex	Peak (measured, nm)	Peak (calculated, nm)	Transition (MO#)		
			from	to	
4	241	276	225	229	LMCT
			226	229	
	294	290	228	229	LMCT

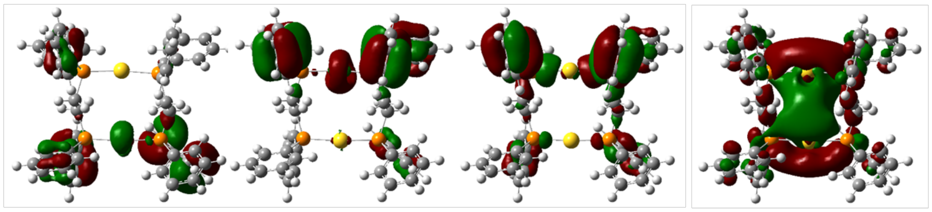

225 (HOMO-3) 226 (HOMO-2) 228 (HOMO) 229 (LUMO)

Figure S36. UV-Vis absorption spectrum of **4**, in PBS (red) at the concentration of 50 μM. DMSO was used for stock solution. And the blue is theoretical spectrum (TD-DFT calculation).

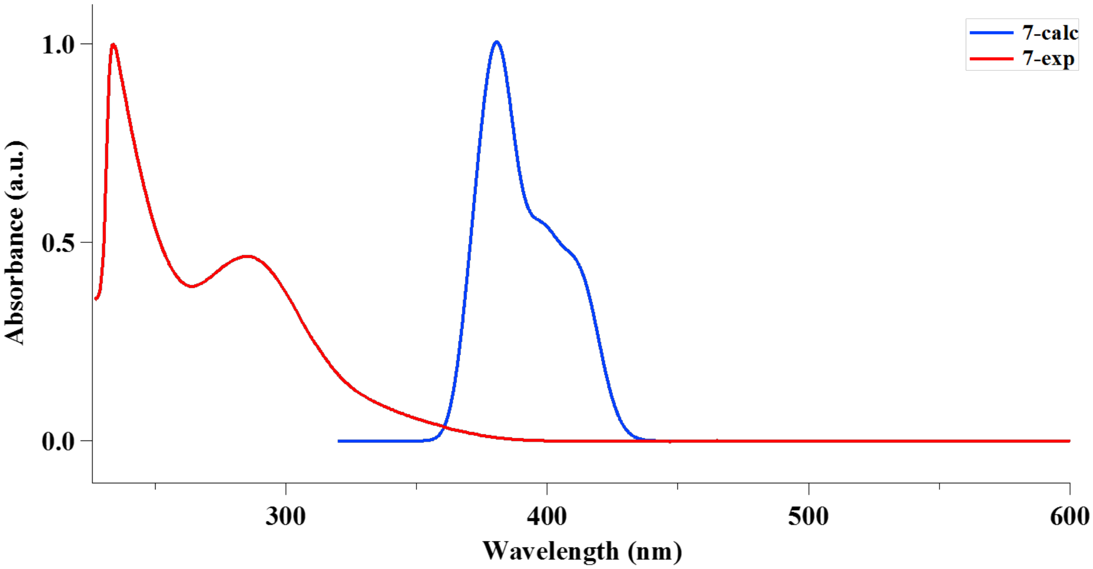

<table>
<tr><th rowspan="2">Complex</th><th rowspan="2">Peak (measured, nm)</th><th rowspan="2">Peak (calculated, nm)</th><th colspan="2">Transition (MO#)</th><th rowspan="2"></th></tr>
<tr><th>from</th><th>to</th></tr>
<tr><td rowspan="5">7</td><td rowspan="3">234</td><td rowspan="3">381</td><td>67</td><td>76</td><td rowspan="3">LMCT</td></tr>
<tr><td>70</td><td>76</td></tr>
<tr><td>72</td><td>76</td></tr>
<tr><td rowspan="2">285</td><td rowspan="2">398</td><td>68</td><td>75</td><td rowspan="2">LLCT</td></tr>
<tr><td>74</td><td>75</td></tr>
</table>

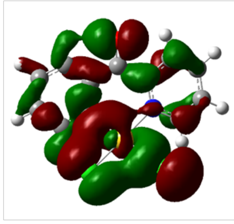
67 (HOMO-7)

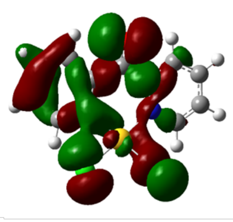
68 (HOMO-6)

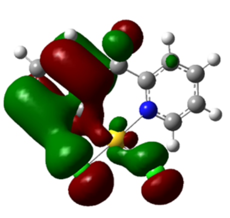
70 (HOMO-4)

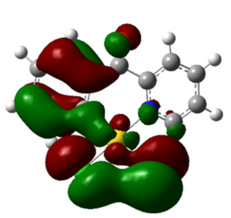
72 (HOMO-2)

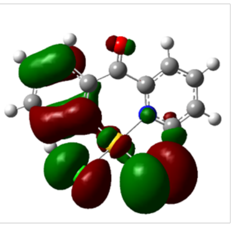
74 (HOMO)

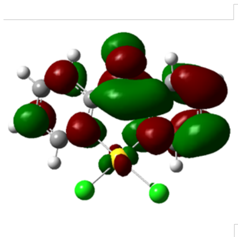
75 (LUMO)

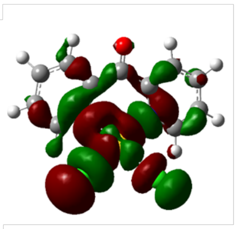
76 (LUMO+1)

Figure S37. UV-Vis absorption spectrum of **7**, in PBS (red) at the concentration of 50 μM. DMSO was used for stock solution. And the blue is theoretical spectrum (TD-DFT calculation).

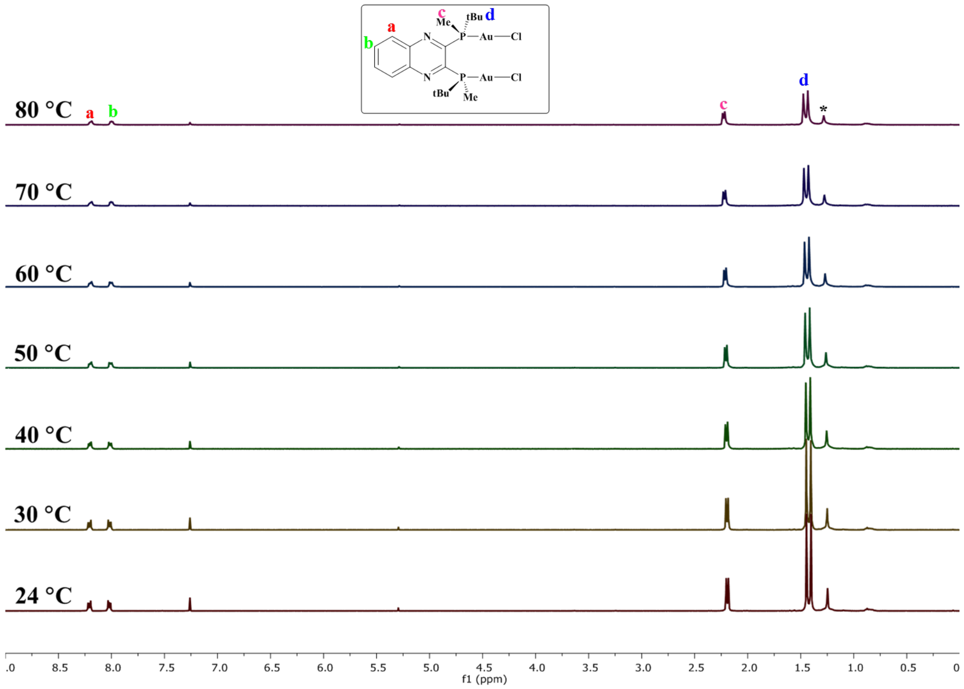

Figure S38. The variable temperature ^{1}H-NMR of complex **1** in $CDCl_3$ at the temperature range 24-80 °C. * Grease

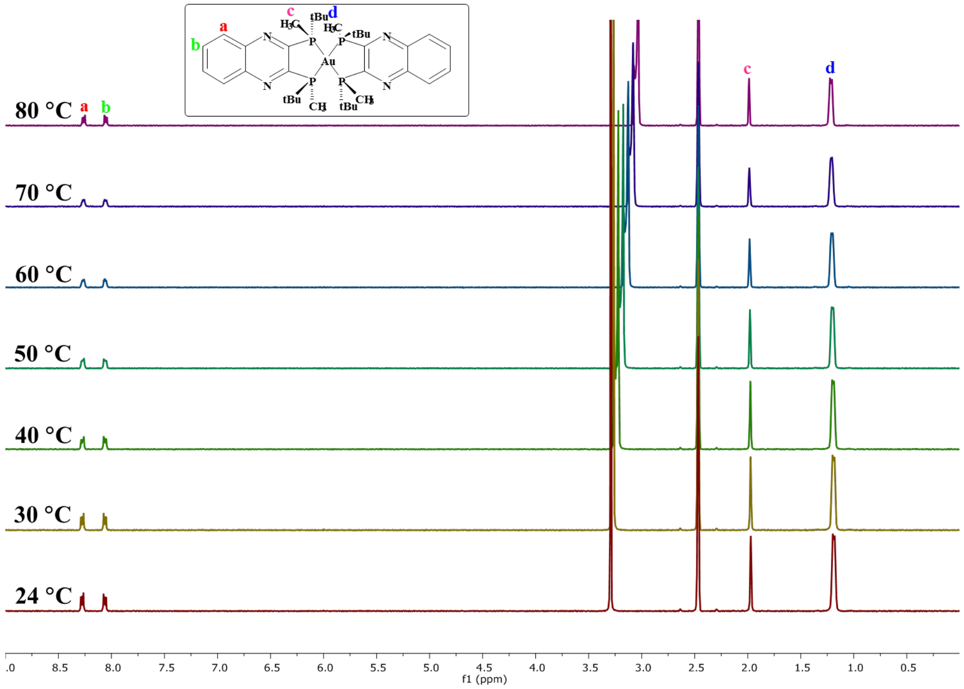

Figure S39. The variable temperature ^{1}H-NMR of complex **2** in DMSO-d_6 at the temperature range 23-80 °C.

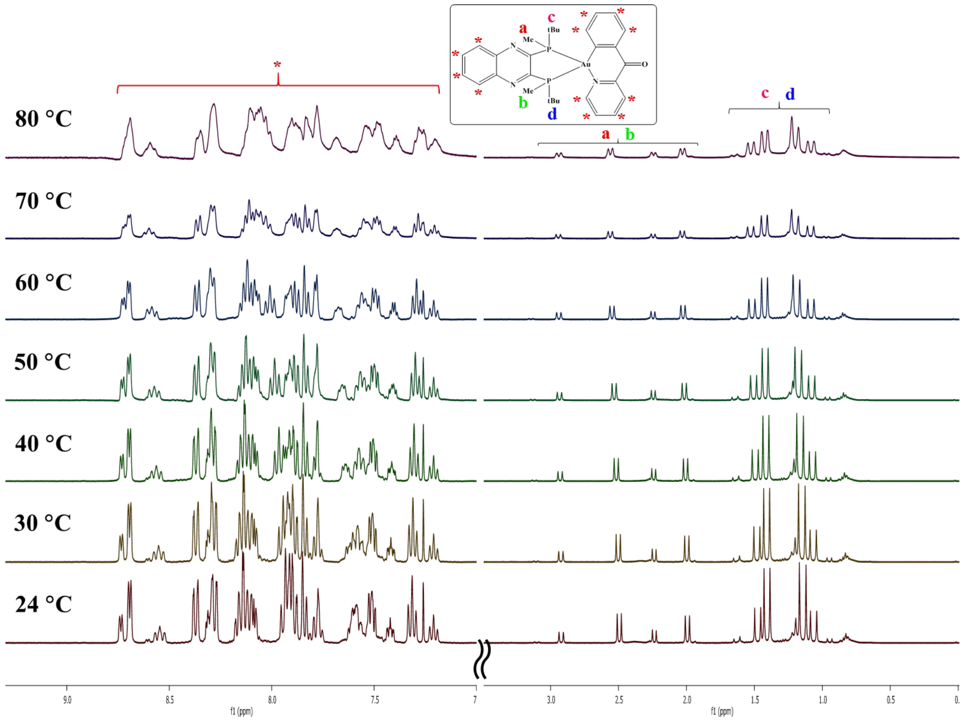

Figure S40. The variable temperature ^{1}H-NMR of complex **3** in $CDCl_3$ at the temperature range 24-80 °C. The different y-axis scales were used to clarify the peaks at 7 – 9 ppm.

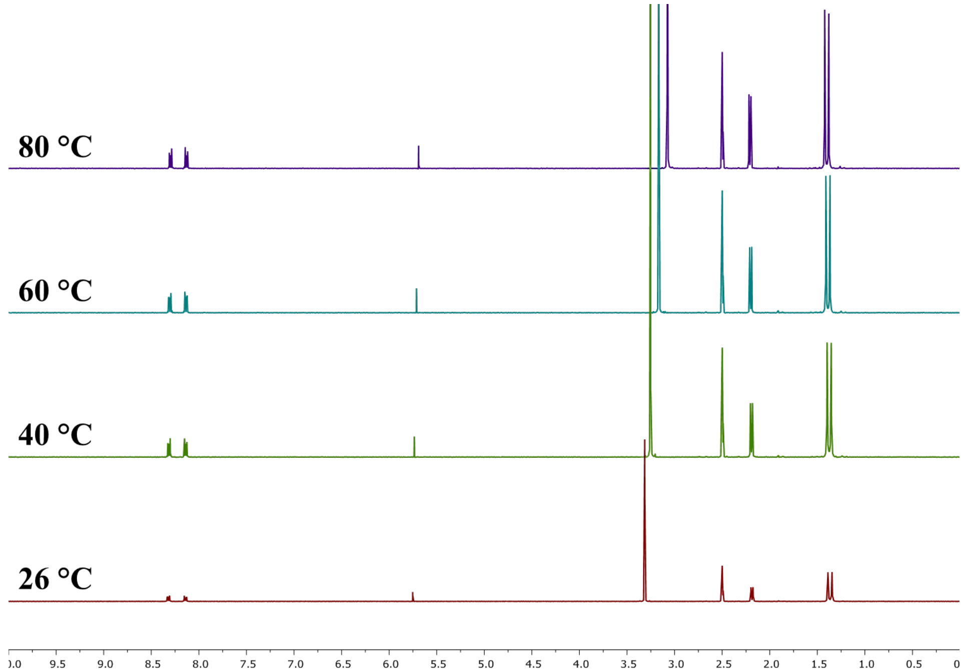

Figure S41. The variable temperature ^{1}H-NMR of complex **1** in DMSO-d_6 at the temperature range 26-80 °C.

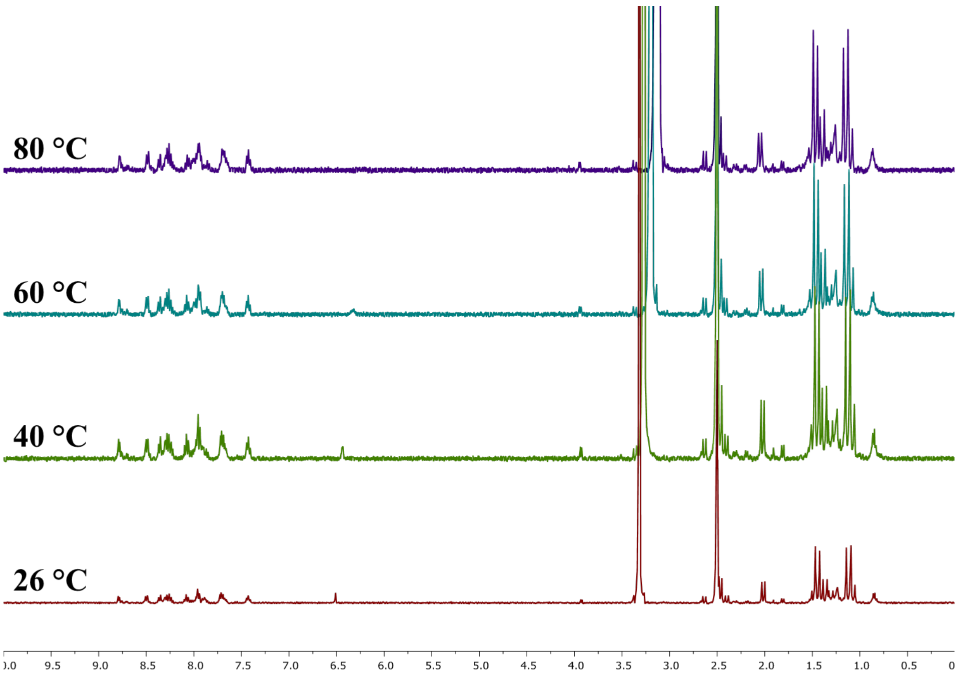

Figure S42. The variable temperature ^{1}H-NMR of complex **3** in DMSO-d_6 at the temperature range 26-80 °C.

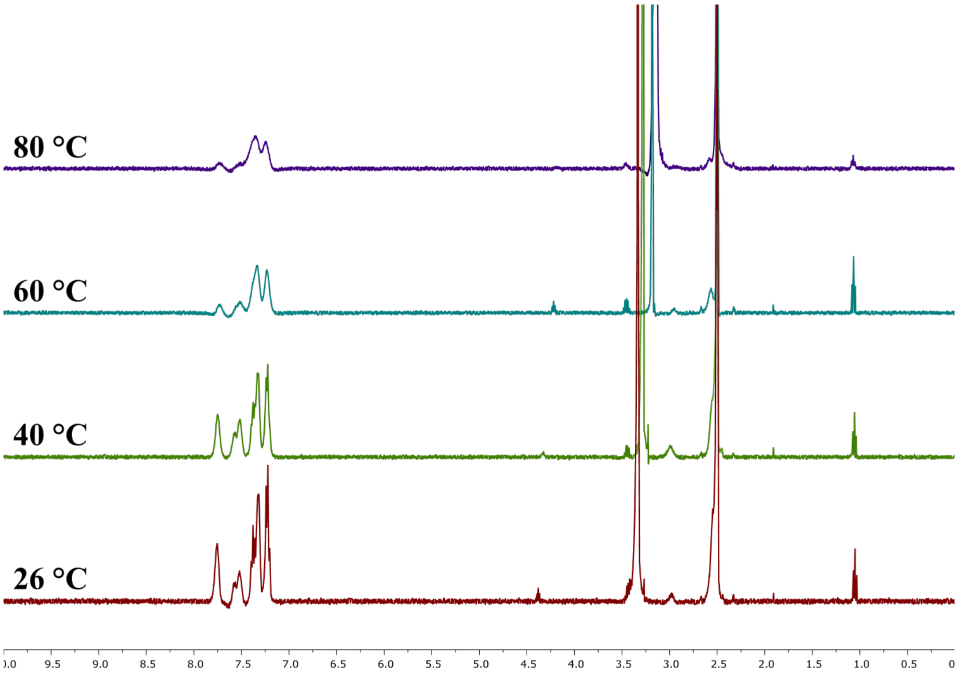

Figure S43. The variable temperature ^{1}H-NMR of complex **4** in DMSO-d_6 at the temperature range 26-80 °C.

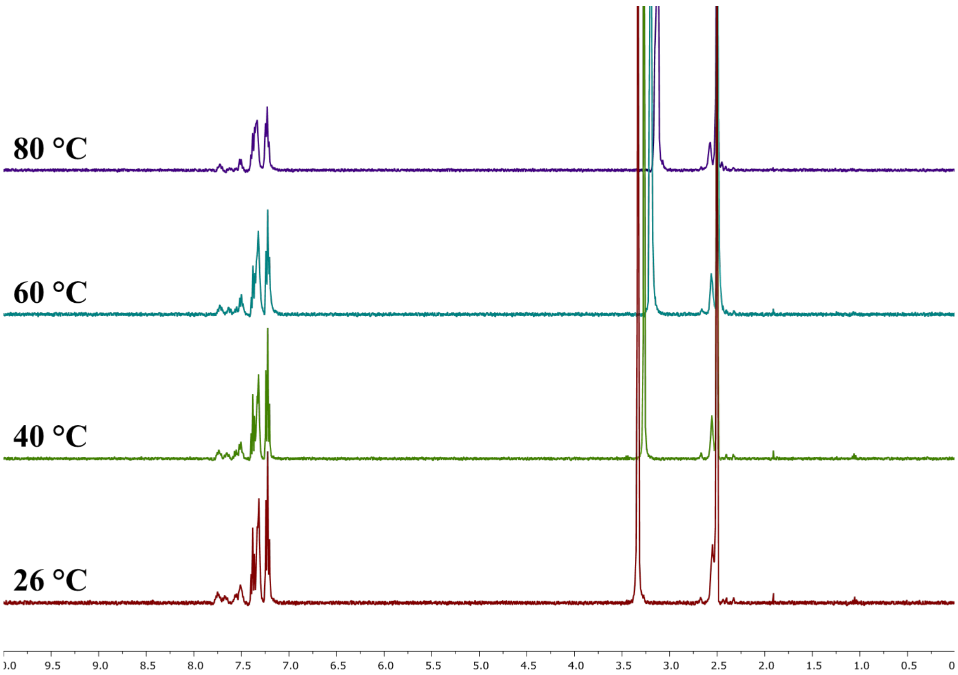

Figure S44. The variable temperature ^{1}H-NMR of complex **5** in DMSO-d_6 at the temperature range 26-80 °C.

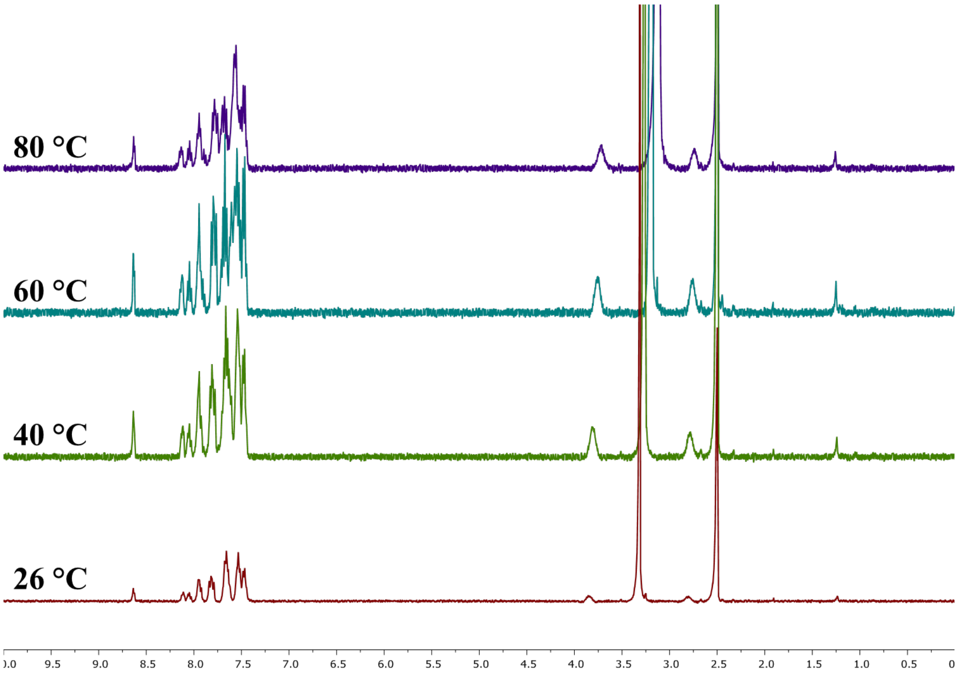

Figure S45. The variable temperature ^{1}H-NMR of complex **6** in DMSO-d_6 at the temperature range 26-80 °C.

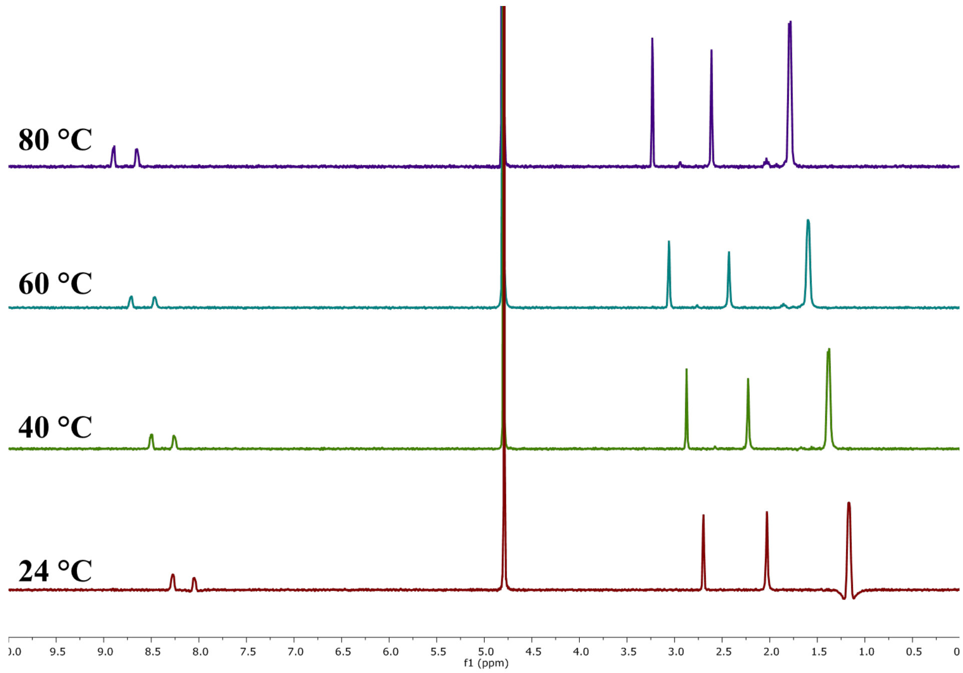

Figure S46. The variable temperature ^{1}H-NMR of complex **2** in D_2O at the temperature range 24-80 °C. DMSO-d_6 was used for stock solution.

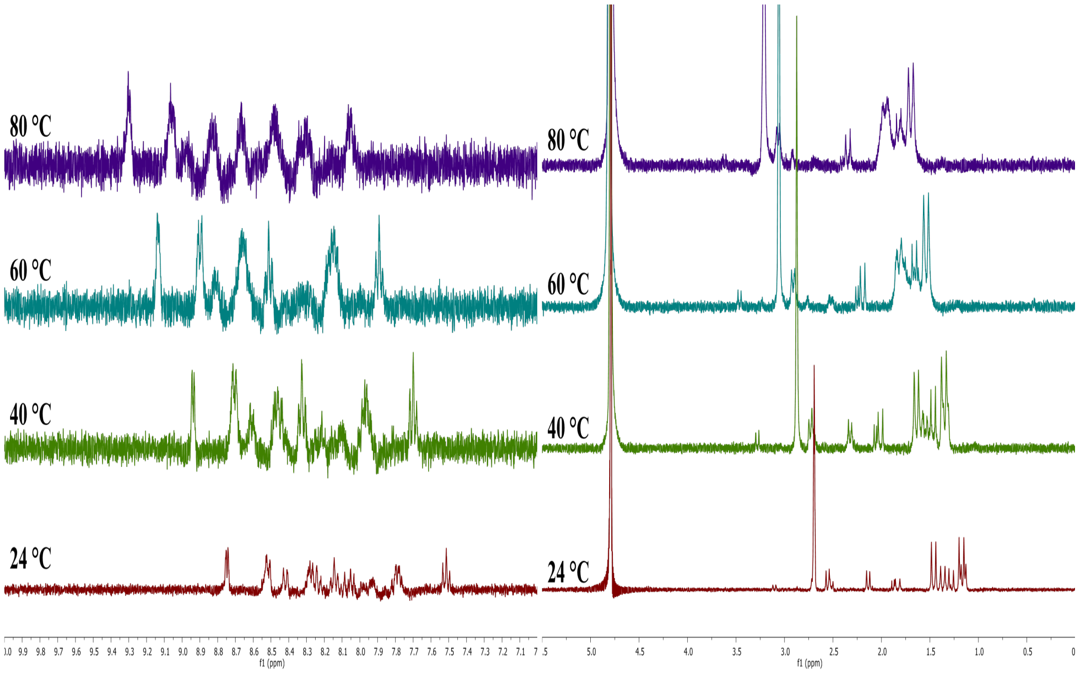

Figure S47. The variable temperature ^{1}H-NMR of complex **3** in D_2O at the temperature range 24-80 °C. DMSO-d_6 was used for stock solution.

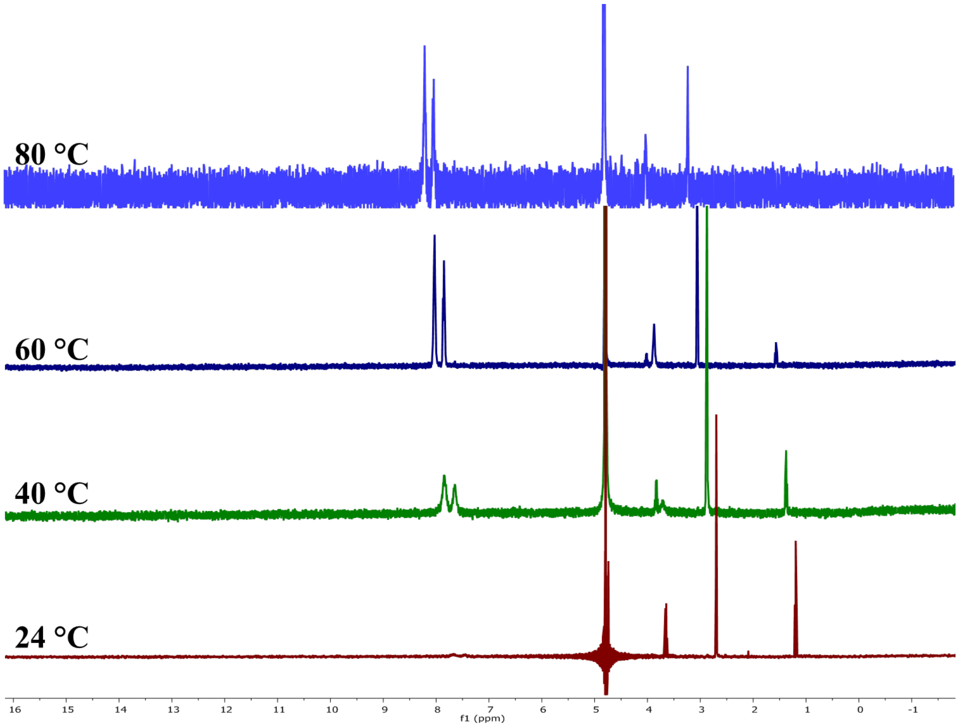

Figure S48. The variable temperature ^{1}H-NMR of complex **4** in D_2O at the temperature range 24-80 °C. DMSO-d_6 was used for stock solution. At 24 °C, solubility is poor. But increasing temp. makes solubility better.

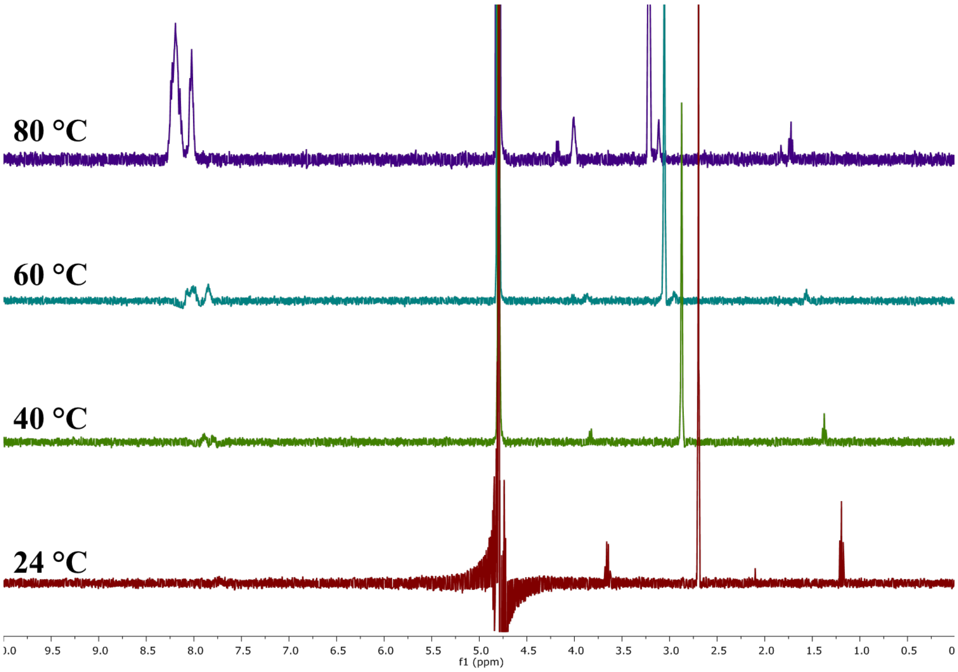

Figure S49. The variable temperature ^{1}H-NMR of complex **5** in D_2O at the temperature range 24-80 °C.

DMSO-d_6 was used for stock solution. At 24 °C, solubility is poor. But increasing temp. makes solubility better.

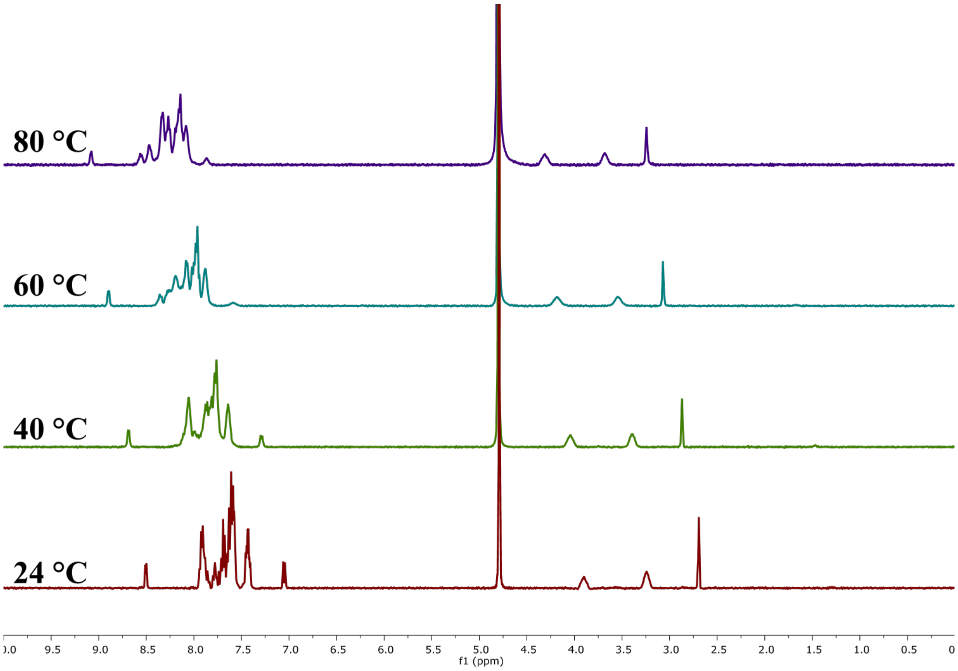

Figure S50. The variable temperature ^{1}H-NMR of complex **6** in D_2O at the temperature range 24-80 °C. DMSO-d_6 was used for stock solution.

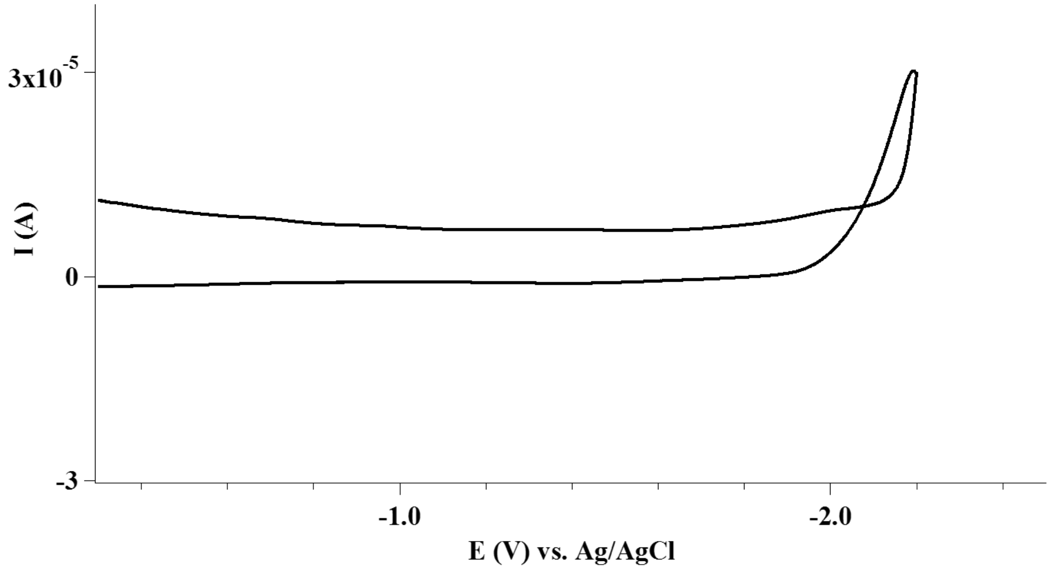

Figure S51. Background cyclic voltammograms recorded at a platinum electrode in DMSO, with $NaClO_4$ supporting electrolyte; scan rate 0.1 V sec^{-1}.

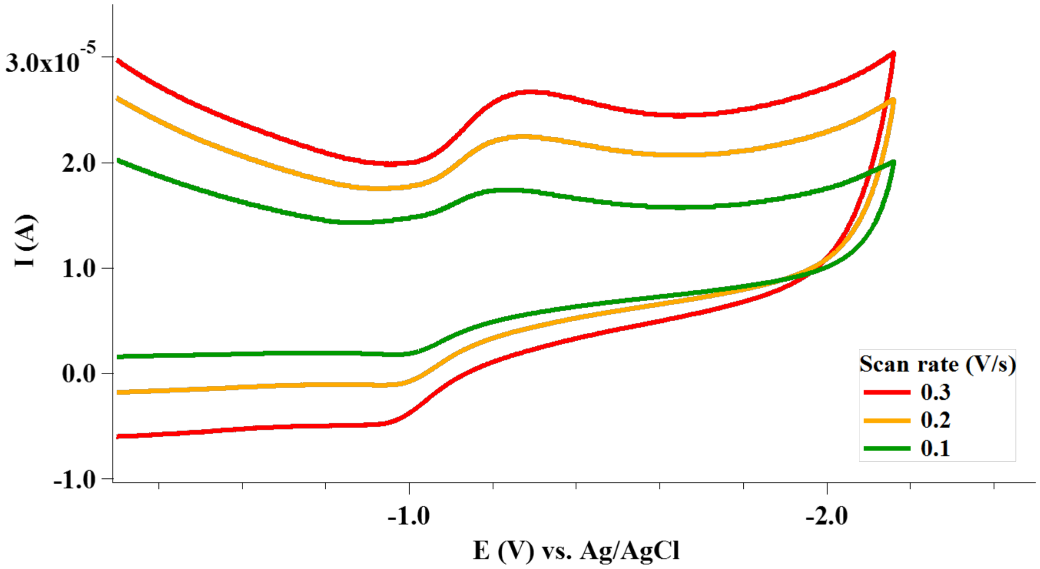

Figure S52. Cyclic voltammograms recorded at a platinum electrode in DMSO solution of 1.0 mM of **1**, with $NaClO_4$ supporting electrolyte; scan rate 0.3, 0.2, and 0.1 V sec^{-1}.

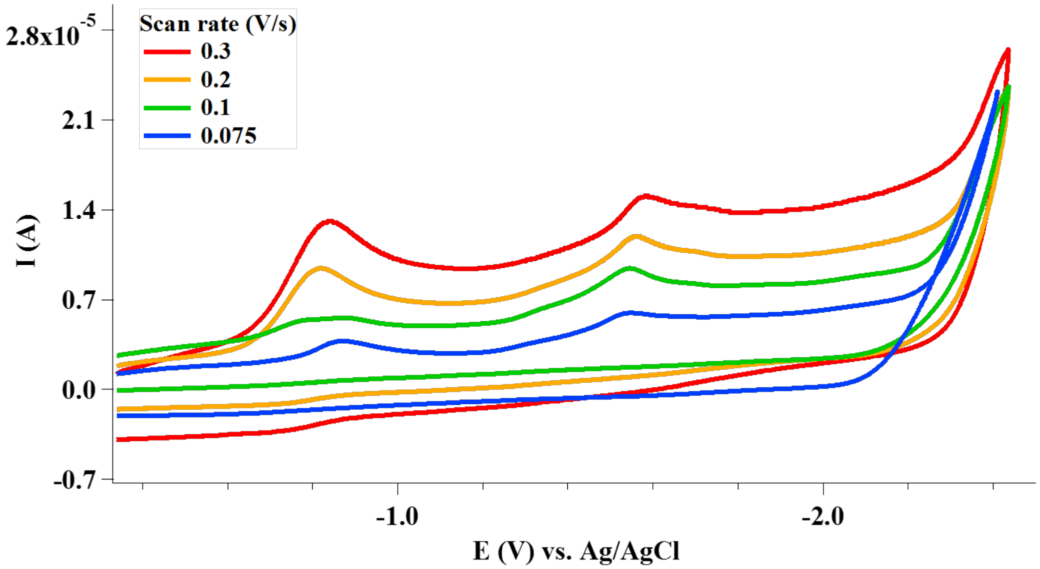

Figure S53. Cyclic voltammograms recorded at a platinum electrode in DMSO solution of 1.0 mM of **4**, with $NaClO_4$ supporting electrolyte; scan rate 0.3, 0.2, 0.1, and 0.075 V sec^{-1}.

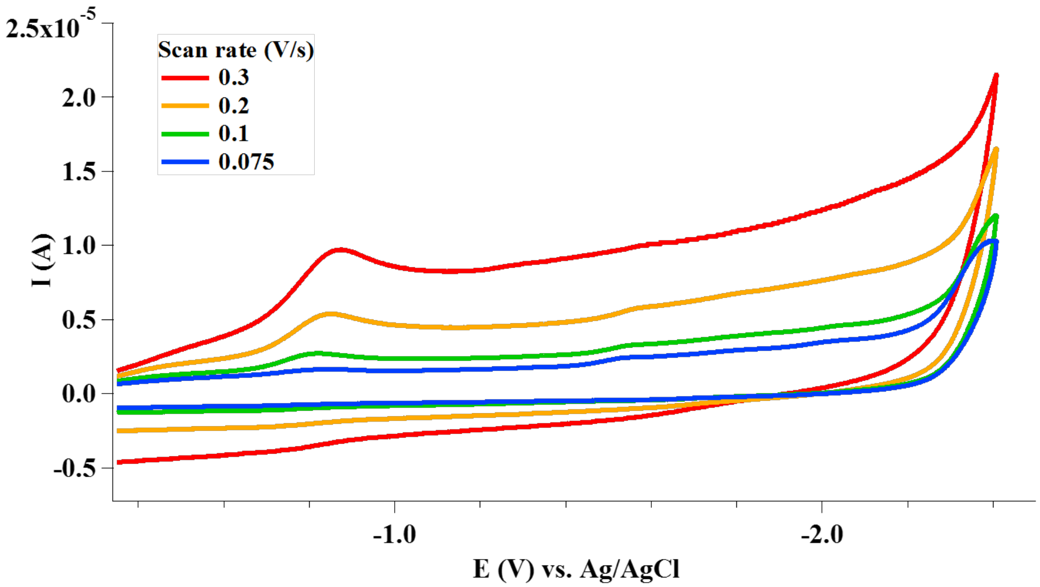

Figure S54. Cyclic voltammograms recorded at a platinum electrode in DMSO solution of 1.0 mM of **5**, with $NaClO_4$ supporting electrolyte; scan rate 0.3, 0.2, 0.1, and 0.075 V sec^{-1}.

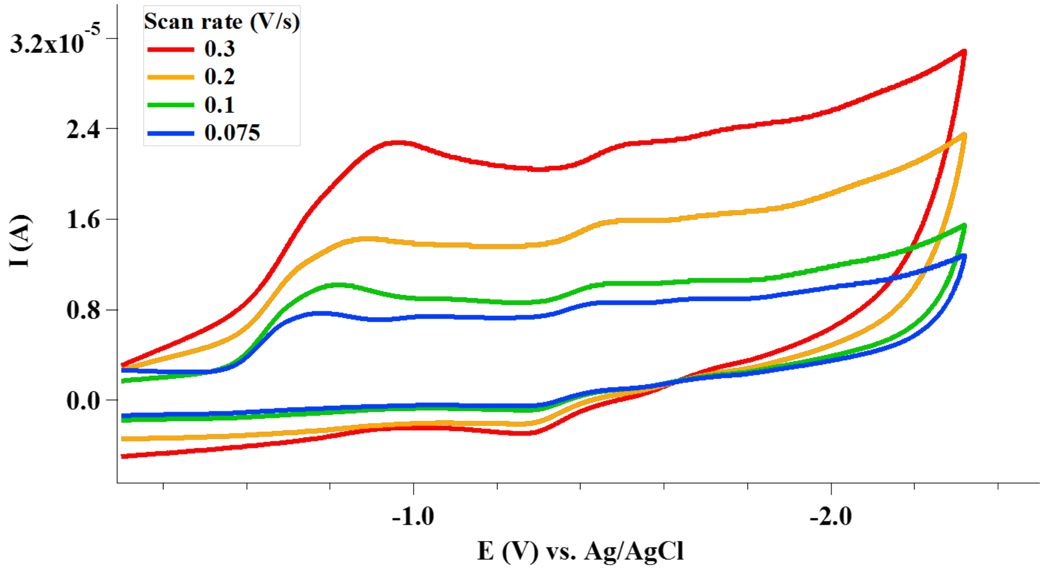

Figure S55. Cyclic voltammograms recorded at a platinum electrode in DMSO solution of 1.0 mM of **3**, with $NaClO_4$ supporting electrolyte; scan rate 0.3, 0.2, 0.1, and 0.075 V sec^{-1}.

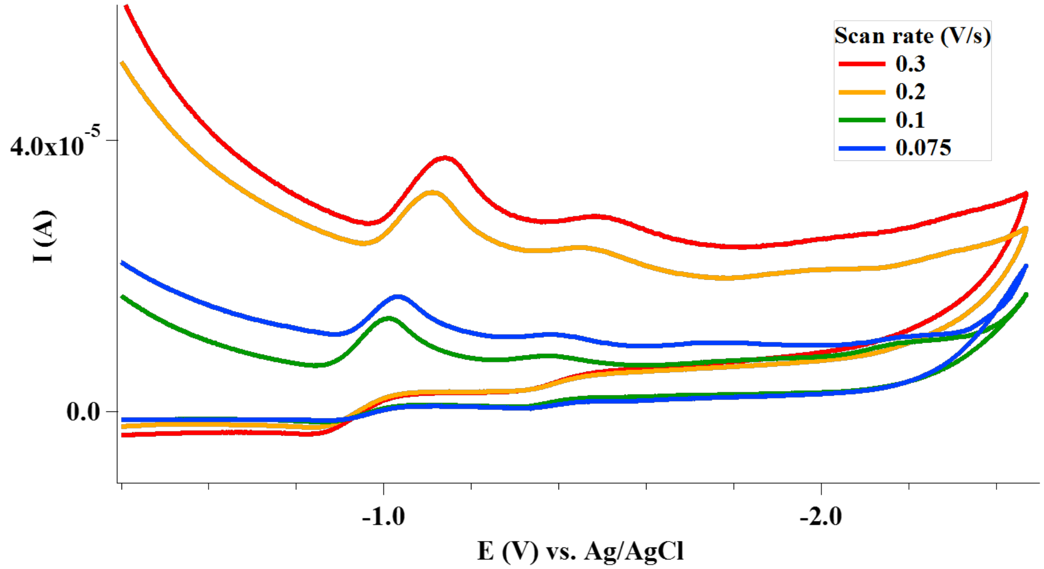

Figure S56. Cyclic voltammograms recorded at a platinum electrode in DMSO solution of 1.0 mM of **6**, with $NaClO_4$ supporting electrolyte; scan rate 0.3, 0.2, 0.1, and 0.075 V sec^{-1}.

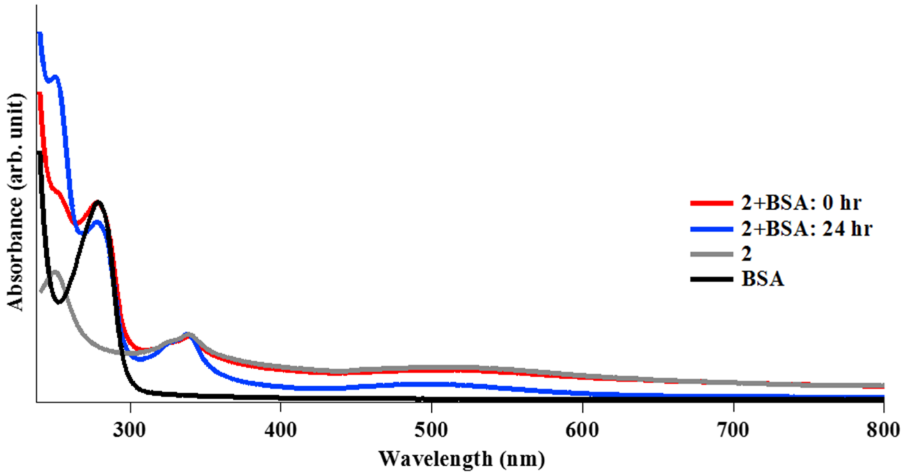

Figure S57. UV-Vis absorption spectra of **2**, BSA, and **2** and BSA mixture in PBS. Concentration of complex 2 and BSA = 25 μM. DMSO was used for stock solution of **2**.

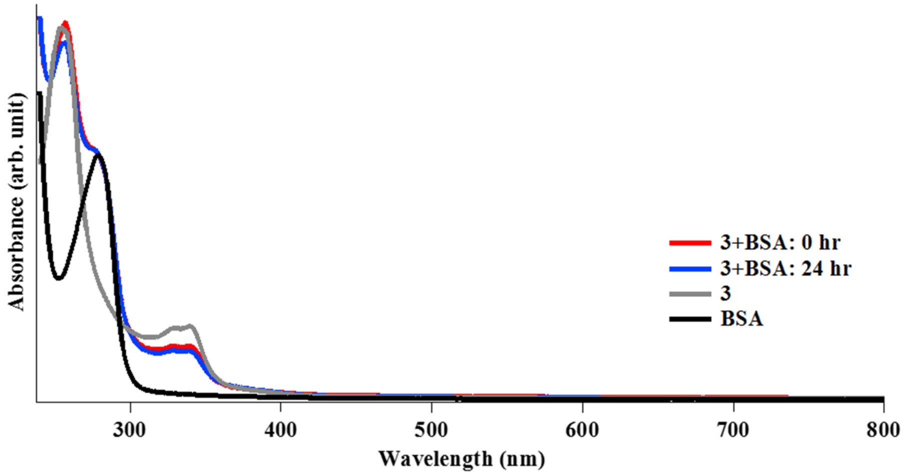

Figure S58. UV-Vis absorption spectra of **3**, BSA, and 3 and BSA mixture in PBS. Concentration of complex **3** and BSA = 25 μM. DMSO was used for stock solution of **3**.

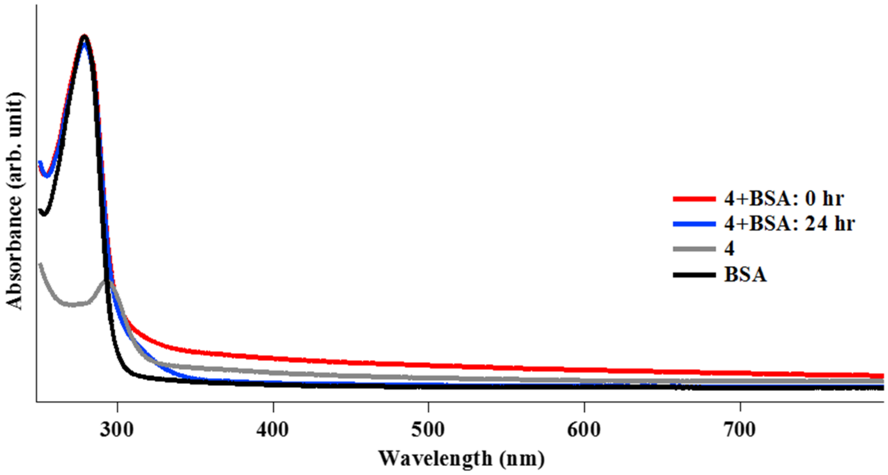

Figure S59. UV-Vis absorption spectra of **4**, BSA, and **4** and BSA mixture in PBS. Concentration of complex **4** and BSA = 25 μM. DMSO was used for stock solution of **4**.

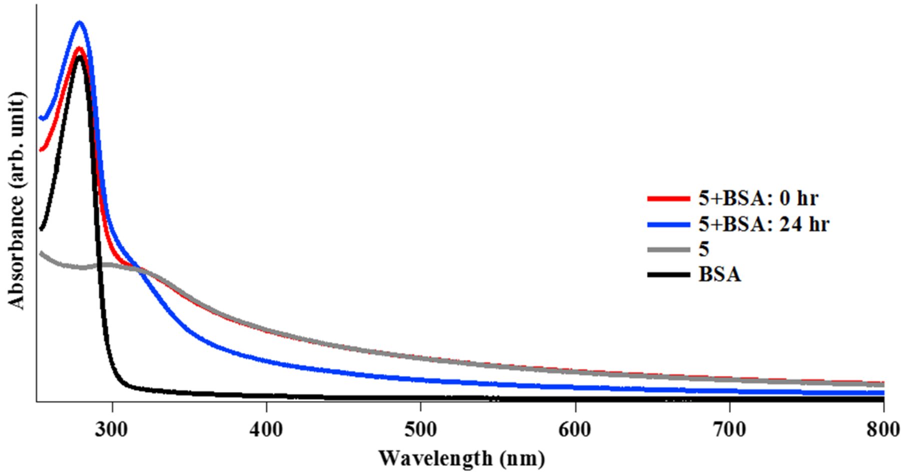

Figure S60. UV-Vis absorption spectra of **5**, BSA, and **5** and BSA mixture in PBS. Concentration of complex **5** and BSA = 25 μM. DMSO was used for stock solution of **5**.

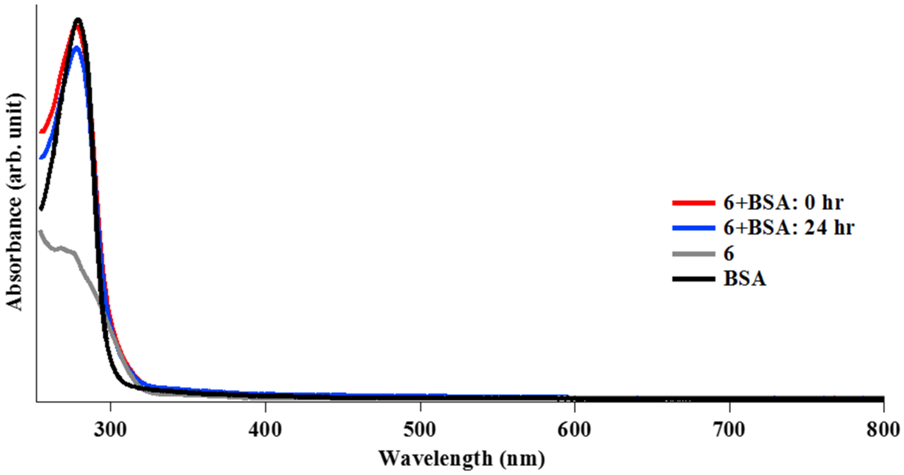

Figure S61. UV-Vis absorption spectra of **6**, BSA, and **6** and BSA mixture in PBS. Concentration of complex **6** and BSA = 25 μM. DMSO was used for stock solution of **6**.

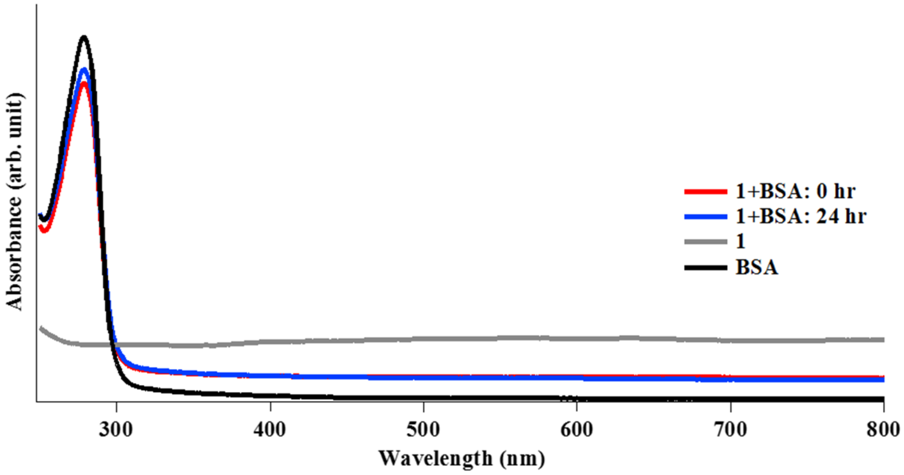

Figure S62. UV-Vis absorption spectra of **1**, BSA, and **1** and BSA mixture in PBS. Concentration of complex **1** and BSA = 25 μM. DMSO was used for stock solution of **1**.

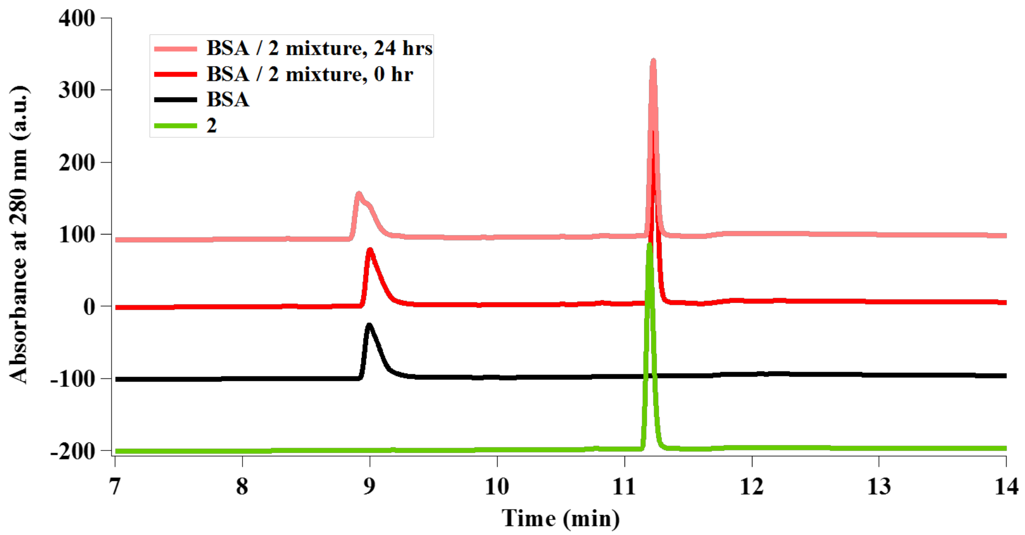

Figure S63. HPLC traces of 2, BSA, and 2+BSA mixture in 24 hours. The concentration of 2 was 1 mg/mL and BSA was 6.7 mg/mL. The 2+BSA solution was 1:1 volume ratio of 2 and BSA.

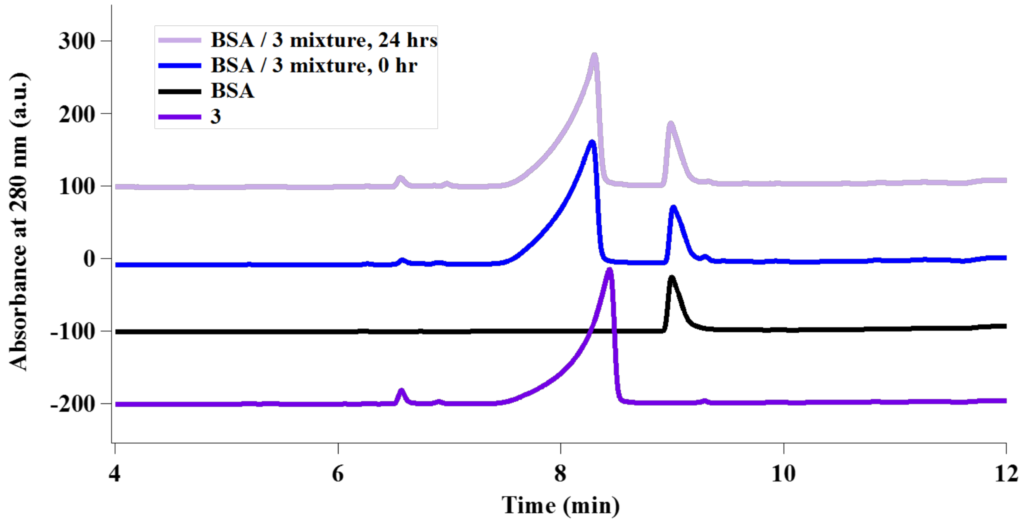

Figure S64. HPLC traces of 3, BSA, and 3+BSA mixture in 24 hours. The concentration of 3 was 1 mg/mL and BSA was 6.7 mg/mL. The 3+BSA solution was 1:1 volume ratio of 3 and BSA.

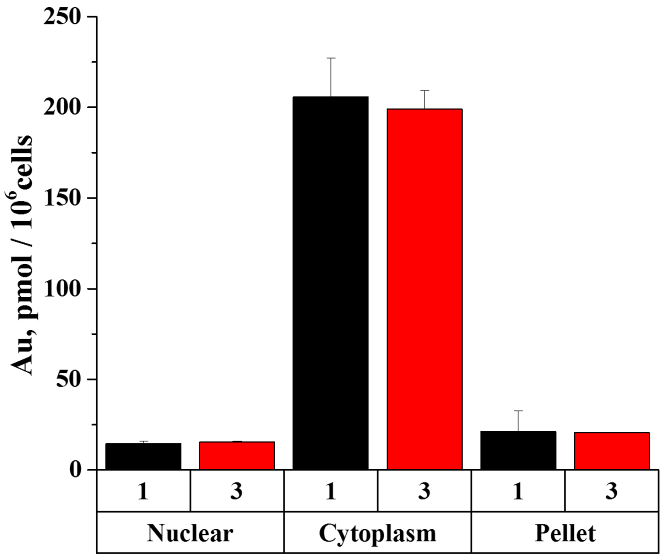

Figure S65. Subcellular localization of Au complexes **1** and **3** in OVCAR8 cells. Cells were treated with 5 μM of compound for 15 h and nuclear and cytoplasmic fractions were isolated.

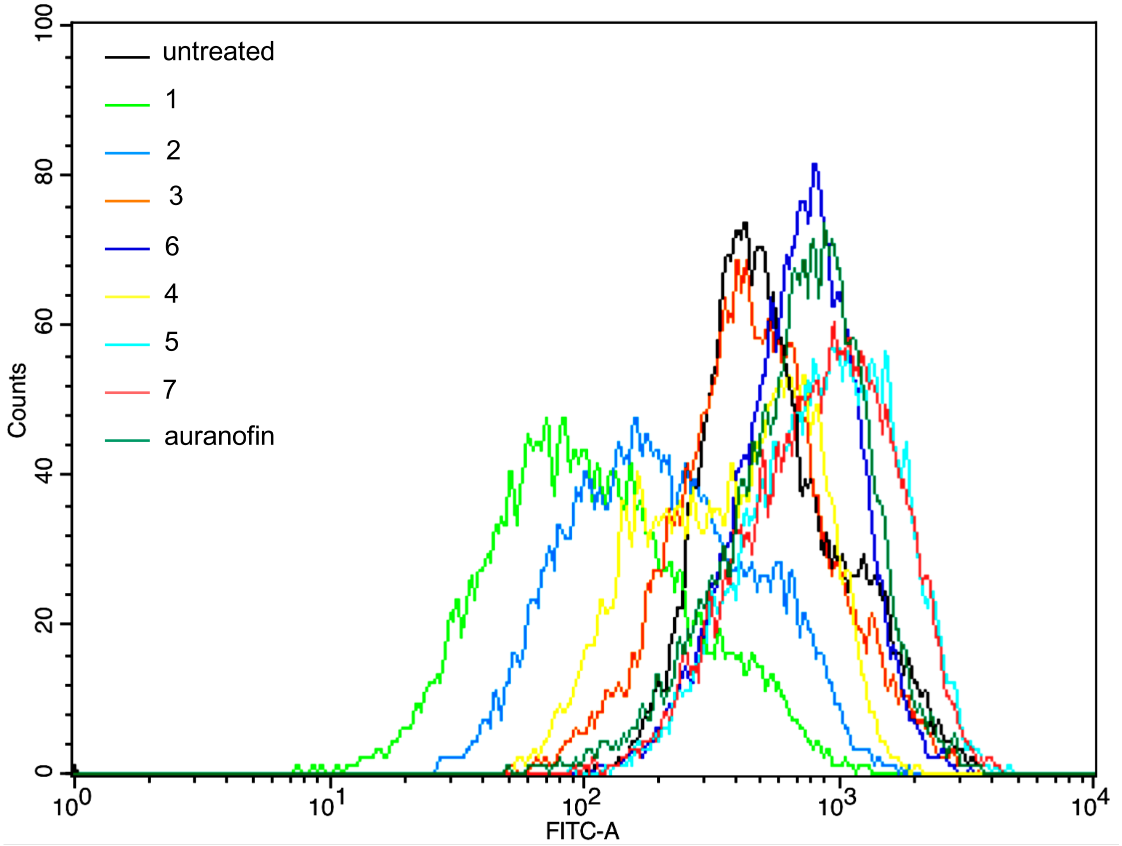

Figure S66. Mitochondrial membrane potential studies. Plots show untreated cells (negative control), and cells treated with **1 – 7** (5 μM, overnight) or auranofin (5 μM, overnight). Cells were then stained with rhodamine 123 and subjected to flow cytometry analysis.

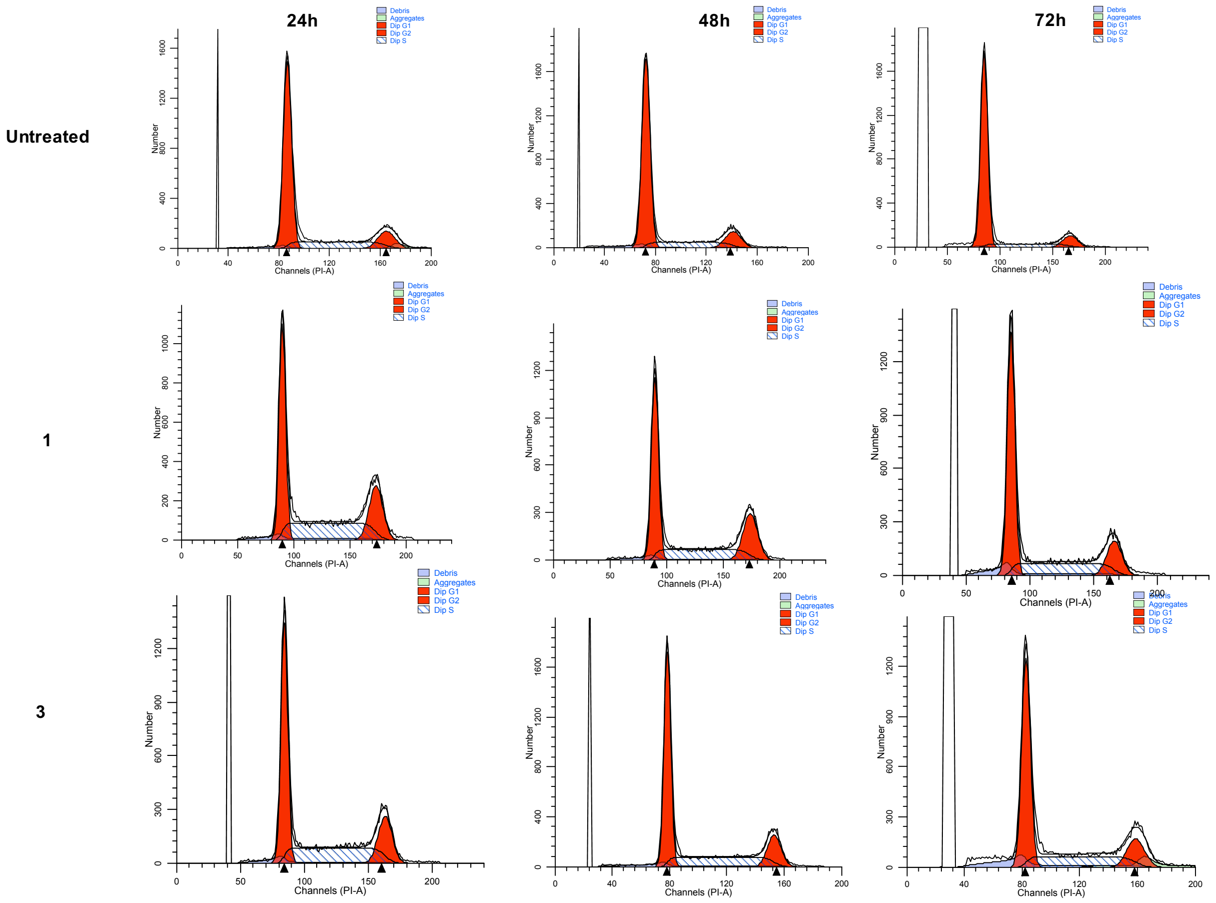

Figure S67. Histogram representing the different phases of the cell cycle of OVCAR8 in the presence or absence **1** (2 µM) or **3** (2 µM) over the course of 72h. 24h untreated: G1: 66.08%, S: 21.69%, G2/M: 12.23%, 48h untreated: G1: 70.21%, S: 17.74%, G2/M: 12.05%, 72h untreated: G1: 77.58%, S: 12.81%, G2/M: 9.62%. 24h treated with **1**: G1: 42.22%, S: 36.39%, G2/M: 21.38%, 48h treated with **1**: G1: 47.27%, S: 34.46%, G2/M: 18.26%, 72h treated with **1**: G1: 54.99%, S: 29.48%, G2/M: 15.53%. 24h treated with **3**: G1: 47.45%, S: 28.38%, G2/M: 24.17%, 48h treated with **3**: G1: 54.39%, S: 29.82%, G2/M: 15.79%, 72h treated with **3**: G1: 56.18%, S: 28.51%, G2/M: 15.31%.

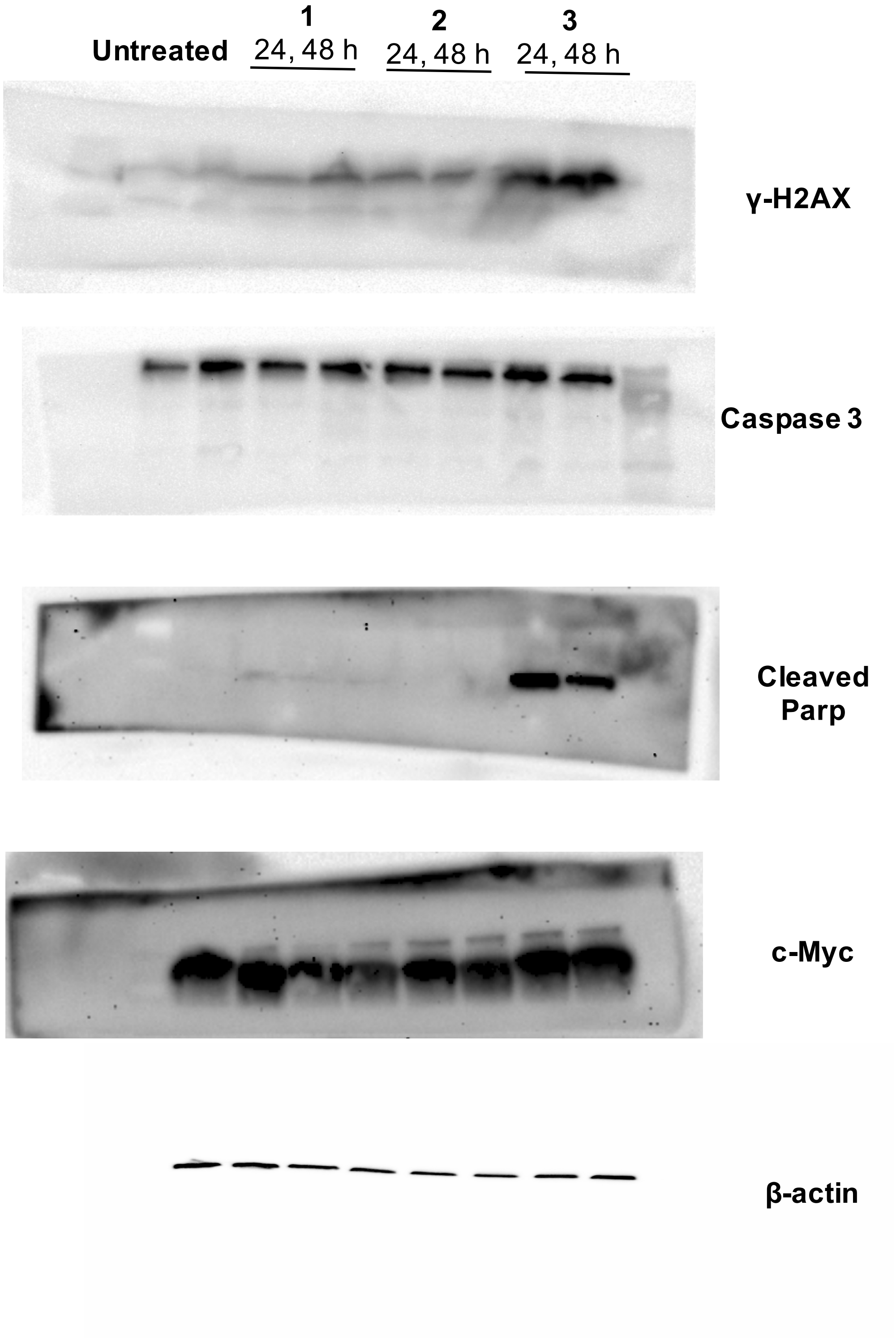

Figure S68. Full-length Western blots/gels for the expression of γ-H2AX, Caspase-3, Cleaved Parp, c-Myc and β-actin.

Supporting Tables

Table S1. Crystal data and structure refinement for complex **1**.

Empirical formula	C18 H28 Au2 Cl2 N2 P2
Formula weight	799.20
Temperature	90.0(2) K
Wavelength	0.71073 Å
Crystal system, space group	Orthorhombic, $P2_12_12_1$
Unit cell dimensions	a = 10.4707(3) Å alpha = 90°.<br>b = 12.1842(3) Å beta = 90°.<br>c = 18.1249(6) Å gamma = 90°.
Volume	2312.32(12) $Å^3$
Z, Calculated density	4, 2.296 Mg/m^3
Absorption coefficient	13.050 mm^{-1}
F(000)	1488
Crystal size	0.120 x 0.080 x 0.020 mm
Theta range for data collection	2.800 to 27.502 deg.
Limiting indices	-13<=h<=13, -14<=k<=15, -23<=l<=17
Reflections collected / unique	14588 / 5222 [R(int) = 0.0350]
Completeness to theta = 25.242	99.7 %
Absorption correction	Semi-empirical from equivalents
Max. and min. transmission	0.647 and 0.334
Refinement method	Full-matrix least-squares on F^2
Data / restraints / parameters	5222 / 0 / 243
Goodness-of-fit on F^2	1.065
Final R indices [I>2sigma(I)]	R1 = 0.0287, wR2 = 0.0530
R indices (all data)	R1 = 0.0371, wR2 = 0.0549
Extinction coefficient	n/a
Largest diff. peak and hole	1.957 and -1.443 e. $Å^{-3}$

Table S2. Crystal data and structure refinement for complex **2**.

Empirical formula	C36 H56 Au Cl N4 P4
Formula weight	901.14
Temperature	90.0(2) K
Wavelength	0.71073 Å
Crystal system, space group	Monoclinic, C2
Unit cell dimensions	a = 24.4547(8) Å alpha = 90 deg.<br>b = 13.2473(5) Å beta = 117.342(1) deg.<br>c = 14.9247(5) Å gamma = 90 deg.
Volume	4294.8(3) $Å^3$
Z, Calculated density	4, 1.394 Mg/m^3
Absorption coefficient	3.664 mm^{-1}
F(000)	1824
Crystal size	0.350 x 0.240 x 0.180 mm
Theta range for data collection	2.632 to 27.539 deg.
Limiting indices	-31<=h<=31, -17<=k<=17, -19<=l<=18
Reflections collected / unique	22198 / 9638 [R(int) = 0.0223]
Completeness to theta = 25.242	99.7 %
Absorption correction	Semi-empirical from equivalents
Max. and min. transmission	0.419 and 0.286
Refinement method	Full-matrix least-squares on F^2
Data / restraints / parameters	9638 / 1 / 442
Goodness-of-fit on F^2	1.089
Final R indices [I>2sigma(I)]	R1 = 0.0257, wR2 = 0.0703
R indices (all data)	R1 = 0.0276, wR2 = 0.0708
Extinction coefficient	0.00034(10)
Largest diff. peak and hole	1.719 and -0.511 e. $Å^{-3}$

Table S3. Crystal data and structure refinement for complex **4**.

Empirical formula	C112 H112 Au4 Cl4 N2 O17 P8
Formula weight	2935.46
Temperature	90.0(2) K
Wavelength	0.71073 Å
Crystal system, space group	Triclinic, P1
Unit cell dimensions	a = 12.1192(3) Å alpha = 99.5954(7) deg.<br>b = 12.3163(3) Å beta = 92.4085(7) deg.<br>c = 20.7389(4) Å gamma = 113.4072(7) deg.
Volume	2780.95(11) $Å^3$
Z, Calculated density	1, 1.753 Mg/m^3
Absorption coefficient	5.536 mm^{-1}
F(000)	1438
Crystal size	0.150 x 0.140 x 0.110 mm
Theta range for data collection	2.727 to 27.560 deg.
Limiting indices	-15<=h<=15, -15<=k<=16, -26<=l<=26
Reflections collected / unique	130251 / 25475 [R(int) = 0.0696]
Completeness to theta = 25.242	99.9 %
Absorption correction	Semi-empirical from equivalents
Max. and min. transmission	0.620 and 0.469
Refinement method	Full-matrix least-squares on F^2
Data / restraints / parameters	25475 / 65 / 1342
Goodness-of-fit on F^2	1.023
Final R indices [I>2sigma(I)]	R1 = 0.0199, wR2 = 0.0372
R indices (all data)	R1 = 0.0238, wR2 = 0.0378
Extinction coefficient	n/a
Largest diff. peak and hole	0.971 and -0.598 e. $Å^{-3}$

Table S4. Crystal data and structure refinement for complex **5**.

Empirical formula	C54 H51 Au Cl N O4 P4
Formula weight	1134.25
Temperature	90.0(2) K
Wavelength	0.71073 Å
Crystal system, space group	Orthorhombic, Pca2(1)
Unit cell dimensions	a = 21.6194(9) Å alpha = 90 deg.<br>b = 10.2687(4) Å beta = 90 deg.<br>c = 21.8943(7) Å gamma = 90 deg.
Volume	4860.6(3) $Å^3$
Z, Calculated density	4, 1.550 Mg/m^3
Absorption coefficient	3.261 mm^{-1}
F(000)	2280
Crystal size	0.140 x 0.140 x 0.060 mm
Theta range for data collection	2.720 to 27.516 deg.
Limiting indices	-28<=h<=28, -13<=k<=13, -28<=l<=28
Reflections collected / unique	137872 / 11126 [R(int) = 0.0793]
Completeness to theta = 25.242	99.8 %
Absorption correction	Semi-empirical from equivalents
Max. and min. transmission	0.813 and 0.639
Refinement method	Full-matrix least-squares on F^2
Data / restraints / parameters	11126 / 813 / 846
Goodness-of-fit on F^2	1.019
Final R indices [I>2sigma(I)]	R1 = 0.0200, wR2 = 0.0427
R indices (all data)	R1 = 0.0247, wR2 = 0.0439
Extinction coefficient	0.00042(8)
Largest diff. peak and hole	0.982 and -0.923 $e.A^{-3}$

Table S5. Bond lengths [Å] and angles [deg] for complex **1**.

Bond	Lengths		Bond	Lengths		Bond	Lengths
Au1-P1	2.225(2)		C13-H13B	0.98		C2-C1-P1	129.8(6)
Au1-Cl1	2.284(2)		C13-H13C	0.98		N2-C2-C1	120.8(8)
Au1-Au2	2.9963(5)		C14-H14A	0.98		N2-C2-P2	108.9(6)
Au2-P2	2.236(2)		C14-H14B	0.98		C1-C2-P2	130.3(6)
Au2-Cl2	2.290(2)		C14-H14C	0.98		N2-C3-C4	119.7(8)
P1-C9	1.82(1)		C15-C16	1.526(14)		N2-C3-C8	120.2(8)
P1-C10	1.843(10)		C15-C17	1.526(12)		C4-C3-C8	120.0(8)
P1-C1	1.855(9)		C15-C18	1.565(13)		C5-C4-C3	119.3(9)
P2-C14	1.828(9)		C16-H16A	0.98		C5-C4-H4	120.3
P2-C2	1.852(9)		C16-H16B	0.98		C3-C4-H4	120.3
P2-C15	1.868(10)		C16-H16C	0.98		C4-C5-C6	120.5(9)
N1-C1	1.337(11)		C17-H17A	0.98		C4-C5-H5	119.7
N1-C8	1.347(10)		C17-H17B	0.98		C6-C5-H5	119.7
N2-C2	1.328(10)		C17-H17C	0.98		C7-C6-C5	120.2(9)
N2-C3	1.362(11)		C18-H18A	0.98		C7-C6-H6	119.9
C1-C2	1.417(12)		C18-H18B	0.98		C5-C6-H6	119.9
C3-C4	1.401(11)		C18-H18C	0.98		C6-C7-C8	120.9(9)
C3-C8	1.404(12)		P1-Au1-Cl1	171.77(9)		C6-C7-H7	119.5
C4-C5	1.353(12)		P1-Au1-Au2	93.90(7)		C8-C7-H7	119.5
C4-H4	0.95		Cl1-Au1-Au2	94.32(7)		N1-C8-C7	120.9(9)
C5-C6	1.422(13)		P2-Au2-Cl2	170.96(9)		N1-C8-C3	120.0(8)
C5-H5	0.95		P2-Au2-Au1	90.91(6)		C7-C8-C3	119.0(8)
C6-C7	1.335(13)		Cl2-Au2-Au1	97.83(6)		P1-C9-H9A	109.5
C6-H6	0.95		C9-P1-C10	106.6(5)		P1-C9-H9AB	109.5
C7-C8	1.393(12)		C9-P1-C1	103.2(4)		H9A-C9-H9AB	109.5
C7-H7	0.95		C10-P1-C1	106.7(4)		P1-C9-H9AC	109.5
C9-H9A	0.98		C9-P1-Au1	108.1(3)		H9A-C9-H9AC	109.5
C9-H9AB	0.98		C10-P1-Au1	112.6(3)		H9AB-C9-H9AC	109.5
C9-H9AC	0.98		C1-P1-Au1	118.7(3)		C13-C10-C12	110.8(9)
C10-C13	1.502(14)		C14-P2-C2	102.5(4)		C13-C10-C11	108.1(9)
C10-C12	1.522(13)		C14-P2-C15	105.9(5)		C12-C10-C11	108.1(8)
C10-C11	1.537(14)		C2-P2-C15	105.6(4)		C13-C10-P1	107.8(6)
C11-H11A	0.98		C14-P2-Au2	108.4(3)		C12-C10-P1	108.1(7)
C11-H11B	0.98		C2-P2-Au2	120.3(3)		C11-C10-P1	114.0(7)
C11-H11C	0.98		C15-P2-Au2	112.8(3)		C10-C11-H11A	109.5
C12-H12A	0.98		C1-N1-C8	119.7(8)		C10-C11-H11B	109.5
C12-H12B	0.98		C2-N2-C3	119.0(8)		H11A-C11-H11B	109.5
C12-H12C	0.98		N1-C1-C2	120.1(8)		C10-C11-H11C	109.5
C13-H13A	0.98		N1-C1-P1	110.1(6)		H11A-C11-H11C	109.5

H11B-C11-H11C	109.5
C10-C12-H12A	109.5
C10-C12-H12B	109.5
H12A-C12-H12B	109.5
C10-C12-H12C	109.5
H12A-C12-H12C	109.5
H12B-C12-H12C	109.5
C10-C13-H13A	109.5
C10-C13-H13B	109.5
H13A-C13-H13B	109.5
C10-C13-H13C	109.5
H13A-C13-H13C	109.5
H13B-C13-H13C	109.5
P2-C14-H14A	109.5
P2-C14-H14B	109.5
H14A-C14-H14B	109.5
P2-C14-H14C	109.5
H14A-C14-H14C	109.5
H14B-C14-H14C	109.5
C16-C15-C17	109.0(8)
C16-C15-C18	110.4(8)
C17-C15-C18	111.4(8)
C16-C15-P2	108.6(7)
C17-C15-P2	112.5(7)
C18-C15-P2	104.9(7)
C15-C16-H16A	109.5
C15-C16-H16B	109.5
H16A-C16-H16B	109.5
C15-C16-H16C	109.5
H16A-C16-H16C	109.5
H16B-C16-H16C	109.5
C15-C17-H17A	109.5
C15-C17-H17B	109.5
H17A-C17-H17B	109.5
C15-C17-H17C	109.5
H17A-C17-H17C	109.5
H17B-C17-H17C	109.5
C15-C18-H18A	109.5
C15-C18-H18B	109.5
H18A-C18-H18B	109.5
C15-C18-H18C	109.5
H18A-C18-H18C	109.5
H18B-C18-H18C	109.5

Table S6. Bond lengths [Å] and angles [deg] for complex **2**.

Bond	Lengths		Bond	Lengths		Bond	Lengths
Au1-P1	2.3889(14)		C10-H10B	0.98		C27-C28	1.541(11)
Au1-P4	2.390(2)		C10-H10C	0.98		C28-H28A	0.98
Au1-P2	2.3928(19)		C11-H11A	0.98		C28-H28B	0.98
Au1-P3	2.3964(15)		C11-H11B	0.98		C28-H28C	0.98
P1-C13	1.823(7)		C11-H11C	0.98		C29-H29A	0.98
P1-C1	1.840(6)		C12-H12A	0.98		C29-H29B	0.98
P1-C9	1.865(7)		C12-H12B	0.98		C29-H29C	0.98
P2-C18	1.812(8)		C12-H12C	0.98		C30-H30A	0.98
P2-C2	1.814(7)		C13-H13A	0.98		C30-H30B	0.98
P2-C14	1.876(8)		C13-H13B	0.98		C30-H30C	0.98
P3-C31	1.830(6)		C13-H13C	0.98		C31-H31A	0.98
P3-C19	1.845(7)		C14-C16	1.521(10)		C31-H31B	0.98
P3-C27	1.854(7)		C14-C15	1.525(12)		C31-H31C	0.98
P4-C36	1.792(7)		C14-C17	1.537(9)		C32-C35	1.519(9)
P4-C20	1.845(9)		C15-H15A	0.98		C32-C33	1.523(9)
P4-C32	1.870(8)		C15-H15B	0.98		C32-C34	1.559(11)
N1-C1	1.323(7)		C15-H15C	0.98		C33-H33A	0.98
N1-C8	1.371(7)		C16-H16A	0.98		C33-H33B	0.98
N2-C2	1.338(9)		C16-H16B	0.98		C33-H33C	0.98
N2-C3	1.359(8)		C16-H16C	0.98		C34-H34A	0.98
N3-C19	1.323(8)		C17-H17A	0.98		C34-H34B	0.98
N3-C26	1.371(8)		C17-H17B	0.98		C34-H34C	0.98
N4-C20	1.325(10)		C17-H17C	0.98		C35-H35A	0.98
N4-C21	1.365(9)		C18-H18A	0.98		C35-H35B	0.98
C1-C2	1.438(9)		C18-H18B	0.98		C35-H35C	0.98
C3-C4	1.405(10)		C18-H18C	0.98		C36-H36A	0.98
C3-C8	1.430(8)		C19-C20	1.435(10)		C36-H36B	0.98
C4-C5	1.368(10)		C21-C26	1.39(1)		C36-H36C	0.98
C4-H4	0.95		C21-C22	1.413(12)		P1-Au1-P4	107.96(6)
C5-C6	1.395(9)		C22-C23	1.355(10)		P1-Au1-P2	87.91(5)
C5-H5	0.95		C22-H22	0.95		P4-Au1-P2	138.21(6)
C6-C7	1.376(9)		C23-C24	1.405(11)		P1-Au1-P3	137.50(6)
C6-H6	0.95		C23-H23	0.95		P4-Au1-P3	87.79(6)
C7-C8	1.395(8)		C24-C25	1.355(9)		P2-Au1-P3	106.48(6)
C7-H7	0.95		C24-H24	0.95		C13-P1-C1	101.6(3)
C9-C10	1.514(9)		C25-C26	1.431(9)		C13-P1-C9	104.5(3)
C9-C12	1.523(11)		C25-H25	0.95		C1-P1-C9	105.1(3)
C9-C11	1.527(9)		C27-C30	1.506(11)		C13-P1-Au1	114.6(2)
C10-H10A	0.98		C27-C29	1.523(8)		C1-P1-Au1	105.01(18)

C9-P1-Au1	123.6(2)		C5-C6-H6	119.6		C16-C14-P2	114.6(6)
C18-P2-C2	101.9(4)		C6-C7-C8	119.8(6)		C15-C14-P2	107.9(5)
C18-P2-C14	104.7(4)		C6-C7-H7	120.1		C17-C14-P2	104.0(5)
C2-P2-C14	103.4(3)		C8-C7-H7	120.1		C14-C15-H15A	109.5
C18-P2-Au1	115.0(3)		N1-C8-C7	120.5(5)		C14-C15-H15B	109.5
C2-P2-Au1	104.9(2)		N1-C8-C3	119.9(5)		H15A-C15-H15B	109.5
C14-P2-Au1	124.1(2)		C7-C8-C3	119.6(5)		C14-C15-H15C	109.5
C31-P3-C19	100.0(4)		C10-C9-C12	113.2(7)		H15A-C15-H15C	109.5
C31-P3-C27	105.2(3)		C10-C9-C11	108.6(6)		H15B-C15-H15C	109.5
C19-P3-C27	106.3(3)		C12-C9-C11	108.3(6)		C14-C16-H16A	109.5
C31-P3-Au1	113.7(2)		C10-C9-P1	113.8(5)		C14-C16-H16B	109.5
C19-P3-Au1	105.1(2)		C12-C9-P1	107.6(5)		H16A-C16-H16B	109.5
C27-P3-Au1	123.8(2)		C11-C9-P1	104.9(5)		C14-C16-H16C	109.5
C36-P4-C20	101.1(4)		C9-C10-H10A	109.5		H16A-C16-H16C	109.5
C36-P4-C32	105.5(4)		C9-C10-H10B	109.5		H16B-C16-H16C	109.5
C20-P4-C32	102.6(3)		H10A-C10-H10B	109.5		C14-C17-H17A	109.5
C36-P4-Au1	116.0(3)		C9-C10-H10C	109.5		C14-C17-H17B	109.5
C20-P4-Au1	104.3(3)		H10A-C10-H10C	109.5		H17A-C17-H17B	109.5
C32-P4-Au1	124.1(2)		H10B-C10-H10C	109.5		C14-C17-H17C	109.5
C1-N1-C8	118.1(5)		C9-C11-H11A	109.5		H17A-C17-H17C	109.5
C2-N2-C3	118.1(6)		C9-C11-H11B	109.5		H17B-C17-H17C	109.5
C19-N3-C26	116.3(6)		H11A-C11-H11B	109.5		P2-C18-H18A	109.5
C20-N4-C21	116.8(6)		C9-C11-H11C	109.5		P2-C18-H18B	109.5
N1-C1-C2	121.9(6)		H11A-C11-H11C	109.5		H18A-C18-H18B	109.5
N1-C1-P1	118.1(4)		H11B-C11-H11C	109.5		P2-C18-H18C	109.5
C2-C1-P1	119.9(4)		C9-C12-H12A	109.5		H18A-C18-H18C	109.5
N2-C2-C1	120.3(6)		C9-C12-H12B	109.5		H18B-C18-H18C	109.5
N2-C2-P2	118.0(5)		H12A-C12-H12B	109.5		N3-C19-C20	122.1(6)
C1-C2-P2	121.6(5)		C9-C12-H12C	109.5		N3-C19-P3	117.9(5)
N2-C3-C4	119.9(6)		H12A-C12-H12C	109.5		C20-C19-P3	120.0(5)
N2-C3-C8	121.2(5)		H12B-C12-H12C	109.5		N4-C20-C19	121.3(7)
C4-C3-C8	118.9(6)		P1-C13-H13A	109.5		N4-C20-P4	117.5(6)
C5-C4-C3	120.1(6)		P1-C13-H13B	109.5		C19-C20-P4	120.7(6)
C5-C4-H4	120		H13A-C13-H13B	109.5		N4-C21-C26	121.7(6)
C3-C4-H4	120		P1-C13-H13C	109.5		N4-C21-C22	118.3(6)
C4-C5-C6	120.8(6)		H13A-C13-H13C	109.5		C26-C21-C22	120.0(6)
C4-C5-H5	119.6		H13B-C13-H13C	109.5		C23-C22-C21	119.6(8)
C6-C5-H5	119.6		C16-C14-C15	108.7(7)		C23-C22-H22	120.2
C7-C6-C5	120.7(6)		C16-C14-C17	109.1(6)		C21-C22-H22	120.2
C7-C6-H6	119.6		C15-C14-C17	112.4(7)		C22-C23-C24	120.7(7)

C22-C23-H23	119.6
C24-C23-H23	119.6
C25-C24-C23	121.3(6)
C25-C24-H24	119.4
C23-C24-H24	119.4
C24-C25-C26	119.0(7)
C24-C25-H25	120.5
C26-C25-H25	120.5
N3-C26-C21	121.6(6)
N3-C26-C25	119.0(6)
C21-C26-C25	119.4(6)
C30-C27-C29	107.2(6)
C30-C27-C28	111.9(7)
C29-C27-C28	109.4(6)
C30-C27-P3	106.8(5)
C29-C27-P3	114.2(5)
C28-C27-P3	107.5(5)
C27-C28-H28A	109.5
C27-C28-H28B	109.5
H28A-C28-H28B	109.5
C27-C28-H28C	109.5
H28A-C28-H28C	109.5
H28B-C28-H28C	109.5
C27-C29-H29A	109.5
C27-C29-H29B	109.5
H29A-C29-H29B	109.5
C27-C29-H29C	109.5
H29A-C29-H29C	109.5
H29B-C29-H29C	109.5
C27-C30-H30A	109.5
C27-C30-H30B	109.5
H30A-C30-H30B	109.5
C27-C30-H30C	109.5
H30A-C30-H30C	109.5
H30B-C30-H30C	109.5
P3-C31-H31A	109.5
P3-C31-H31B	109.5
H31A-C31-H31B	109.5
P3-C31-H31C	109.5
H31A-C31-H31C	109.5
H31B-C31-H31C	109.5
C35-C32-C33	109.5(6)
C35-C32-C34	108.5(6)
C33-C32-C34	108.8(6)
C35-C32-P4	108.2(5)
C33-C32-P4	114.8(5)
C34-C32-P4	106.8(5)
C32-C33-H33A	109.5
C32-C33-H33B	109.5
H33A-C33-H33B	109.5
C32-C33-H33C	109.5
H33A-C33-H33C	109.5
H33B-C33-H33C	109.5
C32-C34-H34A	109.5
C32-C34-H34B	109.5
H34A-C34-H34B	109.5
C32-C34-H34C	109.5
H34A-C34-H34C	109.5
H34B-C34-H34C	109.5
C32-C35-H35A	109.5
C32-C35-H35B	109.5
H35A-C35-H35B	109.5
C32-C35-H35C	109.5
H35A-C35-H35C	109.5
H35B-C35-H35C	109.5
P4-C36-H36A	109.5
P4-C36-H36B	109.5
H36A-C36-H36B	109.5
P4-C36-H36C	109.5
H36A-C36-H36C	109.5
H36B-C36-H36C	109.5

Table S7. Bond lengths [Å] and angles [deg] for complex **4**.

Au1A-P1A	2.3128(12)		C13A-H13A	0.99		C34A-H34A	0.95
Au1A-P4A	2.3161(11)		C13A-H13B	0.99		C35A-C36A	1.365(9)
Au1A-Au2A	2.9682(3)		C14A-H14A	0.99		C35A-H35A	0.95
Au2A-P2A	2.3047(13)		C14A-H14B	0.99		C36A-C37A	1.377(10)
Au2A-P3A	2.3096(13)		C15A-C20A	1.386(7)		C36A-H36A	0.95
P1A-C1A	1.803(5)		C15A-C16A	1.392(7)		C37A-C38A	1.387(9)
P1A-C7A	1.811(5)		C16A-C17A	1.370(7)		C37A-H37A	0.95
P1A-C13A	1.832(5)		C16A-H16A	0.95		C38A-H38A	0.95
P2A-C21A	1.810(5)		C17A-C18A	1.383(7)		C39A-C40A	1.526(7)
P2A-C15A	1.811(5)		C17A-H17A	0.95		C39A-H39A	0.99
P2A-C14A	1.835(5)		C18A-C19A	1.378(8)		C39A-H39B	0.99
P3A-C33A	1.796(5)		C18A-H18A	0.95		C40A-H40A	0.99
P3A-C27A	1.810(5)		C19A-C20A	1.388(8)		C40A-H40B	0.99
P3A-C39A	1.839(4)		C19A-H19A	0.95		C41A-C42A	1.387(8)
P4A-C47A	1.803(5)		C20A-H20A	0.95		C41A-C46A	1.409(7)
P4A-C41A	1.816(5)		C21A-C22A	1.383(8)		C42A-C43A	1.389(8)
P4A-C40A	1.829(5)		C21A-C26A	1.386(7)		C42A-H42A	0.95
C1A-C6A	1.403(6)		C22A-C23A	1.380(8)		C43A-C44A	1.377(9)
C1A-C2A	1.403(7)		C22A-H22A	0.95		C43A-H43A	0.95
C2A-C3A	1.389(8)		C23A-C24A	1.359(9)		C44A-C45A	1.376(9)
C2A-H2A	0.95		C23A-H23A	0.95		C44A-H44A	0.95
C3A-C4A	1.369(8)		C24A-C25A	1.367(9)		C45A-C46A	1.383(8)
C3A-H3A	0.95		C24A-H24A	0.95		C45A-H45A	0.95
C4A-C5A	1.402(8)		C25A-C26A	1.404(8)		C46A-H46A	0.95
C4A-H4A	0.95		C25A-H25A	0.95		C47A-C48A	1.375(8)
C5A-C6A	1.389(8)		C26A-H26A	0.95		C47A-C52A	1.387(7)
C5A-H5A	0.95		C27A-C28A	1.390(8)		C48A-C49A	1.385(9)
C6A-H6A	0.95		C27A-C32A	1.398(7)		C48A-H48A	0.95
C7A-C8A	1.385(8)		C28A-C29A	1.389(8)		C49A-C50A	1.374(8)
C7A-C12A	1.408(8)		C28A-H28A	0.95		C49A-H49A	0.95
C8A-C9A	1.395(8)		C29A-C30A	1.378(9)		C50A-C51A	1.377(9)
C8A-H8A	0.95		C29A-H29A	0.95		C50A-H50A	0.95
C9A-C10A	1.365(9)		C30A-C31A	1.367(8)		C51A-C52A	1.375(8)
C9A-H9A	0.95		C30A-H30A	0.95		C51A-H51A	0.95
C10A-C11A	1.375(10)		C31A-C32A	1.398(7)		C52A-H52A	0.95
C10A-H10A	0.95		C31A-H31A	0.95		Au1B-P1B	2.3130(11)
C11A-C12A	1.383(9)		C32A-H32A	0.95		Au1B-P4B	2.3153(12)
C11A-H11A	0.95		C33A-C34A	1.398(7)		Au1B-Au2B	2.9866(2)
C12A-H12A	0.95		C33A-C38A	1.403(7)		Au2B-P3B	2.3033(12)
C13A-C14A	1.520(7)		C34A-C35A	1.379(8)		Au2B-P2B	2.3096(12)

P1B-C7B	1.815(5)		C15B-C16B	1.398(6)		C37B-C38B	1.385(7)
P1B-C1B	1.817(5)		C16B-C17B	1.398(7)		C37B-H37B	0.95
P1B-C13B	1.839(5)		C16B-H16B	0.95		C38B-H38B	0.95
P2B-C15B	1.803(5)		C17B-C18B	1.372(8)		C39B-C40B	1.530(7)
P2B-C14B	1.812(5)		C17B-H17B	0.95		C39B-H39C	0.99
P2B-C21B	1.816(5)		C18B-C19B	1.350(7)		C39B-H39D	0.99
P3B-C27B	1.807(5)		C18B-H18B	0.95		C40B-H40C	0.99
P3B-C33B	1.814(5)		C19B-C20B	1.393(7)		C40B-H40D	0.99
P3B-C39B	1.823(5)		C19B-H19B	0.95		C41B-C46B	1.382(8)
P4B-C47B	1.815(5)		C20B-H20B	0.95		C41B-C42B	1.398(8)
P4B-C41B	1.817(5)		C21B-C26B	1.376(8)		C42B-C43B	1.386(7)
P4B-C40B	1.833(5)		C21B-C22B	1.397(8)		C42B-H42B	0.95
C1B-C2B	1.390(6)		C22B-C23B	1.394(8)		C43B-C44B	1.373(9)
C1B-C6B	1.395(7)		C22B-H22B	0.95		C43B-H43B	0.95
C2B-C3B	1.385(7)		C23B-C24B	1.376(9)		C44B-C45B	1.351(9)
C2B-H2B	0.95		C23B-H23B	0.95		C44B-H44B	0.95
C3B-C4B	1.379(7)		C24B-C25B	1.368(8)		C45B-C46B	1.392(7)
C3B-H3B	0.95		C24B-H24B	0.95		C45B-H45B	0.95
C4B-C5B	1.379(7)		C25B-C26B	1.395(8)		C46B-H46B	0.95
C4B-H4B	0.95		C25B-H25B	0.95		C47B-C52B	1.382(6)
C5B-C6B	1.393(7)		C26B-H26B	0.95		C47B-C48B	1.388(7)
C5B-H5B	0.95		C27B-C28B	1.387(7)		C48B-C49B	1.388(8)
C6B-H6B	0.95		C27B-C32B	1.396(7)		C48B-H48B	0.95
C7B-C8B	1.392(7)		C28B-C29B	1.398(8)		C49B-C50B	1.372(7)
C7B-C12B	1.392(6)		C28B-H28B	0.95		C49B-H49B	0.95
C8B-C9B	1.388(8)		C29B-C30B	1.372(8)		C50B-C51B	1.388(8)
C8B-H8B	0.95		C29B-H29B	0.95		C50B-H50B	0.95
C9B-C10B	1.376(7)		C30B-C31B	1.378(8)		C51B-C52B	1.388(8)
C9B-H9B	0.95		C30B-H30B	0.95		C51B-H51B	0.95
C10B-C11B	1.384(8)		C31B-C32B	1.380(8)		C52B-H52B	0.95
C10B-H10B	0.95		C31B-H31B	0.95		Cl1-O3	1.401(6)
C11B-C12B	1.383(8)		C32B-H32B	0.95		Cl1-O2	1.414(4)
C11B-H11B	0.95		C33B-C38B	1.376(7)		Cl1-O1	1.443(5)
C12B-H12B	0.95		C33B-C34B	1.387(7)		Cl1-O4	1.449(4)
C13B-C14B	1.539(7)		C34B-C35B	1.380(9)		Cl2-O8	1.418(4)
C13B-H13C	0.99		C34B-H34B	0.95		Cl2-O7	1.422(4)
C13B-H13D	0.99		C35B-C36B	1.383(9)		Cl2-O5	1.443(4)
C14B-H14C	0.99		C35B-H35B	0.95		Cl2-O6	1.444(5)
C14B-H14D	0.99		C36B-C37B	1.380(8)		Cl3-O9	1.419(4)
C15B-C20B	1.384(7)		C36B-H36B	0.95		Cl3-O12	1.427(4)

Cl3-O10	1.429(4)		P3A-Au2A-Au1A	87.43(3)		C5A-C6A-C1A	120.1(5)
Cl3-O11	1.431(5)		C1A-P1A-C7A	108.2(2)		C5A-C6A-H6A	120
Cl4-O16	1.437(8)		C1A-P1A-C13A	106.4(2)		C1A-C6A-H6A	120
Cl4-O13	1.444(9)		C7A-P1A-C13A	103.3(2)		C8A-C7A-C12A	118.4(5)
Cl4-O14	1.453(8)		C1A-P1A-Au1A	110.38(15)		C8A-C7A-P1A	120.9(4)
Cl4-O15	1.460(9)		C7A-P1A-Au1A	112.05(16)		C12A-C7A-P1A	120.7(5)
Cl4'-O16'	1.438(8)		C13A-P1A-Au1A	116.08(17)		C7A-C8A-C9A	120.2(6)
Cl4'-O13'	1.443(9)		C21A-P2A-C15A	107.1(2)		C7A-C8A-H8A	119.9
Cl4'-O14'	1.452(9)		C21A-P2A-C14A	103.6(2)		C9A-C8A-H8A	119.9
Cl4'-O15'	1.457(9)		C15A-P2A-C14A	106.9(2)		C10A-C9A-C8A	120.7(6)
N1D-C1D	1.136(9)		C21A-P2A-Au2A	112.38(18)		C10A-C9A-H9A	119.6
C1D-C2D	1.444(10)		C15A-P2A-Au2A	112.50(16)		C8A-C9A-H9A	119.6
C2D-H2DA	0.98		C14A-P2A-Au2A	113.76(17)		C9A-C10A-C11A	119.9(6)
C2D-H2DB	0.98		C33A-P3A-C27A	108.1(2)		C9A-C10A-H10A	120.1
C2D-H2DC	0.98		C33A-P3A-C39A	107.4(2)		C11A-C10A-H10A	120.1
N2D-C3D	1.149(10)		C27A-P3A-C39A	104.2(2)		C10A-C11A-C12A	120.5(7)
C3D-C4D	1.421(12)		C33A-P3A-Au2A	111.57(16)		C10A-C11A-H11A	119.7
C4D-H4D1	0.98		C27A-P3A-Au2A	113.38(17)		C12A-C11A-H11A	119.7
C4D-H4D2	0.98		C39A-P3A-Au2A	111.85(17)		C11A-C12A-C7A	120.2(6)
C4D-H4D3	0.98		C47A-P4A-C41A	105.2(2)		C11A-C12A-H12A	119.9
O1E-C3E	1.392(10)		C47A-P4A-C40A	108.8(2)		C7A-C12A-H12A	119.9
O1E-C1E	1.43(1)		C41A-P4A-C40A	105.0(2)		C14A-C13A-P1A	118.2(3)
C1E-C2E	1.505(13)		C47A-P4A-Au1A	114.78(16)		C14A-C13A-H13A	107.8
C1E-H1EA	0.99		C41A-P4A-Au1A	105.95(17)		P1A-C13A-H13A	107.8
C1E-H1EB	0.99		C40A-P4A-Au1A	116.12(15)		C14A-C13A-H13B	107.8
C2E-H2EA	0.98		C6A-C1A-C2A	119.2(5)		P1A-C13A-H13B	107.8
C2E-H2EB	0.98		C6A-C1A-P1A	120.6(4)		H13A-C13A-H13B	107.1
C2E-H2EC	0.98		C2A-C1A-P1A	120.2(4)		C13A-C14A-P2A	117.2(3)
C3E-C4E	1.483(12)		C3A-C2A-C1A	119.5(5)		C13A-C14A-H14A	108
C3E-H3EA	0.99		C3A-C2A-H2A	120.3		P2A-C14A-H14A	108
C3E-H3EB	0.99		C1A-C2A-H2A	120.3		C13A-C14A-H14B	108
C4E-H4EA	0.98		C4A-C3A-C2A	121.7(5)		P2A-C14A-H14B	108
C4E-H4EB	0.98		C4A-C3A-H3A	119.2		H14A-C14A-H14B	107.2
C4E-H4EC	0.98		C2A-C3A-H3A	119.2		C20A-C15A-C16A	119.2(5)
			C3A-C4A-C5A	119.3(5)		C20A-C15A-P2A	118.6(4)
P1A-Au1A-P4A	168.66(5)		C3A-C4A-H4A	120.4		C16A-C15A-P2A	122.1(4)
P1A-Au1A-Au2A	94.40(3)		C5A-C4A-H4A	120.4		C17A-C16A-C15A	120.6(4)
P4A-Au1A-Au2A	94.62(3)		C6A-C5A-C4A	120.3(5)		C17A-C16A-H16A	119.7
P2A-Au2A-P3A	178.22(4)		C6A-C5A-H5A	119.9		C15A-C16A-H16A	119.7
P2A-Au2A-Au1A	91.42(3)		C4A-C5A-H5A	119.9		C16A-C17A-C18A	120.1(5)

C16A-C17A-H17A	119.9		C29A-C30A-H30A	119.9		C41A-C42A-C43A	120.0(6)
C18A-C17A-H17A	119.9		C30A-C31A-C32A	121.0(5)		C41A-C42A-H42A	120
C19A-C18A-C17A	119.9(5)		C30A-C31A-H31A	119.5		C43A-C42A-H42A	120
C19A-C18A-H18A	120.1		C32A-C31A-H31A	119.5		C44A-C43A-C42A	120.4(6)
C17A-C18A-H18A	120.1		C27A-C32A-C31A	119.0(5)		C44A-C43A-H43A	119.8
C18A-C19A-C20A	120.2(5)		C27A-C32A-H32A	120.5		C42A-C43A-H43A	119.8
C18A-C19A-H19A	119.9		C31A-C32A-H32A	120.5		C45A-C44A-C43A	119.7(5)
C20A-C19A-H19A	119.9		C34A-C33A-C38A	118.5(5)		C45A-C44A-H44A	120.1
C15A-C20A-C19A	119.9(5)		C34A-C33A-P3A	122.9(4)		C43A-C44A-H44A	120.1
C15A-C20A-H20A	120.1		C38A-C33A-P3A	118.6(4)		C44A-C45A-C46A	121.2(6)
C19A-C20A-H20A	120.1		C35A-C34A-C33A	120.6(5)		C44A-C45A-H45A	119.4
C22A-C21A-C26A	118.5(5)		C35A-C34A-H34A	119.7		C46A-C45A-H45A	119.4
C22A-C21A-P2A	123.6(4)		C33A-C34A-H34A	119.7		C45A-C46A-C41A	119.0(6)
C26A-C21A-P2A	117.9(4)		C36A-C35A-C34A	120.2(6)		C45A-C46A-H46A	120.5
C23A-C22A-C21A	121.2(6)		C36A-C35A-H35A	119.9		C41A-C46A-H46A	120.5
C23A-C22A-H22A	119.4		C34A-C35A-H35A	119.9		C48A-C47A-C52A	118.6(5)
C21A-C22A-H22A	119.4		C35A-C36A-C37A	120.7(6)		C48A-C47A-P4A	119.9(4)
C24A-C23A-C22A	120.1(6)		C35A-C36A-H36A	119.7		C52A-C47A-P4A	121.5(4)
C24A-C23A-H23A	120		C37A-C36A-H36A	119.7		C47A-C48A-C49A	120.8(5)
C22A-C23A-H23A	120		C36A-C37A-C38A	120.1(6)		C47A-C48A-H48A	119.6
C23A-C24A-C25A	120.4(6)		C36A-C37A-H37A	120		C49A-C48A-H48A	119.6
C23A-C24A-H24A	119.8		C38A-C37A-H37A	120		C50A-C49A-C48A	120.3(6)
C25A-C24A-H24A	119.8		C37A-C38A-C33A	119.9(6)		C50A-C49A-H49A	119.8
C24A-C25A-C26A	120.1(6)		C37A-C38A-H38A	120		C48A-C49A-H49A	119.8
C24A-C25A-H25A	119.9		C33A-C38A-H38A	120		C49A-C50A-C51A	119.0(6)
C26A-C25A-H25A	119.9		C40A-C39A-P3A	117.1(3)		C49A-C50A-H50A	120.5
C21A-C26A-C25A	119.7(6)		C40A-C39A-H39A	108		C51A-C50A-H50A	120.5
C21A-C26A-H26A	120.1		P3A-C39A-H39A	108		C52A-C51A-C50A	120.8(5)
C25A-C26A-H26A	120.1		C40A-C39A-H39B	108		C52A-C51A-H51A	119.6
C28A-C27A-C32A	119.5(5)		P3A-C39A-H39B	108		C50A-C51A-H51A	119.6
C28A-C27A-P3A	119.4(4)		H39A-C39A-H39B	107.3		C51A-C52A-C47A	120.4(5)
C32A-C27A-P3A	121.1(4)		C39A-C40A-P4A	119.9(4)		C51A-C52A-H52A	119.8
C29A-C28A-C27A	120.4(6)		C39A-C40A-H40A	107.4		C47A-C52A-H52A	119.8
C29A-C28A-H28A	119.8		P4A-C40A-H40A	107.4		P1B-Au1B-P4B	177.82(5)
C27A-C28A-H28A	119.8		C39A-C40A-H40B	107.4		P1B-Au1B-Au2B	87.60(3)
C30A-C29A-C28A	120.0(6)		P4A-C40A-H40B	107.4		P4B-Au1B-Au2B	90.23(3)
C30A-C29A-H29A	120		H40A-C40A-H40B	106.9		P3B-Au2B-P2B	166.80(5)
C28A-C29A-H29A	120		C42A-C41A-C46A	119.5(5)		P3B-Au2B-Au1B	97.06(3)
C31A-C30A-C29A	120.1(6)		C42A-C41A-P4A	117.0(4)		P2B-Au2B-Au1B	95.50(3)
C31A-C30A-H30A	119.9		C46A-C41A-P4A	123.5(4)		C7B-P1B-C1B	104.6(2)

C7B-P1B-C13B	108.9(2)		C1B-C6B-H6B	120.4		C19B-C18B-C17B	120.5(5)
C1B-P1B-C13B	106.1(2)		C8B-C7B-C12B	119.5(5)		C19B-C18B-H18B	119.8
C7B-P1B-Au1B	109.42(15)		C8B-C7B-P1B	117.1(3)		C17B-C18B-H18B	119.8
C1B-P1B-Au1B	111.76(15)		C12B-C7B-P1B	123.1(4)		C18B-C19B-C20B	120.6(6)
C13B-P1B-Au1B	115.49(17)		C9B-C8B-C7B	120.3(4)		C18B-C19B-H19B	119.7
C15B-P2B-C14B	107.8(2)		C9B-C8B-H8B	119.8		C20B-C19B-H19B	119.7
C15B-P2B-C21B	108.5(2)		C7B-C8B-H8B	119.8		C15B-C20B-C19B	120.3(5)
C14B-P2B-C21B	105.3(2)		C10B-C9B-C8B	119.7(5)		C15B-C20B-H20B	119.9
C15B-P2B-Au2B	113.04(17)		C10B-C9B-H9B	120.2		C19B-C20B-H20B	119.9
C14B-P2B-Au2B	116.76(15)		C8B-C9B-H9B	120.2		C26B-C21B-C22B	119.7(5)
C21B-P2B-Au2B	104.84(16)		C9B-C10B-C11B	120.4(5)		C26B-C21B-P2B	123.0(4)
C27B-P3B-C33B	109.2(2)		C9B-C10B-H10B	119.8		C22B-C21B-P2B	116.8(4)
C27B-P3B-C39B	108.7(2)		C11B-C10B-H10B	119.8		C23B-C22B-C21B	120.2(6)
C33B-P3B-C39B	100.5(2)		C12B-C11B-C10B	120.3(5)		C23B-C22B-H22B	119.9
C27B-P3B-Au2B	111.95(17)		C12B-C11B-H11B	119.8		C21B-C22B-H22B	119.9
C33B-P3B-Au2B	110.42(15)		C10B-C11B-H11B	119.8		C24B-C23B-C22B	119.5(6)
C39B-P3B-Au2B	115.47(17)		C11B-C12B-C7B	119.7(5)		C24B-C23B-H23B	120.3
C47B-P4B-C41B	108.4(2)		C11B-C12B-H12B	120.2		C22B-C23B-H23B	120.2
C47B-P4B-C40B	107.7(2)		C7B-C12B-H12B	120.2		C25B-C24B-C23B	120.2(6)
C41B-P4B-C40B	101.2(2)		C14B-C13B-P1B	116.3(3)		C25B-C24B-H24B	119.9
C47B-P4B-Au1B	108.76(14)		C14B-C13B-H13C	108.2		C23B-C24B-H24B	119.9
C41B-P4B-Au1B	115.73(17)		P1B-C13B-H13C	108.2		C24B-C25B-C26B	121.2(6)
C40B-P4B-Au1B	114.62(16)		C14B-C13B-H13D	108.2		C24B-C25B-H25B	119.4
C2B-C1B-C6B	120.3(5)		P1B-C13B-H13D	108.2		C26B-C25B-H25B	119.4
C2B-C1B-P1B	117.0(4)		H13C-C13B-H13D	107.4		C21B-C26B-C25B	119.2(5)
C6B-C1B-P1B	122.7(4)		C13B-C14B-P2B	118.0(4)		C21B-C26B-H26B	120.4
C3B-C2B-C1B	119.4(5)		C13B-C14B-H14C	107.8		C25B-C26B-H26B	120.4
C3B-C2B-H2B	120.3		P2B-C14B-H14C	107.8		C28B-C27B-C32B	119.3(5)
C1B-C2B-H2B	120.3		C13B-C14B-H14D	107.8		C28B-C27B-P3B	118.9(4)
C4B-C3B-C2B	120.7(5)		P2B-C14B-H14D	107.8		C32B-C27B-P3B	121.7(4)
C4B-C3B-H3B	119.7		H14C-C14B-H14D	107.1		C27B-C28B-C29B	120.6(5)
C2B-C3B-H3B	119.7		C20B-C15B-C16B	118.8(5)		C27B-C28B-H28B	119.7
C3B-C4B-C5B	120.0(5)		C20B-C15B-P2B	120.1(4)		C29B-C28B-H28B	119.7
C3B-C4B-H4B	120		C16B-C15B-P2B	121.0(4)		C30B-C29B-C28B	118.9(6)
C5B-C4B-H4B	120		C15B-C16B-C17B	119.7(5)		C30B-C29B-H29B	120.6
C4B-C5B-C6B	120.3(5)		C15B-C16B-H16B	120.2		C28B-C29B-H29B	120.6
C4B-C5B-H5B	119.8		C17B-C16B-H16B	120.2		C29B-C30B-C31B	121.1(5)
C6B-C5B-H5B	119.8		C18B-C17B-C16B	120.1(5)		C29B-C30B-H30B	119.4
C5B-C6B-C1B	119.3(4)		C18B-C17B-H17B	119.9		C31B-C30B-H30B	119.4
C5B-C6B-H6B	120.4		C16B-C17B-H17B	119.9		C30B-C31B-C32B	120.3(5)

C30B-C31B-H31B	119.9		C41B-C42B-H42B	120.1		O8-Cl2-O6	108.7(3)
C32B-C31B-H31B	119.9		C44B-C43B-C42B	120.7(6)		O7-Cl2-O6	108.6(3)
C31B-C32B-C27B	119.8(5)		C44B-C43B-H43B	119.7		O5-Cl2-O6	108.8(3)
C31B-C32B-H32B	120.1		C42B-C43B-H43B	119.7		O9-Cl3-O12	109.5(3)
C27B-C32B-H32B	120.1		C45B-C44B-C43B	119.8(5)		O9-Cl3-O10	109.2(3)
C38B-C33B-C34B	118.5(5)		C45B-C44B-H44B	120.1		O12-Cl3-O10	109.5(3)
C38B-C33B-P3B	119.7(4)		C43B-C44B-H44B	120.1		O9-Cl3-O11	108.1(3)
C34B-C33B-P3B	121.6(4)		C44B-C45B-C46B	121.0(6)		O12-Cl3-O11	111.0(3)
C35B-C34B-C33B	120.2(6)		C44B-C45B-H45B	119.5		O10-Cl3-O11	109.5(3)
C35B-C34B-H34B	119.9		C46B-C45B-H45B	119.5		O16-Cl4-O13	109.1(11)
C33B-C34B-H34B	119.9		C41B-C46B-C45B	120.0(6)		O16-Cl4-O14	109.8(10)
C34B-C35B-C36B	120.9(6)		C41B-C46B-H46B	120		O13-Cl4-O14	108.2(10)
C34B-C35B-H35B	119.6		C45B-C46B-H46B	120		O16-Cl4-O15	112.6(7)
C36B-C35B-H35B	119.6		C52B-C47B-C48B	119.5(5)		O13-Cl4-O15	108.3(9)
C37B-C36B-C35B	119.2(6)		C52B-C47B-P4B	119.3(4)		O14-Cl4-O15	108.7(10)
C37B-C36B-H36B	120.4		C48B-C47B-P4B	121.1(4)		O16'-Cl4'-O13'	109.4(11)
C35B-C36B-H36B	120.4		C49B-C48B-C47B	119.7(5)		O16'-Cl4'-O14'	109.8(10)
C36B-C37B-C38B	119.5(5)		C49B-C48B-H48B	120.1		O13'-Cl4'-O14'	108.4(11)
C36B-C37B-H37B	120.2		C47B-C48B-H48B	120.1		O16'-Cl4'-O15'	111.6(8)
C38B-C37B-H37B	120.2		C50B-C49B-C48B	120.5(5)		O13'-Cl4'-O15'	108.6(11)
C33B-C38B-C37B	121.6(5)		C50B-C49B-H49B	119.7		O14'-Cl4'-O15'	109.0(11)
C33B-C38B-H38B	119.2		C48B-C49B-H49B	119.7		N1D-C1D-C2D	178.6(8)
C37B-C38B-H38B	119.2		C49B-C50B-C51B	120.2(5)		C1D-C2D-H2DA	109.5
C40B-C39B-P3B	120.0(4)		C49B-C50B-H50B	119.9		C1D-C2D-H2DB	109.5
C40B-C39B-H39C	107.3		C51B-C50B-H50B	119.9		H2DA-C2D-H2DB	109.5
P3B-C39B-H39C	107.3		C50B-C51B-C52B	119.3(5)		C1D-C2D-H2DC	109.5
C40B-C39B-H39D	107.3		C50B-C51B-H51B	120.4		H2DA-C2D-H2DC	109.5
P3B-C39B-H39D	107.3		C52B-C51B-H51B	120.4		H2DB-C2D-H2DC	109.5
H39C-C39B-H39D	106.9		C47B-C52B-C51B	120.7(5)		N2D-C3D-C4D	176.9(8)
C39B-C40B-P4B	119.1(3)		C47B-C52B-H52B	119.6		C3D-C4D-H4D1	109.5
C39B-C40B-H40C	107.5		C51B-C52B-H52B	119.6		C3D-C4D-H4D2	109.5
P4B-C40B-H40C	107.5		O3-Cl1-O2	114.9(4)		H4D1-C4D-H4D2	109.5
C39B-C40B-H40D	107.5		O3-Cl1-O1	108.6(3)		C3D-C4D-H4D3	109.5
P4B-C40B-H40D	107.5		O2-Cl1-O1	109.5(3)		H4D1-C4D-H4D3	109.5
H40C-C40B-H40D	107		O3-Cl1-O4	108.4(4)		H4D2-C4D-H4D3	109.5
C46B-C41B-C42B	118.8(5)		O2-Cl1-O4	106.8(3)		C3E-O1E-C1E	117.2(8)
C46B-C41B-P4B	123.1(4)		O1-Cl1-O4	108.4(3)		O1E-C1E-C2E	111.7(8)
C42B-C41B-P4B	118.0(4)		O8-Cl2-O7	111.6(3)		O1E-C1E-H1EA	109.3
C43B-C42B-C41B	119.8(6)		O8-Cl2-O5	109.2(2)		C2E-C1E-H1EA	109.3
C43B-C42B-H42B	120.1		O7-Cl2-O5	109.9(3)		O1E-C1E-H1EB	109.3

C2E-C1E-H1EB	109.3
H1EA-C1E-H1EB	107.9
C1E-C2E-H2EA	109.5
C1E-C2E-H2EB	109.5
H2EA-C2E-H2EB	109.5
C1E-C2E-H2EC	109.5
H2EA-C2E-H2EC	109.5
H2EB-C2E-H2EC	109.5
O1E-C3E-C4E	105.9(7)
O1E-C3E-H3EA	110.6
C4E-C3E-H3EA	110.6
O1E-C3E-H3EB	110.6
C4E-C3E-H3EB	110.6
H3EA-C3E-H3EB	108.7
C3E-C4E-H4EA	109.5
C3E-C4E-H4EB	109.5
H4EA-C4E-H4EB	109.5
C3E-C4E-H4EC	109.5
H4EA-C4E-H4EC	109.5
H4EB-C4E-H4EC	109.5

Table S8. Bond lengths [Å] and angles [deg] for complex **5**.

Au1-P4'	2.343(9)		C15-C20	1.401(5)		C34-C35	1.382(12)
Au1-P3	2.387(4)		C16-C17	1.385(6)		C34-H34	0.95
Au1-P2	2.3916(10)		C16-H16	0.95		C35-C36	1.374(12)
Au1-P1	2.4106(10)		C17-C18	1.381(6)		C35-H35	0.95
Au1-P4	2.433(6)		C17-H17	0.95		C36-C37	1.367(12)
Au1-P3'	2.472(6)		C18-C19	1.391(6)		C36-H36	0.95
P1-C1	1.806(4)		C18-H18	0.95		C37-C38	1.384(12)
P1-C7	1.823(4)		C19-C20	1.383(6)		C37-H37	0.95
P1-C13	1.834(4)		C19-H19	0.95		C38-H38	0.95
P2-C21	1.819(4)		C20-H20	0.95		C39-C40	1.533(9)
P2-C15	1.820(4)		C21-C26	1.387(6)		C39-H39A	0.99
P2-C14	1.843(4)		C21-C22	1.400(5)		C39-H39B	0.99
C1-C6	1.379(6)		C22-C23	1.382(5)		C40-H40A	0.99
C1-C2	1.400(6)		C22-H22	0.95		C40-H40B	0.99
C2-C3	1.389(6)		C23-C24	1.374(6)		C41-C42	1.392(12)
C2-H2	0.95		C23-H23	0.95		C41-C46	1.405(13)
C3-C4	1.375(6)		C24-C25	1.391(7)		C42-C43	1.390(13)
C3-H3	0.95		C24-H24	0.95		C42-H42	0.95
C4-C5	1.377(6)		C25-C26	1.390(6)		C43-C44	1.368(13)
C4-H4	0.95		C25-H25	0.95		C43-H43	0.95
C5-C6	1.407(6)		C26-H26	0.95		C44-C45	1.385(13)
C5-H5	0.95		P3-C33	1.827(9)		C44-H44	0.95
C6-H6	0.95		P3-C27	1.827(9)		C45-C46	1.364(13)
C7-C8	1.388(5)		P3-C39	1.847(8)		C45-H45	0.95
C7-C12	1.388(5)		P4-C41	1.807(11)		C46-H46	0.95
C8-C9	1.379(6)		P4-C47	1.827(10)		C47-C48	1.385(12)
C8-H8	0.95		P4-C40	1.836(9)		C47-C52	1.401(12)
C9-C10	1.375(6)		C27-C28	1.382(11)		C48-C49	1.392(12)
C9-H9	0.95		C27-C32	1.399(11)		C48-H48	0.95
C10-C11	1.372(6)		C28-C29	1.398(12)		C49-C50	1.405(14)
C10-H10	0.95		C28-H28	0.95		C49-H49	0.95
C11-C12	1.389(6)		C29-C30	1.369(13)		C50-C51	1.364(13)
C11-H11	0.95		C29-H29	0.95		C50-H50	0.95
C12-H12	0.95		C30-C31	1.400(13)		C51-C52	1.392(11)
C13-C14	1.542(6)		C30-H30	0.95		C51-H51	0.95
C13-H13A	0.99		C31-C32	1.390(11)		C52-H52	0.95
C13-H13B	0.99		C31-H31	0.95		P3'-C27'	1.817(12)
C14-H14A	0.99		C32-H32	0.95		P3'-C33'	1.820(12)
C14-H14B	0.99		C33-C38	1.381(11)		P3'-C39'	1.844(10)
C15-C16	1.393(5)		C33-C34	1.397(12)		P4'-C41'	1.819(14)

P4'-C47'	1.829(13)		C47'-C48'	1.382(15)		C7-P1-Au1	121.29(13)
P4'-C40'	1.851(10)		C47'-C52'	1.396(15)		C13-P1-Au1	103.35(13)
C27'-C32'	1.384(14)		C48'-C49'	1.382(15)		C21-P2-C15	104.71(17)
C27'-C28'	1.397(14)		C48'-H48'	0.95		C21-P2-C14	101.46(18)
C28'-C29'	1.374(14)		C49'-C50'	1.398(16)		C15-P2-C14	102.44(17)
C28'-H28'	0.95		C49'-H49'	0.95		C21-P2-Au1	119.57(13)
C29'-C30'	1.376(15)		C50'-C51'	1.377(16)		C15-P2-Au1	122.21(14)
C29'-H29'	0.95		C50'-H50'	0.95		C14-P2-Au1	102.97(14)
C30'-C31'	1.387(15)		C51'-C52'	1.384(15)		C6-C1-C2	119.9(4)
C30'-H30'	0.95		C51'-H51'	0.95		C6-C1-P1	123.6(4)
C31'-C32'	1.380(14)		C52'-H52'	0.95		C2-C1-P1	116.4(3)
C31'-H31'	0.95		Cl1-O3	1.431(13)		C3-C2-C1	119.7(4)
C32'-H32'	0.95		Cl1-O4	1.436(14)		C3-C2-H2	120.2
C33'-C38'	1.379(14)		Cl1-O1	1.445(13)		C1-C2-H2	120.2
C33'-C34'	1.409(14)		Cl1-O2	1.447(14)		C4-C3-C2	120.1(4)
C34'-C35'	1.384(15)		Cl1'-O3'	1.435(13)		C4-C3-H3	120
C34'-H34'	0.95		Cl1'-O2'	1.437(14)		C2-C3-H3	120
C35'-C36'	1.356(15)		Cl1'-O4'	1.439(14)		C3-C4-C5	120.9(4)
C35'-H35'	0.95		Cl1'-O1'	1.450(12)		C3-C4-H4	119.5
C36'-C37'	1.378(16)		N1S-C1S	1.137(6)		C5-C4-H4	119.5
C36'-H36'	0.95		C1S-C2S	1.460(7)		C4-C5-C6	119.5(4)
C37'-C38'	1.381(15)		C2S-H2SA	0.98		C4-C5-H5	120.3
C37'-H37'	0.95		C2S-H2SB	0.98		C6-C5-H5	120.3
C38'-H38'	0.95		C2S-H2SC	0.98		C1-C6-C5	119.9(5)
C39'-C40'	1.521(11)					C1-C6-H6	120
C39'-H39C	0.99		P4'-Au1-P2	126.9(2)		C5-C6-H6	120
C39'-H39D	0.99		P3-Au1-P2	123.87(10)		C8-C7-C12	118.4(4)
C40'-H40C	0.99		P4'-Au1-P1	125.51(15)		C8-C7-P1	121.2(3)
C40'-H40D	0.99		P3-Au1-P1	118.46(9)		C12-C7-P1	120.4(3)
C41'-C42'	1.397(15)		P2-Au1-P1	85.97(3)		C9-C8-C7	120.5(4)
C41'-C46'	1.399(16)		P3-Au1-P4	85.96(16)		C9-C8-H8	119.7
C42'-C43'	1.389(15)		P2-Au1-P4	129.60(15)		C7-C8-H8	119.7
C42'-H42'	0.95		P1-Au1-P4	116.67(12)		C10-C9-C8	120.9(4)
C43'-C44'	1.374(15)		P4'-Au1-P3'	86.1(2)		C10-C9-H9	119.6
C43'-H43'	0.95		P2-Au1-P3'	127.43(13)		C8-C9-H9	119.6
C44'-C45'	1.393(16)		P1-Au1-P3'	108.0(1)		C11-C10-C9	119.3(4)
C44'-H44'	0.95		C1-P1-C7	103.14(16)		C11-C10-H10	120.4
C45'-C46'	1.375(15)		C1-P1-C13	106.1(2)		C9-C10-H10	120.4
C45'-H45'	0.95		C7-P1-C13	102.84(18)		C10-C11-C12	120.4(4)
C46'-H46'	0.95		C1-P1-Au1	118.23(13)		C10-C11-H11	119.8

C12-C11-H11	119.8		C24-C23-C22	120.3(4)		C31-C32-H32	119.7
C7-C12-C11	120.5(4)		C24-C23-H23	119.8		C27-C32-H32	119.7
C7-C12-H12	119.7		C22-C23-H23	119.8		C38-C33-C34	118.8(9)
C11-C12-H12	119.7		C23-C24-C25	120.0(4)		C38-C33-P3	118.9(8)
C14-C13-P1	108.0(3)		C23-C24-H24	120		C34-C33-P3	122.2(8)
C14-C13-H13A	110.1		C25-C24-H24	120		C35-C34-C33	119.5(10)
P1-C13-H13A	110.1		C26-C25-C24	119.7(4)		C35-C34-H34	120.2
C14-C13-H13B	110.1		C26-C25-H25	120.1		C33-C34-H34	120.2
P1-C13-H13B	110.1		C24-C25-H25	120.1		C36-C35-C34	120.8(10)
H13A-C13-H13B	108.4		C21-C26-C25	120.7(4)		C36-C35-H35	119.6
C13-C14-P2	110.6(2)		C21-C26-H26	119.7		C34-C35-H35	119.6
C13-C14-H14A	109.5		C25-C26-H26	119.7		C37-C36-C35	120(1)
P2-C14-H14A	109.5		C33-P3-C27	103.7(5)		C37-C36-H36	120
C13-C14-H14B	109.5		C33-P3-C39	100.3(5)		C35-C36-H36	120
P2-C14-H14B	109.5		C27-P3-C39	107.7(4)		C36-C37-C38	119.9(10)
H14A-C14-H14B	108.1		C33-P3-Au1	122.3(4)		C36-C37-H37	120.1
C16-C15-C20	118.6(4)		C27-P3-Au1	116.1(4)		C38-C37-H37	120.1
C16-C15-P2	118.7(3)		C39-P3-Au1	104.9(3)		C33-C38-C37	120.8(9)
C20-C15-P2	122.6(3)		C41-P4-C47	102.5(6)		C33-C38-H38	119.6
C17-C16-C15	120.5(4)		C41-P4-C40	104.0(5)		C37-C38-H38	119.6
C17-C16-H16	119.8		C47-P4-C40	103.7(5)		C40-C39-P3	113.6(5)
C15-C16-H16	119.8		C41-P4-Au1	126.3(5)		C40-C39-H39A	108.8
C18-C17-C16	121.0(4)		C47-P4-Au1	117.5(4)		P3-C39-H39A	108.8
C18-C17-H17	119.5		C40-P4-Au1	99.8(3)		C40-C39-H39B	108.8
C16-C17-H17	119.5		C28-C27-C32	119.3(9)		P3-C39-H39B	108.8
C17-C18-C19	118.8(4)		C28-C27-P3	124.8(8)		H39A-C39-H39B	107.7
C17-C18-H18	120.6		C32-C27-P3	115.8(7)		C39-C40-P4	110.6(5)
C19-C18-H18	120.6		C27-C28-C29	120(1)		C39-C40-H40A	109.5
C20-C19-C18	120.9(4)		C27-C28-H28	120		P4-C40-H40A	109.5
C20-C19-H19	119.6		C29-C28-H28	120		C39-C40-H40B	109.5
C18-C19-H19	119.6		C30-C29-C28	120.7(10)		P4-C40-H40B	109.5
C19-C20-C15	120.2(4)		C30-C29-H29	119.7		H40A-C40-H40B	108.1
C19-C20-H20	119.9		C28-C29-H29	119.7		C42-C41-C46	119(1)
C15-C20-H20	119.9		C29-C30-C31	120(1)		C42-C41-P4	122.4(9)
C26-C21-C22	118.7(4)		C29-C30-H30	120		C46-C41-P4	118.6(9)
C26-C21-P2	120.1(3)		C31-C30-H30	120		C43-C42-C41	119.4(10)
C22-C21-P2	121.2(3)		C32-C31-C30	119.4(10)		C43-C42-H42	120.3
C23-C22-C21	120.5(4)		C32-C31-H31	120.3		C41-C42-H42	120.3
C23-C22-H22	119.7		C30-C31-H31	120.3		C44-C43-C42	121.0(9)
C21-C22-H22	119.7		C31-C32-C27	120.5(10)		C44-C43-H43	119.5

C42-C43-H43	119.5		C32'-C27'-C28'	118.1(11)		P3'-C39'-H39D	109.4
C43-C44-C45	119.7(10)		C32'-C27'-P3'	119.5(10)		H39C-C39'-H39D	108
C43-C44-H44	120.1		C28'-C27'-P3'	122.4(10)		C39'-C40'-P4'	116.8(7)
C45-C44-H44	120.1		C29'-C28'-C27'	119.6(12)		C39'-C40'-H40C	108.1
C46-C45-C44	120.4(11)		C29'-C28'-H28'	120.2		P4'-C40'-H40C	108.1
C46-C45-H45	119.8		C27'-C28'-H28'	120.2		C39'-C40'-H40D	108.1
C44-C45-H45	119.8		C28'-C29'-C30'	121.4(12)		P4'-C40'-H40D	108.1
C45-C46-C41	120.5(12)		C28'-C29'-H29'	119.3		H40C-C40'-H40D	107.3
C45-C46-H46	119.8		C30'-C29'-H29'	119.3		C42'-C41'-C46'	117.1(12)
C41-C46-H46	119.8		C29'-C30'-C31'	120.0(13)		C42'-C41'-P4'	123.5(11)
C48-C47-C52	118.6(9)		C29'-C30'-H30'	120		C46'-C41'-P4'	119.0(11)
C48-C47-P4	120.7(8)		C31'-C30'-H30'	120		C43'-C42'-C41'	119.6(12)
C52-C47-P4	120.7(8)		C32'-C31'-C30'	118.3(13)		C43'-C42'-H42'	120.2
C47-C48-C49	121.2(10)		C32'-C31'-H31'	120.9		C41'-C42'-H42'	120.2
C47-C48-H48	119.4		C30'-C31'-H31'	120.9		C44'-C43'-C42'	122.8(12)
C49-C48-H48	119.4		C31'-C32'-C27'	122.5(12)		C44'-C43'-H43'	118.6
C48-C49-C50	119(1)		C31'-C32'-H32'	118.7		C42'-C43'-H43'	118.6
C48-C49-H49	120.5		C27'-C32'-H32'	118.7		C43'-C44'-C45'	117.8(12)
C50-C49-H49	120.5		C38'-C33'-C34'	116.1(11)		C43'-C44'-H44'	121.1
C51-C50-C49	120.2(9)		C38'-C33'-P3'	120.5(10)		C45'-C44'-H44'	121.1
C51-C50-H50	119.9		C34'-C33'-P3'	123.4(10)		C46'-C45'-C44'	119.9(14)
C49-C50-H50	119.9		C35'-C34'-C33'	122.1(13)		C46'-C45'-H45'	120.1
C50-C51-C52	120.6(9)		C35'-C34'-H34'	118.9		C44'-C45'-H45'	120.1
C50-C51-H51	119.7		C33'-C34'-H34'	118.9		C45'-C46'-C41'	122.6(14)
C52-C51-H51	119.7		C36'-C35'-C34'	120.0(13)		C45'-C46'-H46'	118.7
C51-C52-C47	120.2(9)		C36'-C35'-H35'	120		C41'-C46'-H46'	118.7
C51-C52-H52	119.9		C34'-C35'-H35'	120		C48'-C47'-C52'	119.5(11)
C47-C52-H52	119.9		C35'-C36'-C37'	119.2(13)		C48'-C47'-P4'	120.6(10)
C27'-P3'-C33'	104.5(7)		C35'-C36'-H36'	120.4		C52'-C47'-P4'	119.4(10)
C27'-P3'-C39'	104.0(6)		C37'-C36'-H36'	120.4		C49'-C48'-C47'	120.0(12)
C33'-P3'-C39'	104.4(6)		C36'-C37'-C38'	121.1(13)		C49'-C48'-H48'	120
C27'-P3'-Au1	125.6(5)		C36'-C37'-H37'	119.5		C47'-C48'-H48'	120
C33'-P3'-Au1	114.5(5)		C38'-C37'-H37'	119.5		C48'-C49'-C50'	119.6(12)
C39'-P3'-Au1	101.4(4)		C33'-C38'-C37'	121.4(13)		C48'-C49'-H49'	120.2
C41'-P4'-C47'	102.4(8)		C33'-C38'-H38'	119.3		C50'-C49'-H49'	120.2
C41'-P4'-C40'	105.0(7)		C37'-C38'-H38'	119.3		C51'-C50'-C49'	121.2(11)
C47'-P4'-C40'	100.2(7)		C40'-C39'-P3'	111.3(6)		C51'-C50'-H50'	119.4
C41'-P4'-Au1	119.0(7)		C40'-C39'-H39C	109.4		C49'-C50'-H50'	119.4
C47'-P4'-Au1	121.5(6)		P3'-C39'-H39C	109.4		C50'-C51'-C52'	118.4(11)
C40'-P4'-Au1	106.0(4)		C40'-C39'-H39D	109.4		C50'-C51'-H51'	120.8

C52'-C51'-H51'	120.8
C51'-C52'-C47'	121.3(11)
C51'-C52'-H52'	119.4
C47'-C52'-H52'	119.4
O3-Cl1-O4	109.9(11)
O3-Cl1-O1	108.3(10)
O4-Cl1-O1	110(1)
O3-Cl1-O2	109.8(11)
O4-Cl1-O2	108.5(13)
O1-Cl1-O2	110.2(11)
O3'-Cl1'-O2'	109.1(13)
O3'-Cl1'-O4'	109(1)
O2'-Cl1'-O4'	108.8(12)
O3'-Cl1'-O1'	110.8(10)
O2'-Cl1'-O1'	110.2(10)
O4'-Cl1'-O1'	108.9(10)
N1S-C1S-C2S	177.5(6)
C1S-C2S-H2SA	109.5
C1S-C2S-H2SB	109.5
H2SA-C2S-H2SB	109.5
C1S-C2S-H2SC	109.5
H2SA-C2S-H2SC	109.5
H2SB-C2S-H2SC	109.5

Table S9. Optimized XYZ coordinates (Energy in hartree)

<Complex 1>

C	-1.93032500	0.71810900	0.10366200
C	-4.25219000	0.70581800	0.08376900
C	-4.25160800	-0.70794600	-0.08511500
C	-1.92971900	-0.71849400	-0.10380400
H	-5.45890500	2.48290700	0.28768000
C	-5.48289800	1.40557200	0.16133200
C	-5.48174700	-1.40863000	-0.16330300
C	-6.66419600	-0.70770600	-0.08140000
C	-6.66477400	0.70375600	0.07880900
H	-5.45687200	-2.48594600	-0.28963500
H	-7.61144600	-1.23473600	-0.14122200
H	-7.61245400	1.23006700	0.13813500
N	-3.07356400	-1.37636500	-0.18082100
N	-3.07470300	1.37512400	0.18010600
C	-1.12284400	-3.05426600	-1.57485900
H	-1.18678400	-2.53846500	-2.53564700
H	-0.40885000	-3.87516000	-1.67910700
H	-2.10474500	-3.43199600	-1.29268100
C	-0.32600600	2.85756800	-1.30327200
C	-1.43118400	3.92864600	-1.38427500
H	-1.32116200	4.69657800	-0.61368100
H	-1.35809900	4.42918400	-2.35658200
H	-2.43201200	3.49628400	-1.30176900
C	-0.44599000	1.91023800	-2.51095600
H	-0.29449500	2.49058700	-3.42816500
H	0.31179300	1.12228200	-2.49587900
H	-1.43759800	1.44979400	-2.57253800
C	1.06369200	3.52746900	-1.30390300
H	1.86944800	2.78880500	-1.32317600
H	1.15492800	4.15229200	-2.20003800
H	1.20915200	4.17733000	-0.43418100
C	-0.32467200	-2.85636100	1.30448500

C	-1.12555000	3.05401200	1.57572900
C	-0.44772500	-1.90914900	2.51196100
H	-0.29602700	-2.48915300	3.42935300
H	0.30855400	-1.11976000	2.49765000
H	-1.44028100	-1.45062300	2.57251100
C	-1.42829200	-3.92915200	1.38438600
H	-1.31613700	-4.69714200	0.61416200
H	-1.35565800	-4.42928600	2.35693300
H	-2.42967500	-3.49831300	1.30052900
C	1.06608000	-3.52406800	1.30691000
H	1.21365200	-4.17368100	0.43735900
H	1.87064100	-2.78412800	1.32724100
H	1.15712200	-4.14875600	2.20316100
H	-0.41228200	3.87549600	1.68029000
H	-1.18930700	2.53799300	2.53641200
H	-2.10770300	3.43096500	1.29337000
Au	1.45691400	0.95762700	1.21703200
Au	1.45832100	-0.95713300	-1.21693000
Cl	3.28599300	-0.15519700	-2.42724400
Cl	3.28510700	0.15432000	2.42573800
P	-0.45941600	1.86963800	0.32652600
P	-0.45788000	-1.86906400	-0.32578000

Sum of electronic and zero-point Energies= -2686.726910

Sum of electronic and thermal Enthalpies= -2686.692907

Sum of electronic and thermal Free Energies= -2686.794036

<Complex 2>

C	-0.16279800	3.44904600	0.26415800
N	-0.70308100	4.65274800	0.33676300
N	1.66233200	4.21863700	-1.09876900
C	-0.08768800	5.68553500	-0.29807200
C	1.03464600	3.21893900	-0.50429900
C	1.13227000	5.46803000	-1.01142300
C	-0.64677700	6.98777500	-0.24280900

H	2.70631800	6.37723700	-2.17443200
C	0.00000000	8.03007300	-0.86739000
H	-1.57558200	7.12807000	0.30011600
H	-0.41981200	9.03030200	-0.82735100
C	1.21855100	7.81650500	-1.56634700
H	1.70797500	8.65780000	-2.04703100
C	1.77933100	6.56151500	-1.64169100
P	1.73452100	1.50320300	-0.73096900
P	-1.01942800	2.08421200	1.21070000
C	3.28083400	1.62835900	0.28282800
H	3.89794100	0.73952700	0.12816600
H	3.00679100	1.67669700	1.33907600
H	3.85445100	2.51926900	0.01774400
C	2.35275100	1.39042100	-2.53344000
C	3.59425700	2.25718300	-2.81828500
H	3.86829800	2.14477100	-3.87412600
H	4.45856300	1.94160300	-2.22649800
H	3.40218400	3.31215500	-2.62096000
C	2.70964900	-0.09392700	-2.75984300
H	3.02937400	-0.23581100	-3.79845700
H	1.85607400	-0.75242300	-2.57308300
H	3.53891200	-0.41655600	-2.12050000
C	1.20116700	1.79531000	-3.47133400
H	1.49634000	1.60775100	-4.50991300
H	0.96180100	2.85803300	-3.38171500
H	0.29280600	1.21619000	-3.27766500
C	-0.88743300	2.60501800	3.04712100
C	0.60502200	2.72218100	3.40754100
H	1.08709500	3.54936400	2.87682100
H	1.15418400	1.80131300	3.18579100
H	0.70759600	2.91814600	4.48086100
C	-1.53758200	1.47321700	3.86877400
H	-1.05488400	0.50849900	3.69165100
H	-2.60482300	1.36666200	3.64702500

H	-1.45079500	1.70392300	4.93666400
C	-1.60374800	3.93206100	3.35908000
H	-1.21326500	4.76172900	2.76771700
H	-1.46222000	4.16910100	4.42023700
H	-2.68120700	3.86730200	3.18222300
C	-2.78203700	2.43017300	0.76858000
H	-2.95700100	2.12207400	-0.26472400
H	-2.99525600	3.49633700	0.86283100
H	-3.44650400	1.85753100	1.42059900
Au	0.00000000	0.00000000	0.27539600
H	-1.70797500	-8.65780000	-2.04703100
C	-1.21855100	-7.81650500	-1.56634700
H	-2.70631800	-6.37723700	-2.17443200
C	-1.77933100	-6.56151500	-1.64169100
C	0.64677700	-6.98777500	-0.24280900
C	-1.13227000	-5.46803000	-1.01142300
C	0.00000000	-8.03007300	-0.86739000
C	0.08768800	-5.68553500	-0.29807200
N	-1.66233200	-4.21863700	-1.09876900
H	0.41981200	-9.03030200	-0.82735100
H	1.57558200	-7.12807000	0.30011600
C	-1.03464600	-3.21893900	-0.50429900
C	0.16279800	-3.44904600	0.26415800
N	0.70308100	-4.65274800	0.33676300
P	1.01942800	-2.08421200	1.21070000
P	-1.73452100	-1.50320300	-0.73096900
C	-3.28083400	-1.62835900	0.28282800
H	-3.85445100	-2.51926900	0.01774400
H	-3.89794100	-0.73952700	0.12816600
H	-3.00679100	-1.67669700	1.33907600
C	-2.35275100	-1.39042100	-2.53344000
C	-2.70964900	0.09392700	-2.75984300
H	-3.02937400	0.23581100	-3.79845700
H	-1.85607400	0.75242300	-2.57308300

H	-3.53891200	0.41655600	-2.12050000
C	-1.20116700	-1.79531000	-3.47133400
H	-1.49634000	-1.60775100	-4.50991300
H	-0.96180100	-2.85803300	-3.38171500
H	-0.29280600	-1.21619000	-3.27766500
C	-3.59425700	-2.25718300	-2.81828500
H	-3.86829800	-2.14477100	-3.87412600
H	-4.45856300	-1.94160300	-2.22649800
H	-3.40218400	-3.31215500	-2.62096000
C	2.78203700	-2.43017300	0.76858000
H	2.95700100	-2.12207400	-0.26472400
H	2.99525600	-3.49633700	0.86283100
H	3.44650400	-1.85753100	1.42059900
C	0.88743300	-2.60501800	3.04712100
C	1.53758200	-1.47321700	3.86877400
H	2.60482300	-1.36666200	3.64702500
H	1.45079500	-1.70392300	4.93666400
H	1.05488400	-0.50849900	3.69165100
C	1.60374800	-3.93206100	3.35908000
H	2.68120700	-3.86730200	3.18222300
H	1.21326500	-4.76172900	2.76771700
H	1.46222000	-4.16910100	4.42023700
C	-0.60502200	-2.72218100	3.40754100
H	-1.08709500	-3.54936400	2.87682100
H	-1.15418400	-1.80131300	3.18579100
H	-0.70759600	-2.91814600	4.48086100

Sum of electronic and zero-point Energies= -3124.883891

Sum of electronic and thermal Enthalpies= -3124.828609

Sum of electronic and thermal Free Energies= -3124.971890

<Complex 3>

H	-5.58009100	-3.47986500	1.35546300
C	-4.76477500	-2.78536500	1.17932700
H	-5.34393700	-2.29494100	-0.85902800

C	-4.64429300	-2.13435500	-0.04654400
C	-3.60001300	-1.22837700	-0.23715800
C	-3.82614700	-2.52317300	2.18011300
C	-3.55970500	-0.42215300	-1.52010300
C	-3.25636700	1.03455400	-1.39061300
C	-4.05237400	1.87421700	-2.19594600
C	-2.37476000	1.60568900	-0.45329900
H	-1.73842800	3.44831500	0.47463100
C	-4.03576500	3.25267600	-2.01427700
H	-4.70596000	1.41090300	-2.92831600
H	-4.66949100	3.88920400	-2.62291800
C	-3.22177400	3.80339700	-1.02516600
H	-3.22858500	4.87424200	-0.84441200
C	-2.37486100	2.98532300	-0.26469000
O	-3.94975100	-0.94150900	-2.54800700
H	-2.01545000	-1.43642800	2.64674600
C	-2.78119300	-1.64826600	1.90955500
N	-2.67614100	-1.01852200	0.72000000
Au	-0.96717100	0.22066300	0.30359500
H	-3.89214200	-2.99121500	3.15610100
C	2.32495600	-0.79094900	0.10923200
N	3.42697100	-1.51844400	0.05829500
N	3.43466200	1.20154700	-0.64626000
C	4.57308900	-0.92178100	-0.35974900
C	2.34510100	0.59735200	-0.20943600
C	4.56918800	0.46447900	-0.75921000
C	5.77679200	-1.66602700	-0.42897300
H	5.73803100	2.10190100	-1.54338600
C	6.92004600	-1.05610700	-0.89532000
H	5.76573100	-2.70521900	-0.11872000
H	7.84595400	-1.61892900	-0.95611500
C	6.91096800	0.30636200	-1.30594500
H	7.82988100	0.75314500	-1.67166600
C	5.76065300	1.06049500	-1.24121100

P	0.89744900	1.68589200	0.11727700
P	0.73727600	-1.63375300	0.52184700
C	1.01856000	-2.42318900	2.15942400
H	1.07675200	-1.66104900	2.93958300
H	1.96194800	-2.97407500	2.13085600
H	0.20543900	-3.11483400	2.39233800
C	0.87055800	2.87423400	-1.27069900
H	1.89833700	3.20396300	-1.44154400
H	0.22932500	3.72556400	-1.04286300
H	0.49567100	2.37615600	-2.16745300
C	1.28782400	2.57733700	1.76423800
C	1.60249500	1.50833400	2.82813700
H	1.84179500	2.01444600	3.76858200
H	2.46528600	0.89092600	2.56444900
H	0.74181700	0.85882100	3.02276700
C	0.09584100	3.43123600	2.23166100
H	-0.80631500	2.83892200	2.41083300
H	-0.13247900	4.24302200	1.53598900
H	0.36897100	3.90092600	3.18221600
C	2.51318700	3.48923700	1.53431700
H	2.76321200	3.95819800	2.49206100
H	2.30125300	4.29379600	0.82516700
H	3.39237600	2.94369500	1.18792500
C	0.51129600	-3.01831700	-0.77243400
C	-0.88320600	-3.65092400	-0.60130200
H	-1.06032700	-4.01841800	0.41445200
H	-0.95006500	-4.51610000	-1.26880400
H	-1.68676000	-2.96758900	-0.88059600
C	1.58767100	-4.10608400	-0.57085300
H	1.45675000	-4.64225300	0.37328600
H	2.60148100	-3.70668800	-0.60904500
H	1.47764100	-4.83976400	-1.37686500
C	0.63386700	-2.38428500	-2.17179800
H	1.63554700	-1.98966300	-2.36168900

H	-0.09719800	-1.58340700	-2.33315700
H	0.43804800	-3.15357800	-2.92558300

Sum of electronic and zero-point Energies= -2221.806385

Sum of electronic and thermal Enthalpies= -2221.767187

Sum of electronic and thermal Free Energies= -2221.875245

<Complex 4>

P	1.99356600	-2.53416100	-0.06713500
C	-0.80169300	-2.87237100	-0.49616200
Au	-2.20875700	0.17585800	0.35819600
P	-2.43208100	-2.17627000	0.08168100
Au	2.20486900	-0.19042900	-0.40363800
C	0.28561400	-2.88633800	0.59680700
H	-0.51688900	-2.24272800	-1.34586600
H	0.09670400	-2.11829100	1.35409200
H	-0.95969600	-3.87600100	-0.90059200
H	0.29787600	-3.84789500	1.11716500
H	1.07546900	3.82820600	0.95043600
C	0.85693200	2.85097800	0.51127800
P	-1.96998700	2.54144500	0.29356600
P	2.40381300	2.17612800	-0.28453000
C	-0.32448500	2.96752500	-0.47583200
H	-0.20930300	2.29096200	-1.32845800
H	0.62957800	2.17377300	1.34077600
H	-0.37495400	3.98142800	-0.88208800
C	3.78777900	2.64941200	0.80999000
C	5.97319400	3.24297600	2.45805700
C	3.58623400	2.97383400	2.16096300
C	5.09516500	2.61490400	0.29325300
C	6.17955700	2.91426500	1.11568200
C	4.67850700	3.27180000	2.97785600
H	2.58854500	3.00299100	2.58719100
H	5.26717200	2.37022700	-0.75098600
H	7.18494300	2.89423100	0.70717200

H	4.51461200	3.53129100	4.01903500
H	6.81953000	3.47807300	3.09563800
C	2.62944800	3.07033000	-1.85643200
C	2.91696200	4.45041400	-4.27353900
C	2.43525000	2.40560400	-3.07748800
C	2.97342800	4.43394900	-1.85449900
C	3.11409500	5.11778100	-3.06059000
C	2.57902800	3.09634600	-4.28218000
H	2.18308300	1.34855100	-3.08547000
H	3.14903800	4.95557900	-0.91810000
H	3.38537100	6.16876600	-3.05533900
H	2.43698300	2.57594900	-5.22421700
H	3.03547100	4.98554400	-5.21054800
C	2.22785900	-3.60442900	-1.52179900
C	2.56288600	-5.24805300	-3.76195900
C	2.16453300	-5.00354500	-1.38821000
C	2.46460500	-3.03930000	-2.78426200
C	2.63256700	-3.86142000	-3.90029800
C	2.32940400	-5.81818300	-2.50635200
H	2.00633400	-5.45772100	-0.41398800
H	2.52733600	-1.96000600	-2.89251600
H	2.82356300	-3.41936300	-4.87307800
H	2.28384900	-6.89730400	-2.39852400
H	2.69692400	-5.88693700	-4.62923700
C	3.15194700	-3.10857200	1.22563200
C	4.99655500	-3.86717700	3.19195600
C	2.81791200	-3.00619200	2.58652100
C	4.42198900	-3.58363000	0.85827900
C	5.33646200	-3.96243900	1.84093400
C	3.73795300	-3.38939700	3.56263400
H	1.84523100	-2.63632600	2.89685300
H	4.69435600	-3.67152600	-0.18882400
H	6.31287700	-4.33601900	1.54908900
H	3.46907100	-3.31837900	4.61191900

H	5.70952400	-4.16624700	3.95374400
C	-3.61101800	-2.57308500	-1.25839100
C	-5.35960300	-3.13865900	-3.36948200
C	-3.48332600	-1.89740300	-2.48518800
C	-4.62802500	-3.52481600	-1.09386600
C	-5.49841200	-3.80204900	-2.14988800
C	-4.35023200	-2.18650000	-3.53695500
H	-2.71262900	-1.14204500	-2.62025800
H	-4.74745400	-4.04555600	-0.15011800
H	-6.28598500	-4.53676900	-2.01561800
H	-4.24506200	-1.66399700	-4.48262600
H	-6.03947900	-3.35751700	-4.18681200
C	-2.91208600	-3.10299200	1.57334700
C	-3.72508100	-4.50492400	3.85821900
C	-3.54472000	-2.42952600	2.63089900
C	-2.69218000	-4.48884100	1.67055300
C	-3.09622800	-5.18304600	2.81038400
C	-3.95111400	-3.13040900	3.76701300
H	-3.72642200	-1.36036000	2.56309700
H	-2.22038700	-5.03312900	0.85737600
H	-2.92532800	-6.25278000	2.87880200
H	-4.44424600	-2.60399100	4.57806400
H	-4.04062900	-5.04915100	4.74280300
C	-2.04429300	3.40733900	1.89588900
C	-2.09856100	4.73414700	4.35915500
C	-2.00883800	2.67613600	3.09371000
C	-2.11028400	4.81167700	1.94073500
C	-2.13547100	5.46836200	3.16955300
C	-2.03570200	3.34051400	4.32144800
H	-1.96841500	1.59041300	3.06640000
H	-2.15971600	5.39029500	1.02270800
H	-2.19155400	6.55193200	3.20010900
H	-2.01617600	2.77040000	5.24501400
H	-2.12612600	5.25003400	5.31387900

C	-3.23480400	3.29339500	-0.79019300
C	-5.25126900	4.32465200	-2.43820300
C	-2.98277200	3.53184100	-2.15116100
C	-4.50931900	3.56633200	-0.26371200
C	-5.50915800	4.08244400	-1.08673300
C	-3.98968600	4.04893800	-2.96782200
H	-2.00860000	3.32749300	-2.58426400
H	-4.71824100	3.39248600	0.78767900
H	-6.48806300	4.29885700	-0.67082500
H	-3.78465400	4.24149100	-4.01626100
H	-6.03097300	4.72948200	-3.07556900

Sum of electronic and zero-point Energies= -3646.314319

Sum of electronic and thermal Enthalpies= -3646.256674

Sum of electronic and thermal Free Energies= -3646.423152

<Complex 5>

Au	0.00011100	-0.00004100	0.00188600
P	-1.37560800	1.00549600	-1.87725800
C	-0.28832700	0.71220800	-3.37272900
C	0.28690700	-0.71151200	-3.37296700
P	1.37463000	-1.00512600	-1.87788400
H	-0.87449100	0.88572900	-4.28051100
H	0.52380100	1.44536500	-3.36150800
H	0.87282900	-0.88483800	-4.28094300
H	-0.52521400	-1.44467300	-3.36167100
P	1.02572700	1.36141900	1.87910200
P	-1.02502100	-1.36157700	1.87936300
C	0.71592200	0.27796600	3.37392200
H	0.89791100	0.86048200	4.28233700
H	1.43875200	-0.54341300	3.36141400
C	-0.71500400	-0.27804200	3.37407300
H	-1.43783900	0.54333600	3.36164500
H	-0.89682100	-0.86049100	4.28256300
C	1.76802900	-2.80149900	-1.97154300

C	2.33499300	-5.55820300	-1.95333100
C	3.05134700	-3.26643300	-1.63730200
C	0.76854300	-3.74131000	-2.27667400
C	1.05297900	-5.10703400	-2.27298400
C	3.33123900	-4.63410300	-1.63210700
H	3.83969100	-2.56026900	-1.39645500
H	-0.23902800	-3.41786600	-2.51948000
H	0.27141700	-5.81819100	-2.52327800
H	4.33270200	-4.97491700	-1.38583100
H	2.55584100	-6.62120500	-1.95610900
C	2.93060500	-0.16089700	-2.38494400
C	5.23678300	1.22490200	-3.18386900
C	3.64788600	-0.56765600	-3.52524700
C	3.38451100	0.94518200	-1.65461400
C	4.53319000	1.63503000	-2.05242200
C	4.79266400	0.12166100	-3.91997100
H	3.32100000	-1.43058700	-4.09850400
H	2.84223100	1.26445500	-0.77153900
H	4.87589700	2.48397200	-1.46925700
H	5.33971100	-0.20184600	-4.80052300
H	6.13062100	1.75829800	-3.49348100
C	-1.76915600	2.80184400	-1.97043800
C	-2.33637800	5.55848000	-1.95127800
C	-0.76991700	3.74181600	-2.27583800
C	-3.05235500	3.26656800	-1.63542100
C	-3.33237800	4.63420400	-1.62976200
C	-1.05448200	5.10751500	-2.27167800
H	0.23755900	3.41850800	-2.51922200
H	-3.84049900	2.56025800	-1.39432900
H	-4.33374600	4.97486700	-1.38289400
H	-0.27311000	5.81880500	-2.52218500
H	-2.55733300	6.62146000	-1.95369300
C	-2.93172400	0.16134900	-2.38399600
C	-5.23823000	-1.22423800	-3.18231400

C	-3.38544300	-0.94478600	-1.65363600
C	-3.64937300	0.56829500	-3.52400300
C	-4.79430700	-0.12092300	-3.91842900
C	-4.53428800	-1.63453700	-2.05114600
H	-2.84289300	-1.26417500	-0.77076800
H	-3.32265800	1.43130800	-4.09723800
H	-5.34164600	0.20271900	-4.79875100
H	-4.87684300	-2.48353200	-1.46797000
H	-6.13218900	-1.75755700	-3.49170900
C	0.21251900	2.93395200	2.38383500
C	-1.12358400	5.27357400	3.16988400
C	-0.86231800	3.42590300	1.63174800
C	0.61324000	3.63013700	3.53933300
C	-0.05144500	4.79146100	3.92777400
C	-1.52740900	4.59132000	2.02319500
H	-1.17734700	2.89950600	0.73752400
H	1.45294400	3.27419200	4.12959300
H	0.26705000	5.32184100	4.82026400
H	-2.35282500	4.96248900	1.42390900
H	-1.63798300	6.18007400	3.47479900
C	2.83054100	1.71588100	1.97247000
C	5.59981100	2.21761200	1.95805000
C	3.32424700	2.99795900	1.67797900
C	3.74777800	0.68502700	2.24057200
C	5.11970800	0.93705700	2.23919100
C	4.69840600	3.24514400	1.67425000
H	2.63625100	3.81026200	1.46650600
H	3.40163200	-0.32213500	2.45243000
H	5.81323500	0.13133000	2.46074600
H	5.06194500	4.24572700	1.45874400
H	6.66778300	2.41293700	1.96167300
C	-0.21129300	-2.93393100	2.38387000
C	1.12557500	-5.27327100	3.16948400
C	-0.61123200	-3.62998900	3.53971300

C	0.86314600	-3.42588100	1.63121500
C	1.52861200	-4.59115700	2.02243300
C	0.05383700	-4.79116400	3.92794400
H	-1.45064700	-3.27406900	4.13040000
H	1.17756600	-2.89960400	0.73671000
H	2.35369600	-4.96232700	1.42269200
H	-0.26405800	-5.32144000	4.82070900
H	1.64027100	-6.17965700	3.47424200
C	-2.82970100	-1.71657000	1.97329000
C	-5.59881400	-2.21921600	1.95970700
C	-3.74719900	-0.68603200	2.24174000
C	-3.32307900	-2.99878400	1.67884500
C	-4.69715800	-3.24642700	1.67554100
C	-5.11904500	-0.93851800	2.24077500
H	-3.40133500	0.32123500	2.45357200
H	-2.63487500	-3.81082900	1.46707000
H	-5.06043200	-4.24711300	1.46006800
H	-5.81277100	-0.13303800	2.46260200
H	-6.66672100	-2.41489500	1.96366200

Sum of electronic and zero-point Energies= -3510.722142

Sum of electronic and thermal Enthalpies= -3510.667164

Sum of electronic and thermal Free Energies= -3510.821214

<Complex 6>

C	-1.61511400	-1.39976300	1.26380900
C	-1.50783800	-2.78919900	1.09127100
C	-2.59084000	-3.61853200	1.44220800
C	-3.73208000	-3.07545000	2.02325700
C	-3.80570600	-1.69991300	2.24595300
C	-2.76216600	-0.85776600	1.84055000
H	-2.49725200	-4.68741900	1.27868000
H	-4.55327700	-3.72254500	2.31358600
H	-4.68071100	-1.27059500	2.72489900
H	-2.86523100	0.21055300	1.99289200

C	-0.26715300	-3.49634600	0.66171400
O	-0.27409200	-4.55217800	0.05637600
C	1.05671400	-2.98961600	1.21081500
C	1.99494300	-3.93878600	1.61842100
C	2.40694600	-1.25510400	2.00750900
C	3.15422500	-3.52071000	2.26872600
H	1.78168300	-4.98482300	1.43056000
C	3.35177500	-2.15646600	2.48419000
H	2.54188200	-0.18614900	2.11682000
H	3.88554500	-4.24672200	2.60947700
H	4.22780000	-1.78401300	3.00310900
Au	-0.05544000	-0.26796800	0.43181400
N	1.28565900	-1.66903400	1.38104900
P	-1.56831200	1.17577700	-0.71065400
P	1.73200200	1.02432000	-0.75278800
C	0.80471400	2.45295700	-1.49747300
C	-0.54304900	1.97064700	-2.04028000
H	1.40610900	2.89451100	-2.29754500
H	0.66966200	3.21822100	-0.72742000
H	-0.40664100	1.24562100	-2.84834900
H	-1.12882100	2.80313200	-2.44374000
C	3.08007200	1.72218300	0.25240900
C	5.10589100	2.78495500	1.86754100
C	4.40826200	1.29166400	0.09289200
C	2.77186100	2.67737000	1.24124600
C	3.78387600	3.20711700	2.03962500
C	5.41438700	1.82886900	0.89805600
H	4.66132800	0.55512800	-0.66208700
H	1.75046700	3.01988800	1.38711100
H	3.54323200	3.95251200	2.79090200
H	6.44078600	1.50375200	0.76062400
H	5.89263200	3.20321100	2.48728600
C	-2.11252300	2.52675200	0.37514300
C	-2.88113800	4.70171700	1.96046000

C	-2.96588300	3.51730900	-0.14896000
C	-1.64461600	2.64032700	1.69595700
C	-2.03085600	3.72564500	2.48376000
C	-3.34619200	4.59706300	0.64644400
H	-3.34266300	3.44706200	-1.16519700
H	-0.99079900	1.88115300	2.11696300
H	-1.67343900	3.80404800	3.50551300
H	-4.00740900	5.35539800	0.23971500
H	-3.18378200	5.54298600	2.57580200
C	-2.96597300	0.36851600	-1.52595300
C	-5.08958500	-0.91386700	-2.81171300
C	-4.28084800	0.55253600	-1.06491500
C	-2.71900200	-0.47717800	-2.62501100
C	-3.78109900	-1.11216500	-3.26311600
C	-5.33663700	-0.08423400	-1.71613200
H	-4.48417600	1.19436800	-0.21518100
H	-1.70908900	-0.64741400	-2.98749300
H	-3.58920000	-1.75860400	-4.11323900
H	-6.35241900	0.06970000	-1.36673800
H	-5.91518900	-1.40714000	-3.31473200
C	2.42638800	0.09595300	-2.15553000
C	3.45302500	-1.30102100	-4.35559100
C	2.11798700	-1.26257400	-2.34037600
C	3.25808300	0.74980000	-3.08554800
C	3.76689500	0.05016100	-4.17794000
C	2.63002300	-1.95621900	-3.43837400
H	1.48180100	-1.78564800	-1.63322600
H	3.52105300	1.79590900	-2.95624600
H	4.40876900	0.55893600	-4.88997900
H	2.38674500	-3.00514300	-3.57446300
H	3.85154700	-1.84101300	-5.20859500

Sum of electronic and zero-point Energies= -2414.741453

Sum of electronic and thermal Enthalpies= -2414.702208

Sum of electronic and thermal Free Energies= -2414.816373

<Complex 7>

C	-1.51247800	0.41396000	-0.28180800
C	-1.39681800	1.71269200	0.23728600
C	-2.50369700	2.58588200	0.18339300
C	-3.68287600	2.19040000	-0.43128200
C	-3.77261300	0.91223400	-0.98934300
C	-2.70176500	0.01953800	-0.89846800
H	-2.39563800	3.57434000	0.61748500
H	-4.52580800	2.87259200	-0.48236300
H	-4.68656500	0.59540600	-1.48390700
H	-2.80889600	-0.98701600	-1.28262200
C	-0.15111000	2.29935600	0.79245100
O	-0.15756000	3.24539100	1.56609300
C	1.19339900	1.87315500	0.22695400
C	2.20400200	2.83635700	0.21418900
C	2.57408600	0.36660400	-0.90979600
C	3.41696200	2.55521300	-0.40628400
H	1.99768900	3.78648800	0.69131900
C	3.59533600	1.30580100	-0.99537400
H	2.68824900	-0.64408600	-1.27863500
H	4.20678100	3.29939000	-0.43211100
H	4.51666800	1.03820100	-1.50008300
Au	0.02851600	-0.91734700	0.00790000
N	1.40016300	0.65213400	-0.30988900
Cl	-1.48472900	-2.61778700	0.44691900
Cl	1.87528900	-2.44222000	0.35989400

Sum of electronic and zero-point Energies= -1648.110916

Sum of electronic and thermal Enthalpies= -1648.094694

Sum of electronic and thermal Free Energies= -1648.155296
